# Caspase-3 cleaves CYLD to restrict interferon signaling during mitochondrial apoptosis

**DOI:** 10.1038/s44319-026-00876-4

**Published:** 2026-07-15

**Authors:** Yanis M Macé, Tiphaine Douanne, Jane Jardine, Kilian Trillet, Rosalie Moreau, Joanna Re, Cara J Ellison, Nadine Laguette, Paul R Elliott, Julie Gavard, Nicolas Bidère

**Affiliations:** 1 Team SOAP, CRCI²NA, INSERM, CNRS, Université de Nantes, Nantes, France; 2 Equipe Labellisée Ligue Contre le Cancer, Paris, France; 3 Institut de Génétique Moléculaire de Montpellier (IGMM), Université de Montpellier, CNRS UMR5535, Montpellier, France; 4 Department of Biochemistry, University of Oxford, Oxford, UK; 5 Institut de Cancérologie de l’Ouest (ICO), Saint-Herblain, France

**Keywords:** Autophagy & Cell Death, Immunology, Post-translational Modifications & Proteolysis

## Abstract

Mitochondrial outer membrane permeabilization is a pivotal event in programmed cell death by apoptosis, leading to the activation of the cysteine protease caspase-3 (CASP3) and the release of mitochondrial nucleic acids. This release triggers the activation of the Interferon Regulatory Factor 3 (IRF3) transcription factor and the subsequent IRF3-mediated type I interferon production and cell death. CASP3 ensures apoptosis remains immunologically silent, though the mechanisms are unclear. We report that CASP3 cleaves CYLD, a deubiquitinating enzyme crucial for cell fate and inflammatory signaling. This proteolysis occurs at a site distinct from the previously reported CASP8 site, which is involved in limiting cell lysis and inflammation during extrinsic apoptosis. Although cleaved CYLD retains its enzymatic activity in vitro, knocked-in cells expressing CASP3-resistant CYLD show increased interferon signaling and enhanced cell death. Thus, a proteolytic code regulates CYLD to balance inflammation during programmed cell death.

## Synopsis

Caspase-3 cleaves the deubiquitinating enzyme CYLD, a key enzyme involved in cell death and inflammatory signaling, during mitochondrial apoptosis. This proteolysis prevents dying cells from triggering an inflammatory response and helps maintain apoptosis silence.

- CYLD is differentially cleaved by caspases during apoptosis and pyroptosis.
- Caspase-3 directly cleaves CYLD at Asp364.
- CYLD cleavage by CASP3 tempers IRF3-mediated signaling and cell death.

## Abstract (web summary)

Caspase-3 cleaves the deubiquitinating enzyme CYLD, a key enzyme involved in cell death and inflammatory signaling, during mitochondrial apoptosis. This proteolysis prevents dying cells from triggering an inflammatory response and helps maintain apoptosis silence.

## Graphical abstract

**Figure:**
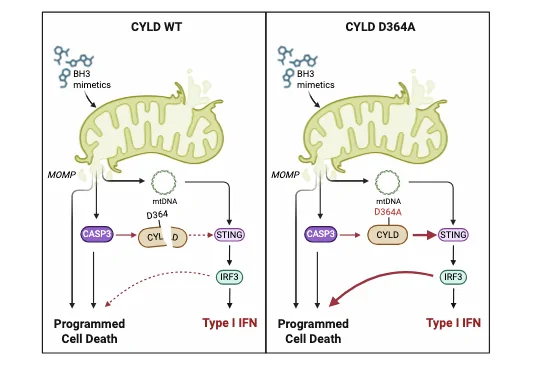

**Figure:**
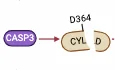

## Introduction

Programmed cell death intricately shapes life, with various modalities coexisting to balance its inflammatory impact. Cell death programs often activate caspases (CASPs), a family of cysteine proteases that regulate cell demise and inflammation (Newton et al, 2024). Mitochondrial apoptosis, a well-studied form of cell death, is typically non-immunogenic, initiated by cellular stress, and controlled by BCL-2 family members (Glover et al, 2024). This pathway involves mitochondrial outer membrane permeabilization (MOMP) and the release of mitochondrial apoptogenic proteins into the cytosol, which in turn activate CASPs alongside a CASP-independent cell death. Cytochrome c released into the cytosol promotes the activation of the initiator CASP9, which subsequently cleaves and activates the executioner CASP3 and CASP7 to dismantle the cell (Newton et al, 2024; Glover et al, 2024).

Mitochondrial permeabilization also liberates mitochondrial nucleic acids (mt-NAs), including mitochondrial DNA (mtDNA), which serves as a potent activator of the transcription factor Interferon Regulatory Factor 3 (IRF3) via the cGAS-STING pathway (Rongvaux et al, 2014; White et al, 2014). IRF3, in turn, drives the production of type I interferons (IFNs) and the subsequent expression of interferon-stimulated genes (ISGs) with microbicidal activities. IRF3 also promotes the transcription of proapoptotic members of the BCL-2 family, thereby enhancing mitochondrial cell death (Gulen et al, 2017; Diepstraten et al, 2024). However, CASP3 suppresses this interferon response to maintain apoptosis immunologically silent (Rongvaux et al, 2014; White et al, 2014), although the precise mechanisms remain unclear. In contrast, CASP1 maturates the inflammatory cytokine IL-18 and pyrogenic cytokine IL-1β and the pore-forming protein Gasdermin D (GSDMD) into biologically active forms to mediate pyroptosis, a highly inflammatory and lytic form of cell death triggered by various infectious and sterile insults (Zhivaki and Kagan, 2021; Muruve et al, 2008).

The tumor suppressor CYLD is a ubiquitin-specific protease (USP)-type deubiquitinating enzyme (DUB) that preferentially hydrolyzes Lys63- and Met1-linked ubiquitin chains from substrates, playing a key role in cell death and inflammatory signaling (Elliott et al, 2021). For instance, CYLD positively regulates TNF-mediated apoptosis and necroptosis, a form of lytic cell death that occurs in apoptosis-deficient conditions (Hitomi et al, 2008; Wang et al, 2008; Wei et al, 2017), while restraining pyroptosis (Yang et al, 2020). CYLD also participates in the induction of type I IFN in response to double-stranded DNA by controlling the activity of the cGAS-STING pathway (Zhang et al, 2018, 2025). However, the role of CYLD in mitochondrial apoptosis or mtDNA-triggered inflammation is unknown. The function and activity of CYLD are shaped by post-translational modifications (PTMs), including phosphorylation and proteolysis (Macé et al, 2025). For instance, CYLD is cleaved and inactivated by CASP8 at residue Asp215 during apoptosis induced by TNF, thereby preventing lytic cell death by necroptosis (O’Donnell et al, 2011). CASP8 also cleaves CYLD at Asp215 in bone marrow-derived macrophages upon TLR4 stimulation (Legarda et al, 2016), and knock-in mice with a D215A point mutation in CYLD resist LPS-induced endotoxic shock (Liu et al, 2026). The paracaspase MALT1 also processes CYLD after the amino acid Arg324 during lymphocyte activation or in a subset of aggressive B-cell lymphoma and glioblastoma-initiating cells (Staal et al, 2011; Douanne et al, 2016; Jacobs et al, 2020). However, the exact role of this proteolysis remains unclear (Gewies et al, 2014). Whether CYLD undergoes additional PTMs during CASP8-independent intrinsic apoptosis is unknown.

## Results and discussion

### CYLD is differentially cleaved during mitochondrial apoptosis and pyroptosis

**Figure 1:**
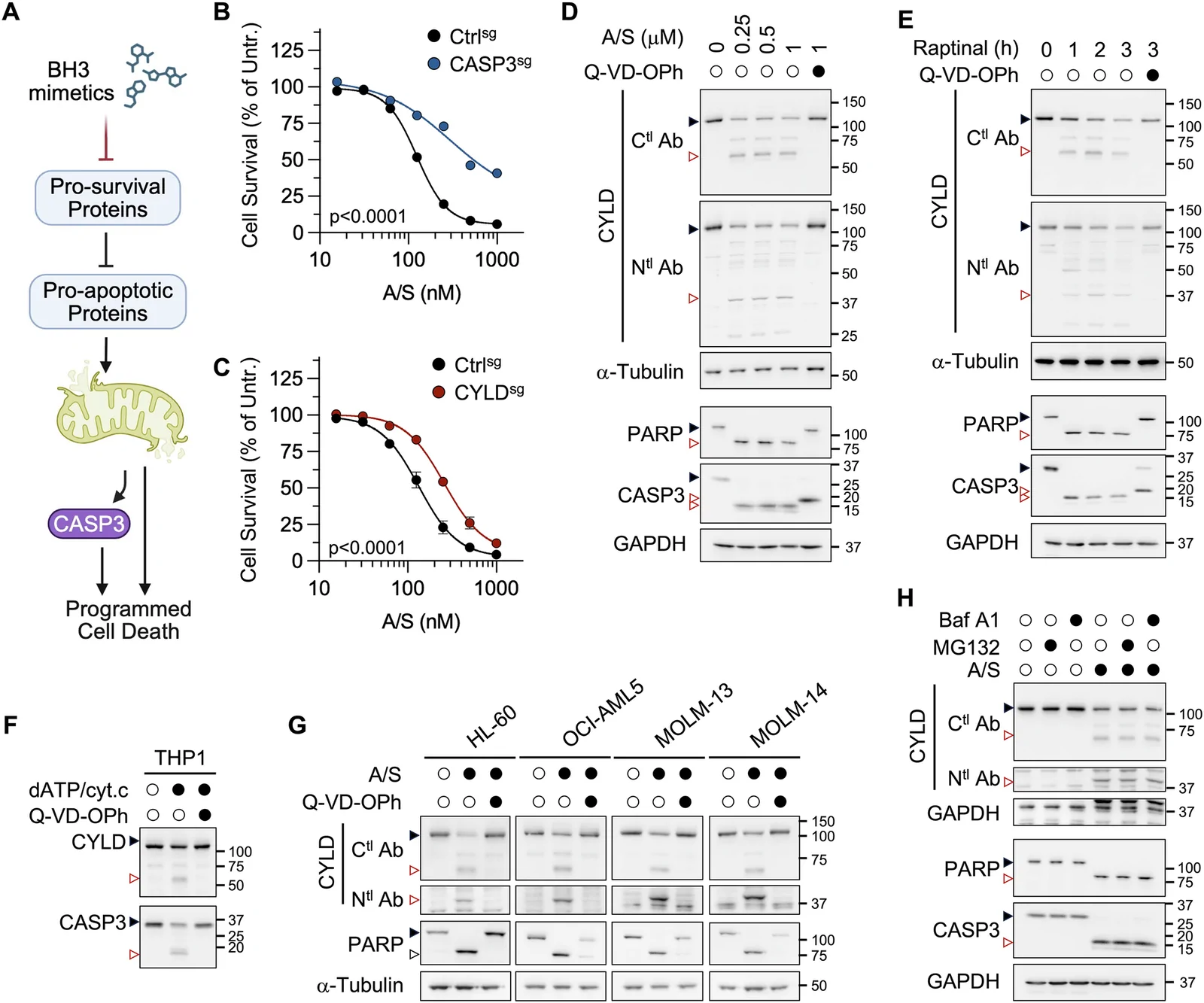
CYLD is cleaved during mitochondrial apoptosis. (**A**) Schematic illustrating mitochondrial outer membrane permeabilization (MOMP), driven by BH3 mimetics or cellular stresses, resulting in CASP-dependent and CASP-independent cell death pathways. (**B**) Dose–response curves of THP-1 cells deleted for CASP3 with a sgRNA (CASP3^sg^) or their control counterparts (Ctrl^sg^) treated with escalating doses of ABT-737 and S63845 (A/S, 15.625, 31.25, 62.5, 125, 250, 500, and 1000 nM) for 1 h. Metabolic cell survival was measured using CellTiter Glo (*n* = 3; mean ± SEM; extra sum-of-squares F test; *P* value < 0.0001). (**C**) Cell survival in Ctrl^sg^ and CYLD^sg^ THP-1 cells treated and analyzed as in (**B**) (*n* = 3; mean ± SEM; extra sum-of-squares F test; *P* value < 0.0001). (**D**) Immunoblot analysis of THP-1 cells pretreated or not with the pan-CASP inhibitor Q-VD-OPh (10 μM, 30 min) and stimulated with A/S as indicated for 1 h. Closed and open triangles indicate full-length and cleaved proteins. Numbers to the right indicate molecular mass markers. (**E**) Cells, as in (**D**), were treated with 10 μM Raptinal as indicated. (**F**) Cell-free extracts prepared from THP-1 cells were incubated with cytochrome c (cytc.c, 50 μg/mL) and dATP (1 mM) for 3 h. When indicated, extracts were preincubated with Q-VD-OPh (10 μM) for 15 min. (**G**) Immunoblot analysis of a panel of human AML cell lines pretreated with Q-VD-OPh (10 μM, 30 min) and exposed to A/S (1 μM each) for 1 h. (**H**) Immunoblot analysis of THP-1 cells pretreated with MG132 (25 μM) or Bafilomycin A1 (BafA1, 200 nM) for 30 min and stimulated with A/S (1 μM each) for 1 h. The presented immunoblots are representative of at least three independent experiments. Source data are available online for this figure.

**Figure EV1:**
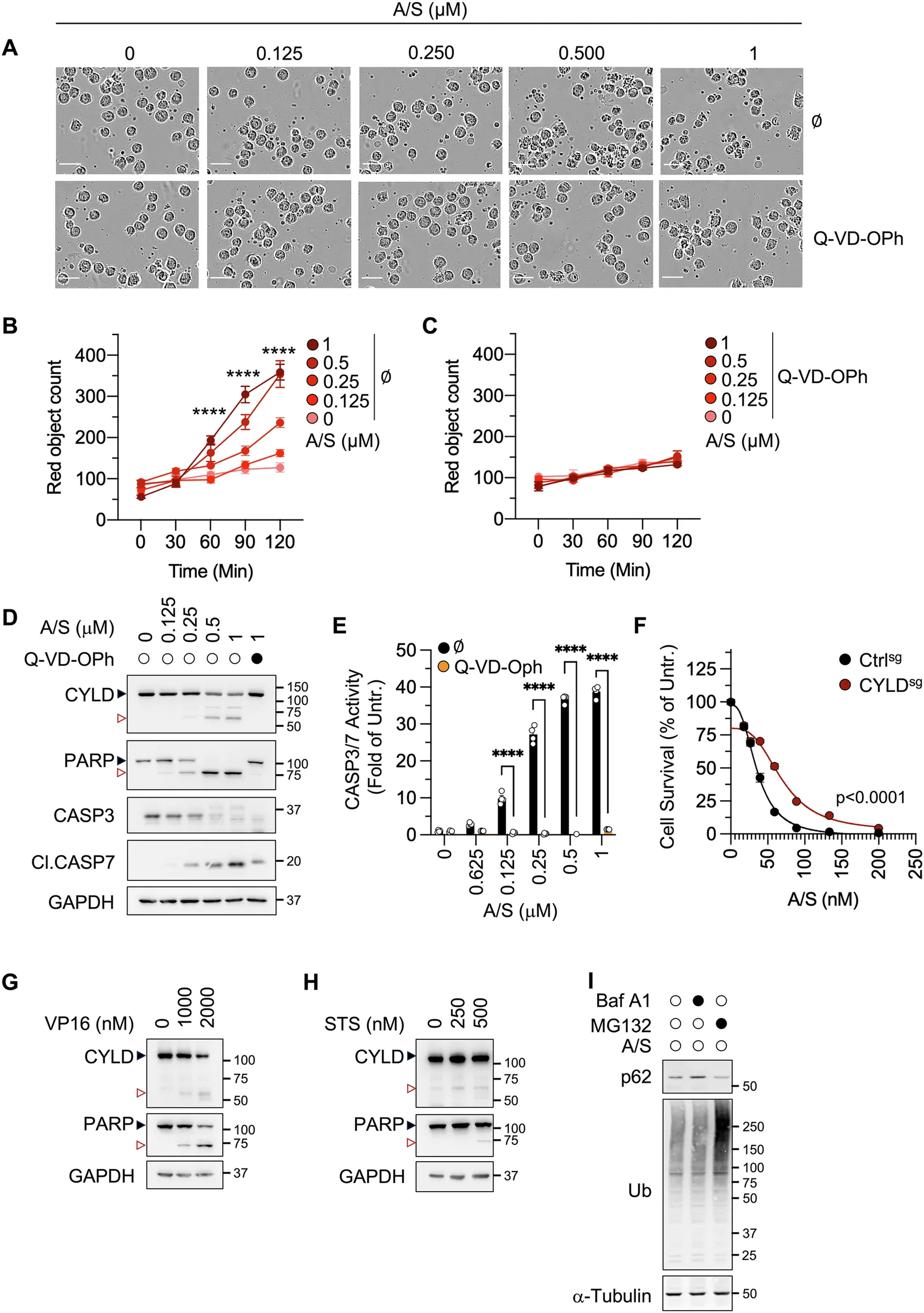
CYLD cleavage is associated with hallmarks of apoptosis. Related to Fig. 1. (**A**) Representative IncuCyte bright-field images of THP-1 cells treated with increasing doses of ABT-737 and S63845 (A/S) for 0 h and 1 h (20X magnification). When indicated, cells were pretreated with the pan-caspase inhibitor Q-VD-OPh (10 µM, 30 min). (**B**, **C**) Real-time quantification of annexin V-positive THP-1 cells (red objects) treated as in (**A**) using IncuCyte (*n* = 3; mean ± SEM; ****P < 0.0001; two-way ANOVA). *P* values for each comparison: 0.125 μM, untreated vs 30 min: *P* = 0.6958; 0.125 μM, untreated vs 60 min: *P* = 0.6395; 0.125 μM, untreated vs 90 min: *P* = 0.0126; 0.125 μM, untreated vs 120 min: *P* < 0.0001; 0.250 μM, untreated vs 30 min: *P* = 0.5936; 0.250 μM, untreated vs 60 min: *P* = 0.1943; 0.250 μM, untreated vs 90 min: *P* = 0.011; 0.250 μM, untreated vs 120 min: *P* < 0.0001; 0.5 μM, untreated vs 30 min: *P* = 0.9921; 0.5 μM, untreated vs 60 min: *P* = 0.0011; 0.5 μM, untreated vs 90 min: *P* < 0.0001; 0.5 μM, untreated vs 120 min: *P* < 0.0001; 1 μM, untreated vs 30 min: *P* = 0.4030; 1 μM, untreated vs 60 min: *P* < 0.0001; 1 μM, untreated vs 90 min: *P* < 0.0001; 1 μM, untreated vs 120 min: *P* < 0.0001. (**D**) Immunoblot analysis of THP-1 cells pretreated or not with Q-VD-OPh (10 μM, 30 min) and stimulated with A/S as indicated for 1 h. Closed and open triangles indicate full-length and cleaved proteins. Cl.CASP7, cleaved CASP7. Numbers to the right indicate molecular mass markers. (**E**) CASP3/7 catalytic activity was measured by luminescent substrate cleavage (CASP Glo assay) in THP-1 cells pretreated or not with Q-VD-OPh (10 μM, 30 min) and treated with escalating concentrations of A/S, as indicated (*n* = 4; mean ± SEM; ****P < 0.0001; two-way ANOVA). *P* values for each comparison: 0.125 μM, control vs Q-VD-Oph: *P* < 0.0001; 0.25 μM, control vs Q-VD-Oph: *P* < 0.0001; 0.5 μM, control vs Q-VD-Oph: *P* < 0.0001; 1 μM, control vs Q-VD-Oph: *P* < 0.0001. (**F**) Dose–response curves of the indicated THP-1 cells treated with escalating doses of ABT-737 and S63845 (A/S) for 24 h (*n* = 6; mean ± SEM; extra sum-of-squares F test; *P* value < 0.0001). (**G**, **H**) Immunoblot analysis of THP-1 cells treated as indicated with etoposide (VP16) or staurosporine (STS) for 24 h. Closed and open triangles indicate full-length and cleaved proteins. (**I**) THP-1 cells were pretreated with MG132 (25 μM) or Bafilomycin A1 (Baf A1, 200 nM) for 30 min and exposed to A/S (1 μM each) for 1 h. Cell lysates were prepared and analyzed by immunoblotting with antibodies specific to the indicated proteins. The presented immunoblots are representative of at least three independent experiments. Source data are available online for this figure.

To assess the status of CYLD during intrinsic apoptosis triggered by mitochondrial permeabilization, THP-1 monocytic cells were challenged with the BH3 mimetics ABT-737 (inhibits BCL-2, BCL-xL, and BCL-w) and S63845 (MCL-1 inhibitor). Such treatment causes the oligomerization of the BCL-2 family members BAX and BAK in the mitochondrial outer membrane, leading to MOMP and the release of mitochondrial proteins and mt-NAs (Riley et al, 2018) (Fig. 1A). Supporting previous findings showing that MOMP causes CASP-dependent and independent cell death (Giampazolias et al, 2017), treatment with ABT-737 and S63845 led to rapid cell death, partially inhibited in cells deleted for CASP3 with single guide RNAs (CASP3^sg^) (Fig. 1B). Of note, the drop in ATP measured with CellTiter Glo correlated with the appearance of morphological changes of apoptosis, activation of CASPs and loss of phosphatidylserine asymmetry (Fig. EV1A–E). In these conditions, the extent of cell death was modestly but significantly reduced in CYLD^sg^ cells, suggesting that CYLD is essentially dispensable for this acute cell death program (Figs. 1C and EV1F). Immunoblotting using an antibody specific to the COOH-terminal region revealed that the abundance of full-length CYLD decreased following treatment. A predominant shorter COOH-terminal fragment with a relative molecular mass of approximately 68 kDa appeared (Fig. 1D). The use of an antibody against the NH_2_-terminal part of CYLD unveiled a main fragment of approximately 40 kDa, suggesting single proteolysis (Fig. 1D). These cleavage fragments were absent in cells treated with the pan-CASP inhibitor Q-VD-OPh, pointing to a cleavage by a CASP (Fig. 1D). Similar results were observed with Raptinal, a BAX/BAK-independent CASP3 activator (Palchaudhuri et al, 2015; Renaud et al, 2023) (Fig. 1E), or with the DNA topoisomerase II inhibitor etoposide and the PKC inhibitor staurosporine (Fig. EV1G,H). CASP activation was also triggered in a cell-free system by adding cytochrome c and dATP to post-nuclear extracts to bypass external stimuli. As expected (Slee et al, 1999, 2001), this caused CASP3 activation and cleavage of CYLD, which were efficiently blocked in the presence of Q-VD-OPh (Fig. 1F). This proteolysis of CYLD was not restricted to THP-1 cells and could be extended to additional Acute Myeloid Leukemia (AML) cell lines and cells from different lineages (Fig. 1G, and see below). Notably, CYLD cleavage fragments did not accumulate in cells treated with proteasome or lysosome inhibitors, indicating stable products (Figs. 1H and EV1I). Hence, CYLD is cleaved during intrinsic apoptosis.

**Figure EV2:**
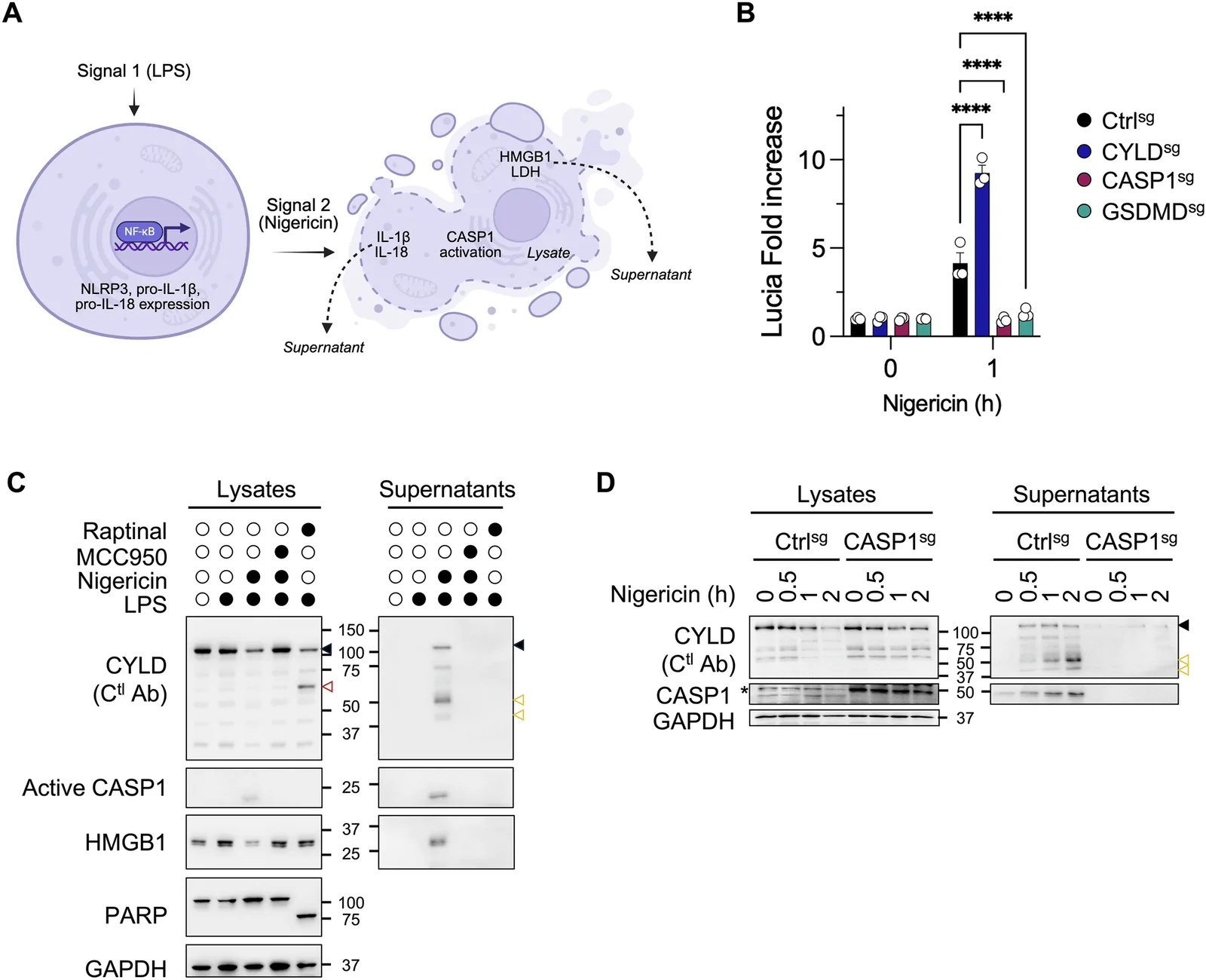
CYLD is differentially cleaved during apoptosis and pyroptosis. Related to Fig. 1. (**A**) Workflow to induce pyroptosis in monocytic cells. (**B**) THP-1 cells stably expressing HMGB1-Lucia deleted with sgRNAs for CYLD (CYLD^sg^), CASP1 (CASP1^sg^), GSDMD (GSDMD^sg^), or their control counterparts (Ctrl^sg^) were primed with LPS (1 μg/mL) for 3 h before stimulation with Nigericin (10 μM) for 1 h. The release of HMGB1-Lucia was assessed in cell supernatants (*n* = 3; mean ± SEM; ****P < 0.0001; two-way ANOVA). *P* values for each comparison: Ctrl^sg^ vs CYLD^sg^: *P* < 0.0001; Ctrl^sg^ vs CASP1^sg^: *P* < 0.0001; Ctrl^sg^ vs GSDMD^sg^: *P* < 0.0001. (**C**) Immunoblot analysis of THP-1 cells primed or not with LPS (1 μg/mL) for 3 h before treatment with Nigericin (10 μM) or Raptinal (10 μM) for 1 h. When indicated, cells were pretreated with the NLRP3 inhibitor MCC950 (2 μM) for 30 min. Closed and open symbols indicate full-length and cleaved CYLD. (**D**) Immunoblot analysis of Ctrl^sg^ and CASP1^sg^ THP-1 cells treated as in (**B**). * indicates a nonspecific band. The presented immunoblots are representative of at least three independent experiments. Source data are available online for this figure.

Given that CYLD also regulates pyroptosis (Yang et al, 2020), we investigated its status during this form of programmed cell death. To this end, THP-1 cells expressing an HMGB1-Lucia fusion protein were primed with LPS before exposure to the microbial toxin Nigericin (Fig. EV2A). This assembles the NLRP3 inflammasome, a cytosolic platform that activates CASP1 (Newton et al, 2021). As expected, this caused plasma membrane rupture (PMR), evidenced by the release in culture supernatants of the cytosolic HMGB1, which was significantly reduced in cells knocked out for the key pyroptosis effectors CASP1 or the pore-forming protein GSDMD (Fig. EV2B). Consistent with Yang et al (Yang et al, 2020), Nigericin-mediated PMR was further enhanced without CYLD (Fig. EV2B). Both full-length CYLD and several lower molecular weight fragments were found in the supernatant of pyroptotic cells, suggesting proteolysis (Fig. EV2C). This proteolysis was efficiently prevented in cells treated with the selective NLRP3 inhibitor MCC950 or in CASP1^sg^ cells (Fig. EV2C,D). Thus, CYLD is differentially cleaved during pyroptosis and intrinsic apoptosis.

### CASP3 mediates CYLD cleavage independent of SPATA2 during mitochondrial apoptosis

**Figure 2:**
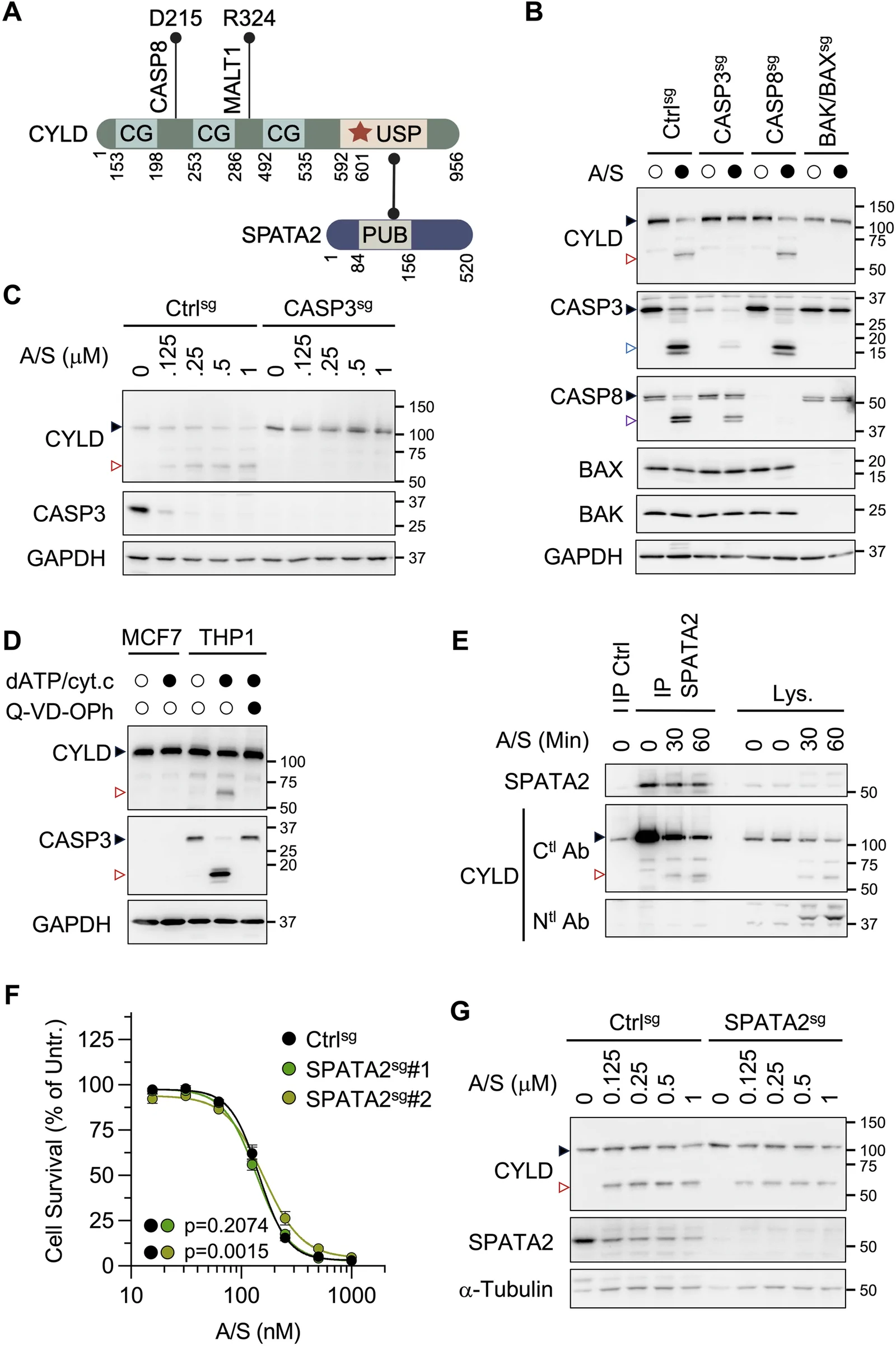
CYLD cleavage during mitochondrial apoptosis requires CASP3. (**A**) Schematic representation of CYLD and SPATA2. Known cleavage sites by CASP8 and MALT1 are indicated. CG, Cap-Gly; USP, Ubiquitin-specific protease. (**B**) Immunoblot analysis of Ctrl^sg^, CASP3^sg^, CASP8^sg^, or BAX/BAK^sg^ MDA-MB-231 cells treated with ABT-737 and S63845 (A/S, 10 μM each) for 2 h. Closed and open triangles indicate full-length and cleaved proteins. (**C**) Immunoblot analysis of Ctrl^sg^ and CASP3^sg^ THP-1 cells exposed to escalating doses of A/S for 1 h. (**D**) Cell-free extracts prepared from THP-1 or MCF7 cells were incubated for 3 h with cytochrome c (cytc.c, 50 μg/mL) and dATP (1 mM). When indicated, extracts were preincubated for 15 min with Q-VD-OPh (10 μM). (**E**) THP-1 cells were treated with A/S (1 μM each) as indicated. Cell lysates were subjected to immunoprecipitation (IP) with antibodies against SPATA2 or with nonrelevant antibodies, and samples were then analyzed by immunoblotting. (**F**) Dose–response curves of Ctrl^sg^ and SPATA2^sg^ THP-1 cells treated with escalating doses of A/S (15.625, 31.25, 62.5, 125, 250, 500, and 1000 nM) for 1 h. Metabolic cell survival was assessed using CellTiter Glo (*n* = 4; mean ± SEM; extra sum-of-squares F test; *P* values for each comparison: Ctrl^sg^ vs SPATA2^sg#1^: *P* = 0.2074, Ctrl^sg^ vs SPATA2^sg#2^: *P* = 0.0015). (**G**) Immunoblot analysis of Ctrl^sg^ and SPATA2^sg^ THP-1 cells treated with escalating doses of A/S for 1 h. The presented immunoblots are representative of at least three independent experiments. Source data are available online for this figure.

**Figure EV3:**
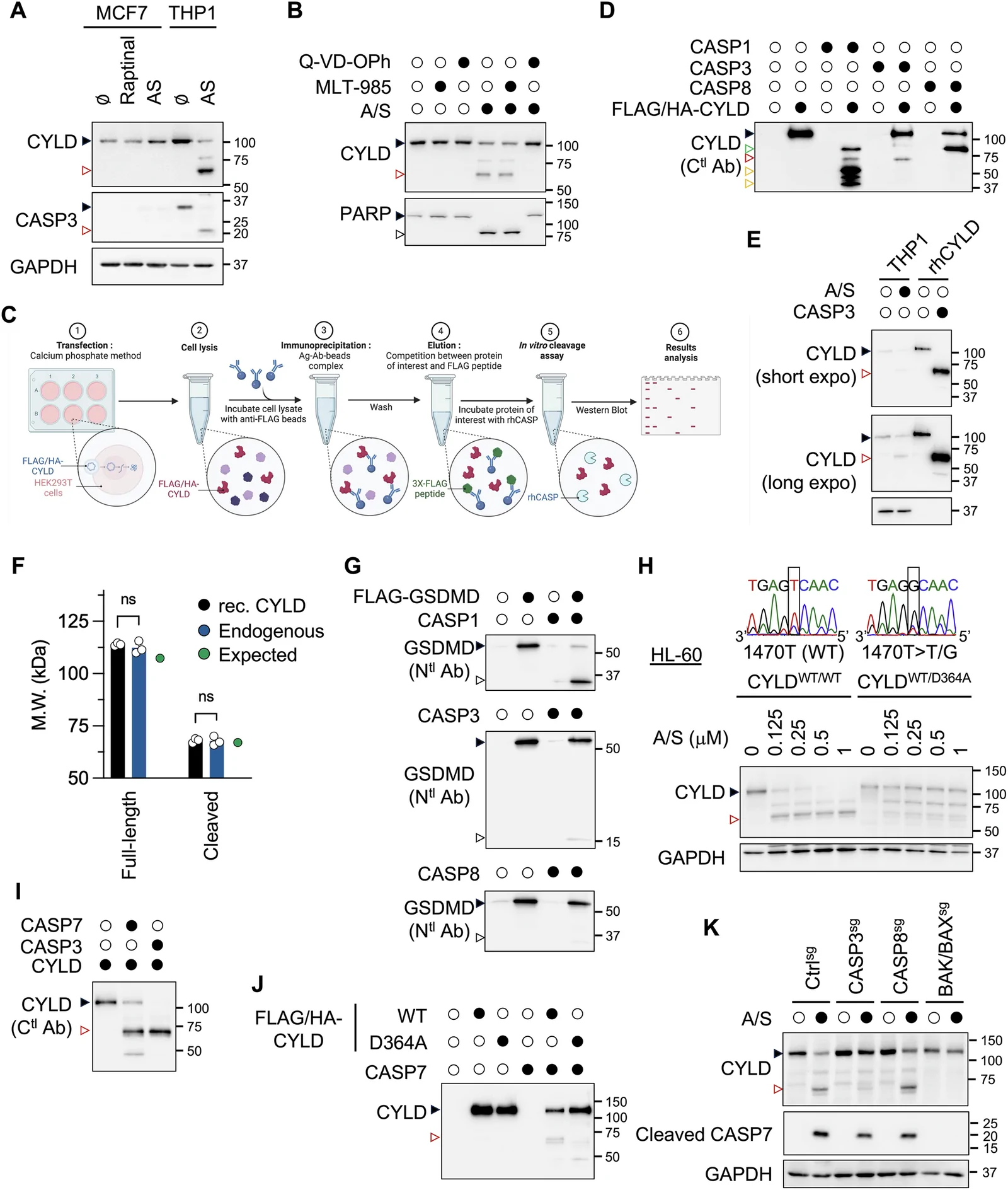
CYLD cleavage by caspases. Related to Figs. 2 and 3. (**A**) Immunoblot analysis of MCF7 cells treated with Raptinal (10 μM) or ABT-737 and S63845 (A/S, 10 μM each) for 3 h. As a control, THP-1 cells exposed to A/S (1 μM each, 1 h) were used. Cell lysates were prepared, and immunoblotting was performed as indicated. Closed and open triangles indicate full-length and cleaved proteins. Numbers to the right indicate molecular mass markers. (**B**) Immunoblot analysis of THP-1 cells pretreated with MLT-985 (2 μM) or Q-VD-OPh (10 μM) for 30 min before exposure to A/S (1 μM each) for 1 h. (**C**) Workflow of the in vitro CASP cleavage assay of immunopurified CYLD. (**D**, **E**) In vitro cleavage of immunopurified CYLD, as in (**C**), incubated with the indicated recombinant human CASPs. In (**E**), cell lysates from THP-1 treated with ABT-737 and S63845 (A/S, 1 μM each) for 1 h were also analyzed. (**F**) Measured and expected molecular weights of CYLD and its cleaved fragment (*n* = 3; mean ± SEM; ns, not significant; ANOVA). *P* values for each comparison: Full-length, rhCYLD vs endogenous: *P* = 0.9274; Cleaved, rhCYLD vs endogenous: *P* > 0.9999. Rec., recombinant. (**G**) in vitro cleavage of immunopurified GSDMD, as in (**A**), incubated with the indicated CASPs. (**H**) Immunoblot analysis of control cells (CYLD^WT/WT^) or knock-in HL-60 cells engineered to express heterozygous D364A mutation (CYLD^WT/D364A^), treated with ABT-737 and S63845 (A/S) for 1 h. (**I**) in vitro cleavage of CYLD incubated with the indicated CASPs. (**J**) HEK293T cells were transfected with the indicated FLAG/HA-CYLD plasmids. After 24 h, cell lysates were prepared and immunoprecipitated with FLAG antibodies. Samples were eluted with FLAG peptides before incubation with CASP7 as indicated. Samples were analyzed by immunoblotting as indicated. (**K**) Immunoblot analysis of Ctrl^sg^, CASP3^sg^, CASP8^sg^, or BAX/BAK^sg^ MDA-MB-231 cells treated with ABT-737 and S63845 (A/S, 10 μM each) for 2 h. The presented immunoblots are representative of at least three independent experiments. Source data are available online for this figure.

CASP8 and MALT1 have previously been shown to cleave CYLD after the residues Asp215 and Arg324, respectively (Fig. 2A) (Staal et al, 2011; O’Donnell et al, 2011). However, the size of the fragments observed upon ABT-737 and S63845 treatment differs from what is expected from CASP8 proteolysis, suggesting a different cleavage site. Accordingly, CYLD processing normally occurred in MDA-MB-231 cells deficient for CASP8. However, CYLD remained intact and unprocessed in CASP3-deleted cells (Fig. 2B). Similar results were obtained with CASP3^sg^ THP-1 cells or with MCF7 cells, which naturally lack the expression of CASP3 (Jänicke, 2009) (Figs. 2C and EV3A). In keeping with this, the addition of cytochrome c and dATP to cell extracts from MCF7 did not cause cleavage of CYLD (Fig. 2D). As expected, CASP3 activation and subsequent CYLD proteolysis upon ABT-737 and S63845 treatment were suppressed in cells lacking BAX and BAK (Fig. 2B). In addition, we found that treating cells with the MALT1 inhibitor MLT-985 (Quancard et al, 2020) did not affect CYLD cleavage triggered by BH3 mimetics (Fig. EV3B). The adapter SPATA2 binds the COOH-terminal USP domain of CYLD and positively regulates extrinsic cell death (Fig. 2A) (Kupka et al, 2016; Elliott et al, 2016; Wagner et al, 2016; Schlicher et al, 2016). Accordingly, we observed a constitutive association of CYLD with SPATA2 (Fig. 2E). Upon treatment with ABT-737 and S63845, the CYLD COOH-terminal fragment and not the NH_2_-terminal fragment remained bound (Fig. 2E). Yet, targeting SPATA2 with sgRNAs had no overt effect on acute cell death triggered by ABT-737 and S63845 (Fig. 2F). This was accompanied by a minimal impact of SPATA2 on BH3 mimetics-mediated CYLD cleavage (Fig. 2G). Thus, CYLD is cleaved at a previously unrecognized site during mitochondrial apoptosis. This proteolysis relies on CASP3 independently of SPATA2.

### CASP3 processes CYLD at Asp364

**Figure 3:**
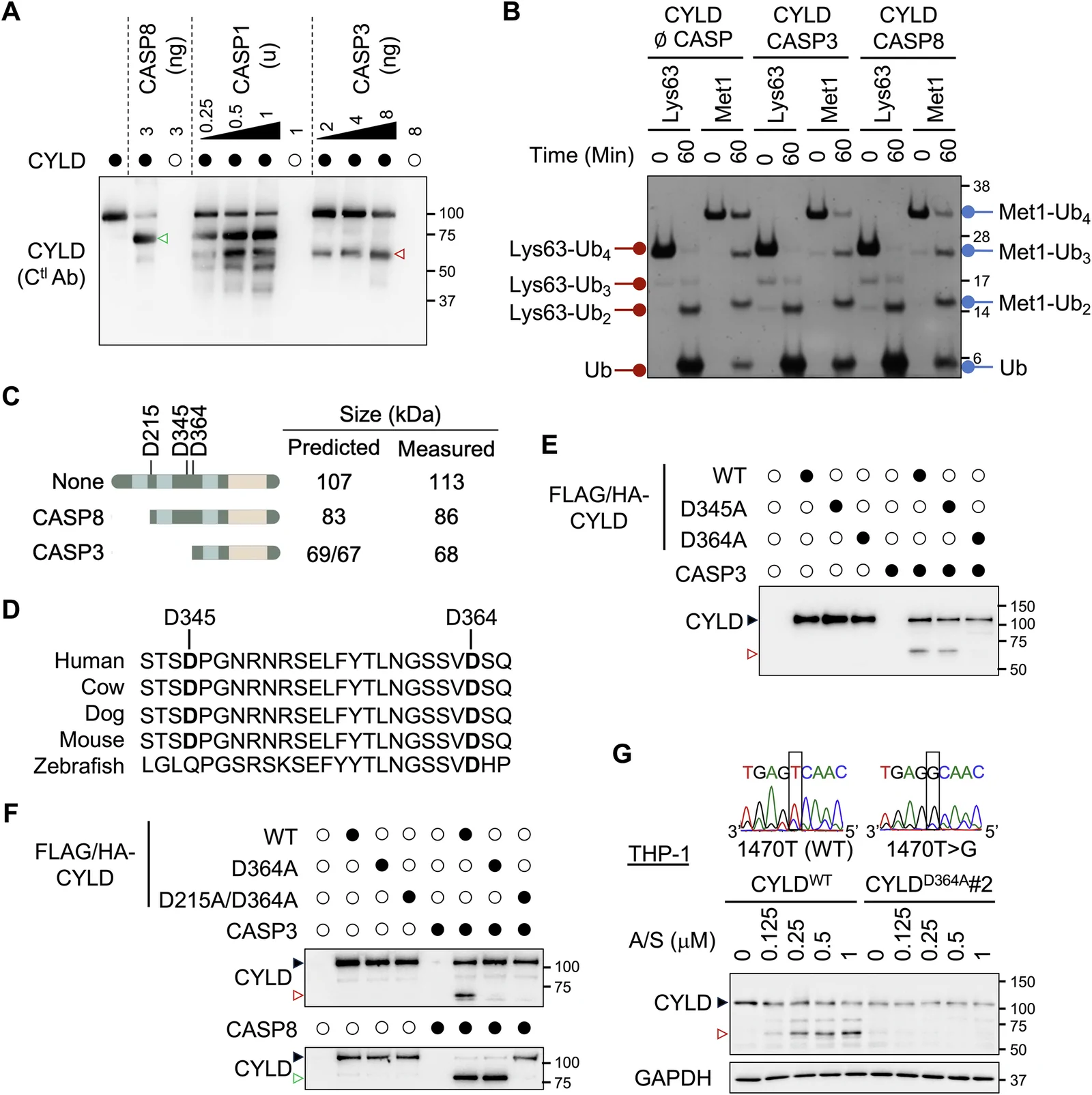
CYLD is cleaved by CASP3 at aspartic acid 364. (**A**) In vitro cleavage of recombinant human CYLD incubated with the indicated recombinant CASPs. Closed and open triangles indicate full-length and cleaved CYLD. (**B**) Purified CYLD was incubated together with the indicated CASPs, and CYLD DUB activity was assessed using Lys63-Ub_4_ and Met1-Ub_4_ as substrates. Samples were incubated for 1 h, resolved by SDS-PAGE, and stained using silver stain. (**C**) Schematic of CYLD protein indicating the predicted CASP3 cleavage sites (D345 and D364) in addition to the known CASP8 cleavage site. The predicted and measured COOH-terminal products are also shown. (**D**) Alignment of CYLD amino acid sequences from multiple species. The conserved aspartic acids are highlighted in bold. (**E**, **F**) HEK293T cells were transfected with the indicated FLAG/HA-CYLD plasmids. After 24 h, cell lysates were prepared and immunoprecipitated with FLAG antibodies. Samples were eluted with FLAG peptides before incubation with recombinant CASPs as indicated. Samples were analyzed by immunoblotting as indicated. (**G**) Immunoblots for indicated proteins in control cells (CYLD^WT^) or knock-in THP-1 cells engineered to express homozygous D364A mutation (CYLD^D364A^), treated with ABT-737 and S63845 (A/S) for 1 h. The presented immunoblots are representative of at least three independent experiments. Source data are available online for this figure.

To test whether CYLD might be a direct target of CASP3, we performed an in vitro cleavage assay by mixing recombinant human CYLD or immunopurified FLAG-HA-tagged human CYLD with active CASPs (Fig. EV3C). As expected (O’Donnell et al, 2011), CYLD was directly cleaved by CASP8, generating an approximately 86 kDa COOH-terminal fragment, consistent with cleavage at Asp215 (Figs. 3A and EV3D). Supporting our in cellulo data, CASP3 also cleaved CYLD at a specific site, distinct from the CASP8 site, generating a fragment that matched the one observed during mitochondrial apoptosis (Figs. 3A and EV3E,F). Of note, CASP1 cleaved CYLD in multiple fragments, phenocopying results obtained in pyroptotic samples (Figs. 3A and EV3D). This was not caused by a nonspecific activity of CASP1, as FLAG-tagged GSDMD, used as a control, was processed to generate a single fragment (Fig. EV3G). CASP1 displays concentration-dependent promiscuity toward various substrates (Walsh et al, 2011), and CYLD might be one of them. Next, we carried out in vitro DUB assays on Lys63- and Met1-linked tetra-ubiquitin chains with CYLD and CASPs (Elliott et al, 2021). This showed that when cleaved by CASP3 and CASP8, CYLD still efficiently cleaves Lys63- and Met1 linkages (Fig. 3B). These results suggest that, in addition to CASP8 and MALT1, CYLD is directly and differentially cleaved by CASP1 and CASP3.

We next conducted an in silico analysis of CYLD sequences from several species to identify putative CASP3 cleavage sites compatible with the observed fragments. This uncovered two conserved potential motifs, but only the mutation of Asp364 and not Asp345 prevented the cleavage of CYLD by recombinant CASP3 (Fig. 3C–E). Nonetheless, CYLD D364A was still efficiently cleaved by recombinant CASP8 in vitro unless the Asp215 residue was mutated (Fig. 3F). Next, we deployed a CRISPR/Cas9-based knock-in strategy to generate cells harboring a CYLD D364A mutation and obtained THP-1 cells with a homozygous CYLD D364A mutation (CYLD^D364A/D364A^). In these cells, the cleavage of CYLD upon ABT-737 and S63845 was significantly reduced (Fig. 3G). This was also true in HL-60 cells with a heterozygous CYLD D364A mutation (Fig. EV3H). Hence, CASP3 cleaves CYLD at Asp364 during mitochondrial apoptosis.

CASP3 and CASP7 are frequently considered as effector CASPs that share a similar repertoire of substrates (Thornberry et al, 1997). However, accumulating evidence indicates that they are functionally distinct proteases, with CASP3 acting as the major effector protease, whereas CASP7 exhibits a more selective set of substrates. In vitro, we observed that CASP7 cleaves CYLD, generating a main cleavage product similar to that produced by CASP3 (Fig. EV3I). This processing was abolished when the CYLD D364A mutant was used, indicating that both CASP3 and CASP7 cleave CYLD at the same site (Fig. EV3J). Furthermore, active CASP7 was detected in lysates from cells treated with BH3 mimetics, and its activation was only partly dependent on CASP3 (Fig. EV3K). Despite this, CYLD cleavage was prevented in CASP3 knockout cells (Fig. EV3K). Collectively, these findings suggest that CYLD is predominantly cleaved by CASP3 in cells undergoing mitochondrial apoptosis.

### CYLD cleavage tempers interferon signaling associated with mitochondrial apoptosis

**Figure EV4:**
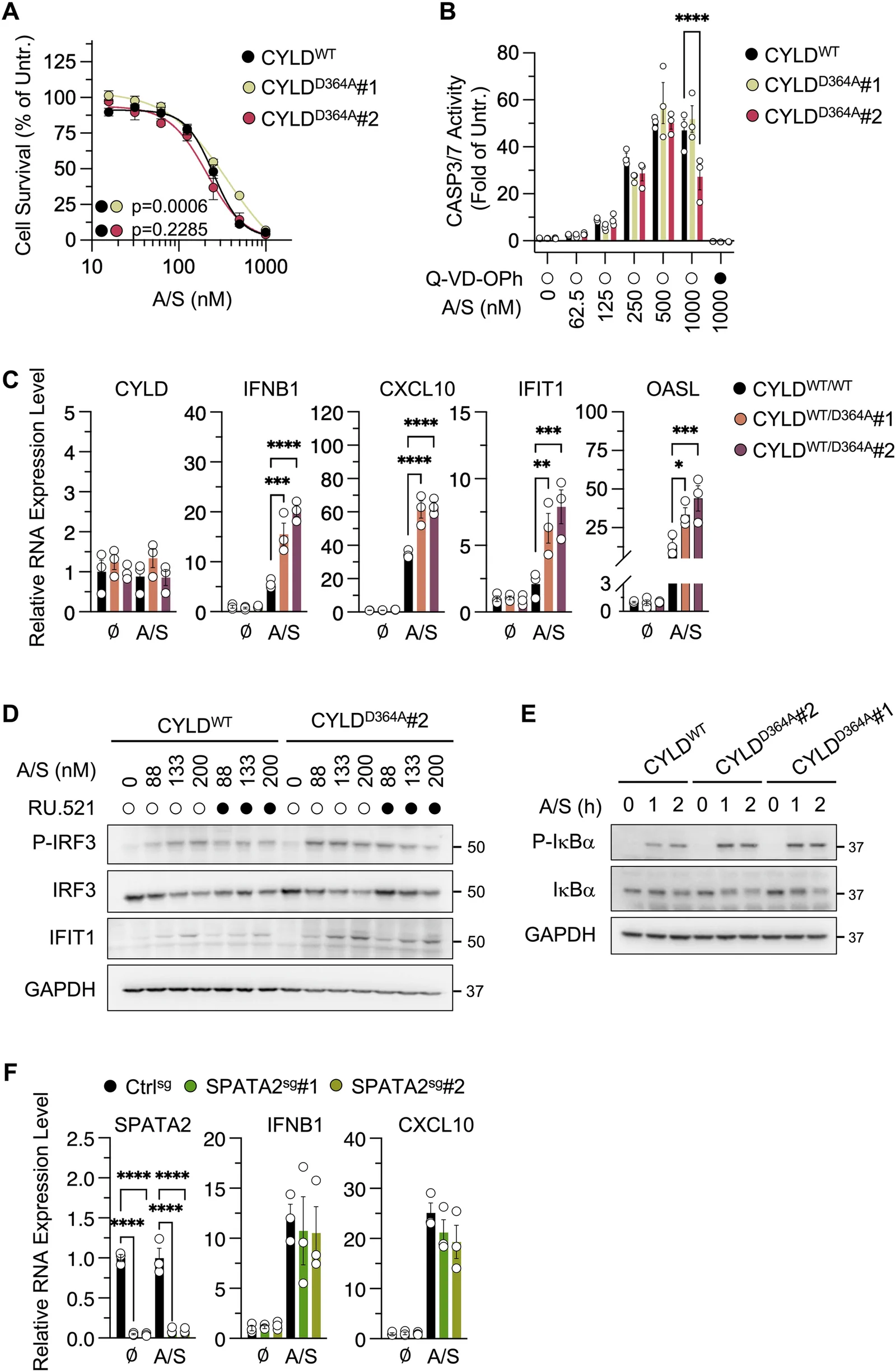
Cells expressing CASP3-resistant CYLD display enhanced interferon signaling. Related to Fig. 4. (**A**) Dose–response curves in the indicated THP-1 cell genotypes treated with escalating doses of ABT-737 and S63845 (A/S, 15.625, 31.25, 62.5, 125, 250, 500, and 1000 nM) for 1 h. Metabolic cell survival was assessed by CellTiter Glo (*n* = 4; mean ± SEM; extra sum-of-squares F test; *P* values for each comparison: CYLD^WT^ vs CYLD^D364A#1^: *P* = 0.0006, CYLD^WT^ vs CYLD^D364A#2^: *P* = 0.2285). (**B**) CASP3/7 Glo assay in the indicated THP-1 cell genotypes treated with escalating concentrations of A/S, as indicated. When indicated, cells were pretreated with Q-VD-OPh (10 μM, 30 min) (*n* = 3; mean ± SEM; two-way ANOVA; ****P < 0.0001; *P* values for CYLD^WT^ vs CYLD^D364A#2^, A/S 1000 nM: *P* < 0.0001). (**C**) qPCR analysis of the indicated genes in the indicated HL-60 cell genotypes treated with ABT-737 and S63845 (A/S, 1 μM each) for 2 h (*n* = 3; mean ± SEM; *P < 0.05, **P < 0.01, ***P < 0.001, ****P < 0.0001; one-way ANOVA). *P* values for each comparison (A/S treatment): INFB1, CYLD^WT^ vs CYLD^D364A#1^: *P* = 0.0001; INFB1, CYLD^WT^ vs CYLD^D364A#2^: *P* < 0.0001; CXCL10, CYLD^WT^ vs CYLD^D364A#1^: *P* < 0.0001; CXCL10, CYLD^WT^ vs CYLD^D364A#2^: *P* < 0.0001; OASL, CYLD^WT^ vs CYLD^D364A#1^: *P* = 0.0218; OASL, CYLD^WT^ vs CYLD^D364A#2^: *P* = 0.0010; IFIT1, CYLD^WT^ vs CYLD^D364A#1^: *P* = 0.0071; IFIT1, CYLD^WT^ vs CYLD^D364A#2^: *P* = 0.0005. (**D**) Immunoblot analysis of THP-1 cell genotypes pretreated or not with the cGAS inhibitor RU.521 (10 μM, 30 min) and stimulated with A/S as indicated for 24 h. Numbers to the right indicate molecular mass markers. (**E**) Immunoblot analysis of the indicated THP-1 cell genotypes exposed to A/S (1 μM each) for 1 and 2 h. (**F**) qPCR analysis of SPATA2, IFNB1, and CXCL10 in the indicated THP-1 cell genotypes treated with ABT-737 and S63845 (A/S, 1 μM each) for 2 h (*n* = 3; mean ± SEM; ****P < 0.0001; one-way ANOVA). *P* values for each comparison: SPATA2, Untreated Ctrl^sg^ vs SPATA2^sg#1^: *P *< 0.0001; SPATA2, Untreated SPATA2, Ctrl^sg^ vs SPATA2^sg#2^: *P* < 0.0001; SPATA2, A/S Ctrl^sg^ vs SPATA2^sg#1^: *P* < 0.0001; SPATA2, A/S Ctrl^sg^ vs SPATA2^sg#2^: *P* < 0.0001. The presented immunoblots are representative of at least three independent experiments. Source data are available online for this figure.

**Figure 4:**
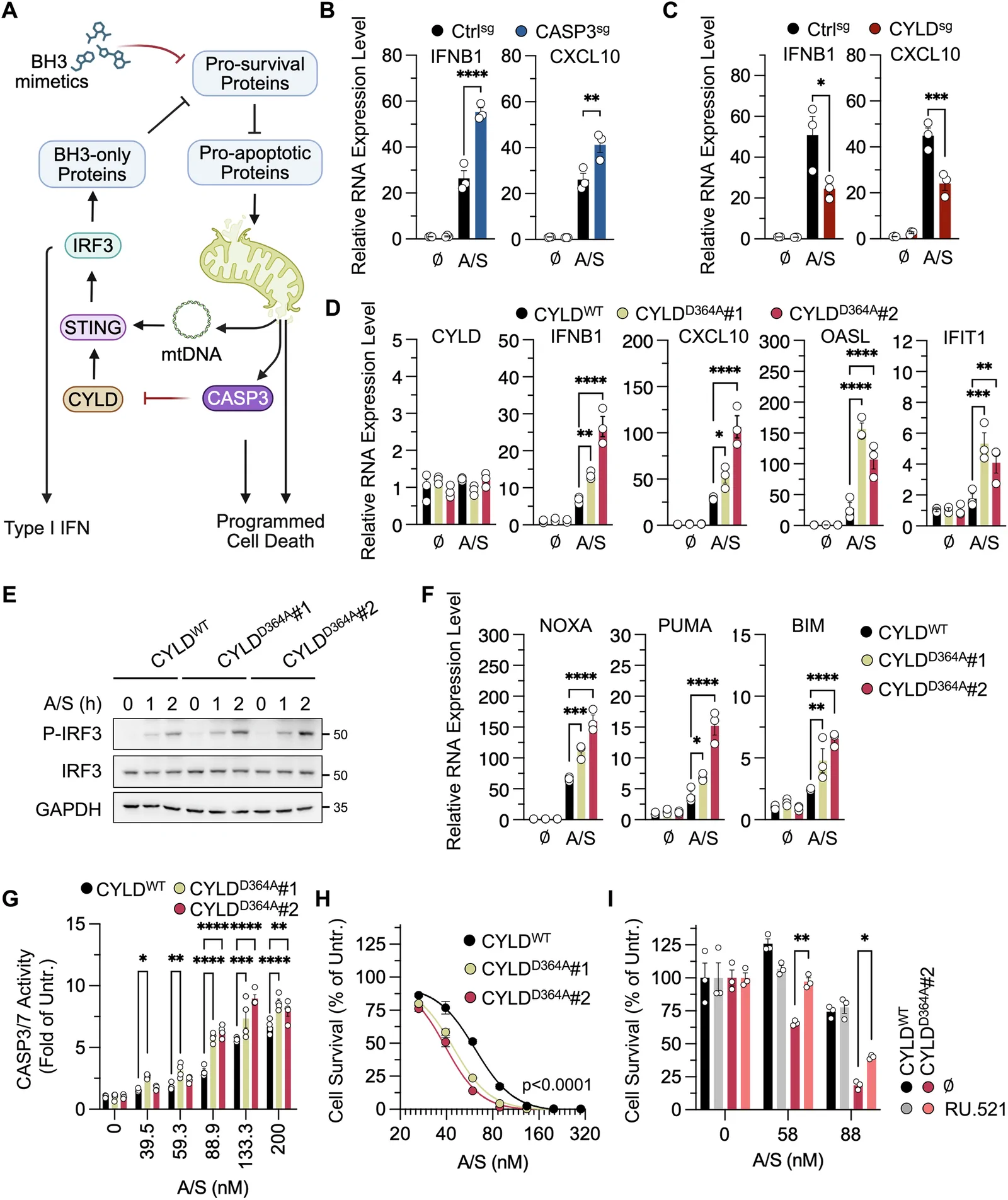
Interferon signaling and cell death during mitochondrial apoptosis are enhanced when CASP3-mediated CYLD cleavage is impaired. (**A**) Model depicting the intertwined regulation of cell death and inflammation during mitochondrial apoptosis. (**B**–**D**) RT-qPCR analysis of the indicated genes in the indicated THP-1 cell genotypes treated with ABT-737 and S63845 (A/S, 1 μM each) for 2 h (*n* = 3; mean ± SEM; *P < 0.05, **P < 0.01, ***P < 0.001, ****P < 0.0001; one-way ANOVA). *P* values for each comparison (A/S treatment): INFB1, Ctrl^sg^ vs CASP3^sg^: *P* = 0.000028; CXCL10, Ctrl^sg^ vs CASP3^sg^: *P* = 0.0048; INFB1, Ctrl^sg^ vs CYLD^sg^: *P* = 0.0185; CXCL10, Ctrl^sg^ vs CYLD^sg^: *P* = 0.0008; INFB1, CYLD^WT^ vs CYLD^D364A#1^: *P* = 0.006939; INFB1, CYLD^WT^ vs CYLD^D364A#2^: *P* < 0.0001; CXCL10, CYLD^WT^ vs CYLD^D364A#1^: *P* = 0.0457; CXCL10, CYLD^WT^ vs CYLD^D364A#2^: *P* < 0.0001; OASL, CYLD^WT^ vs CYLD^D364A#1^: *P* < 0.0001; OASL, CYLD^WT^ vs CYLD^D364A#2^: *P* < 0.0001; IFIT1, CYLD^WT^ vs CYLD^D364A#1^: *P* = 0.0003; IFIT1, CYLD^WT^ vs CYLD^D364A#2^: *P* = 0.0094. (**E**) Immunoblot analysis of the indicated THP-1 cells treated with A/S (1 μM each) for 1 and 2 h. (**F**) RT-qPCR analysis of NOXA, PUMA, and BIM genes in the indicated THP-1 cell genotypes treated as in (**B**) (*n* = 3; mean ± SEM; *P < 0.05, **P < 0.01, ***P < 0.001, ****P < 0.0001; one-way ANOVA). *P* values for each comparison (A/S treatment): NOXA, CYLD^WT^ vs CYLD^D364A#1^: *P* = 0.0002; NOXA, CYLD^WT^ vs CYLD^D364A#2^: *P* < 0.0001; PUMA, CYLD^WT^ vs CYLD^D364A#1^: *P* = 0.0458; PUMA, CYLD^WT^ vs CYLD^D364A#2^: *P* < 0.0001; BIM, CYLD^WT^ vs CYLD^D364A#1^: *P* = 0.0079; BIM, CYLD^WT^ vs CYLD^D364A#2^: *P* < 0.0001. (**G**) CASP3/7 catalytic activity was measured by luminescent substrate cleavage (CASP Glo assay) in the indicated THP-1 genotypes treated with escalating concentrations of A/S, as indicated (*n* = 4; mean ± SEM; *P < 0.05, **P < 0.01, ***P < 0.001, ****P < 0.0001; two-way ANOVA). *P* values for each comparison: 39.5 nM, CYLD^WT^ vs CYLD^D364A#1^: *P* = 0.0176; 59.3 nM, CYLD^WT^ vs CYLD^D364A#1^: *P* = 0.0051; 88.9 nM, CYLD^WT^ vs CYLD^D364A#1^: *P* < 0.0001; 88.9 nM, CYLD^WT^ vs CYLD^D364A#2^: *P* < 0.0001; 133.3 nM, CYLD^WT^ vs CYLD^D364A#1^: *P* = 0.0002; 133.3 nM, CYLD^WT^ vs CYLD^D364A#2^: *P* < 0.0001; 200 nM, CYLD^WT^ vs CYLD^D364A#1^: *P* < 0.0001; 200 nM, CYLD^WT^ vs CYLD^D364A#2^: *P *= 0070. (**H**) Dose–response curves of the indicated THP-1 cells treated with escalating doses of ABT-737 and S63845 (A/S, 26.3, 39.5, 59, 89, 133, 200, and 300 nM) for 24 h and analyzed in a CellTiter Glo assay (*n* = 4; extra sum-of-squares F test; *P* values for each comparison: CYLD^WT^ vs CYLD^D364A#1^: *P* < 0.0001; CYLD^WT^ vs CYLD^D364A#2^: *P* < 0.0001). (**I**) CellTiter Glo assay of cells in the indicated THP-1 cell genotypes pretreated or not with RU.521 (5 μM, 30 min) and treated with A/S as indicated for 24 h (*n* = 3; mean ± SEM; *P < 0.05, **P < 0.01; two-way ANOVA). *P* values for each comparison: 58 nM, CYLD CYLD^D364A#2^ untreated vs CYLD^D364A#2^ RU.521: *P* = 0.0017; 88 nM, CYLD CYLD^D364A#2^ untreated vs CYLD^D364A#2^ RU.521: *P* = 0.0373. The presented immunoblots are representative of at least three independent experiments. Source data are available online for this figure.

We next assessed the impact of CYLD cleavage by CASP3 during acute cell death triggered by the combination of ABT-737 and S63845 during 1 h and found that THP-1 expressing a CASP3-resistant CYLD did not display an overt change in cell death and CASP3/7 activation (Fig. EV4A,B). In addition to cell death, MOMP triggers the release of mtDNA into the cytosol through BAX and BAK pores, activating IRF3 transcription factor and the subsequent production of type I IFN via the cGAS-STING pathway (Rongvaux et al, 2014; White et al, 2014; Riley et al, 2018) (Fig. 4A). MOMP-mediated activation of CASP3 counteracts IRF3 signaling by cleaving and inactivating key regulators of the pathway, including cGAS and IRF3 (Ning et al, 2019). Given that CYLD positively regulates the cGAS-STING pathway (Zhang et al, 2018, 2025), we hypothesized that cleavage by CASP3 antagonizes CYLD’s function. We first confirmed by RT-qPCR that the induction of IFNB1 and the downstream IFN-stimulated gene (ISG) CXCL10 were increased in CASP3^sg^ cells and decreased in CYLD^sg^ cells treated with ABT-737 and S63845 (Fig. 4B,C). In these conditions, THP-1 and HL-60 cells expressing CASP3-resistant CYLD displayed enhanced mRNA production of IFNB1 and the ISGs CXCL10, IFIT1, and OASL (Figs. 4D and EV4C). Accordingly, the phosphorylation of IRF3 and the expression of the IFN-stimulated gene IFIT1 were increased in THP-1 cells expressing CYLD D364A (Figs. 4E and EV4D). As expected (Rongvaux et al, 2014; White et al, 2014; Riley et al, 2018), the pharmacological inhibition of cGAS with RU.521 attenuated IRF3 activation (Fig. EV4D). Consistent with previous findings (Vringer et al, 2024), MOMP also triggered NF-κB activation in THP-1 cells, which was further increased in cells expressing CYLD D364A (Fig. EV4E). Of note, no overt changes in the induction of IFNB1 and ISG mRNAs were observed in SPATA2^sg^ cells (Fig. EV4F). Altogether, these results suggest that CASP3 cleaves CYLD to restrict interferon signaling associated with cell death.

**Figure EV5:**
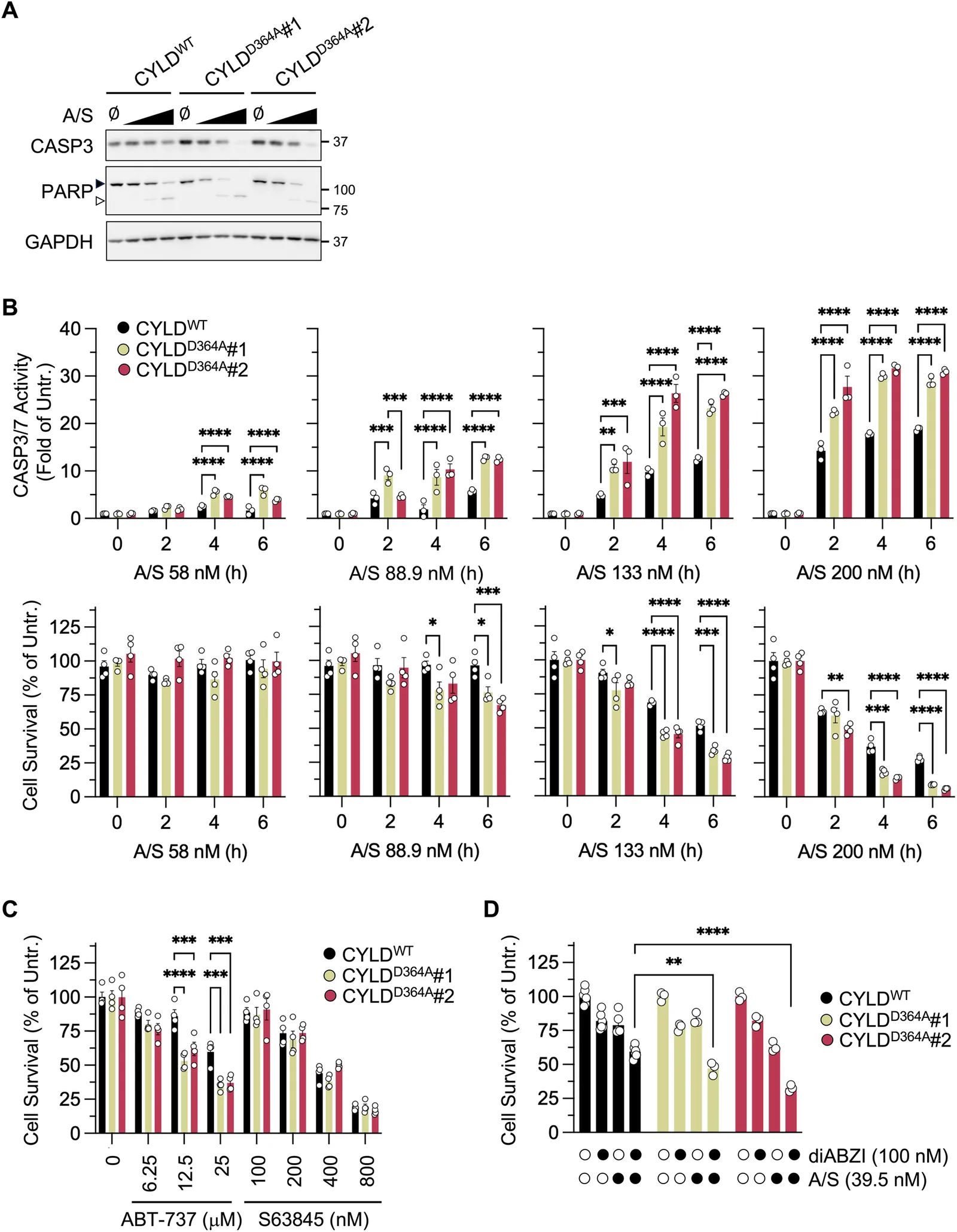
Cells expressing CASP3-resistant CYLD display enhanced cell death. Related to Fig. 4. (**A**) Immunoblot analysis of the indicated THP-1 cell genotypes treated with ABT-737 and S63845 (A/S, 39.5, 59, 89 nM each) for 24 h. Closed and open triangles indicate full-length and cleaved PARP. (**B**) CASP3/7 Glo and CellTiter Glo assays of cells in the indicated THP-1 cell genotypes treated for 0, 2, 4, and 6 h with the indicated doses of ABT-737 and S63845 (A/S) (*n* = 3 for CellTiter Glo and *n* = 4 for CASP3/7 Glo; mean ± SEM; *P < 0.05, **P < 0.01, ***P < 0.001, ****P < 0.0001; two-way ANOVA). For CASP3/7 Glo, *P* values for each comparison: 58 nM 4 h, CYLD^WT^ vs CYLD^D364A#1^: *P* < 0.0001; 58 nM 4 h, CYLD^WT^ vs CYLD^D364A#2^: *P* < 0.0001; 58 nM 6 h, CYLD^WT^ vs CYLD^D364A#1^: *P* < 0.0001; 58 nM 6 h, CYLD^WT^ vs CYLD^D364A#2^: *P* < 0.0001; 88.9 nM 2 h, CYLD^WT^ vs CYLD^D364A#1^: *P* = 0.0003; 88.9 nM 2 h, CYLD^WT^ vs CYLD^D364A#2^: *P* = 0.9257; 88.9 nM 4 h, CYLD^WT^ vs CYLD^D364A#1^: *P* < 0.0001; 88.9 nM 4 h, CYLD^WT^ vs CYLD^D364A#2^: *P* < 0.0001; 88.9 nM 6 h, CYLD^WT^ vs CYLD^D364A#1^: *P* < 0.0001; 88.9 nM 6 h, CYLD^WT^ vs CYLD^D364A#2^: *P* < 0.0001; 133 nM 2 h, CYLD^WT^ vs CYLD^D364A#1^: *P* = 0.0027; 133 nM 2 h, CYLD^WT^ vs CYLD^D364A#2^: *P* = 0.0004; 133 nM 4 h, CYLD^WT^ vs CYLD^D364A#1^: *P* < 0.0001; 133 nM 4 h, CYLD^WT^ vs CYLD^D364A#2^: *P* < 0.0001; 133 nM 6 h, CYLD^WT^ vs CYLD^D364A#1^: *P* < 0.0001; 133 nM 6 h, CYLD^WT^ vs CYLD^D364A#2^: *P* < 0.0001; 200 nM 2 h, CYLD^WT^ vs CYLD^D364A#1^: *P* < 0.0001; 200 nM 2 h, CYLD^WT^ vs CYLD^D364A#2^: *P* < 0.0001; 200 nM 4 h, CYLD^WT^ vs CYLD^D364A#1^: *P* < 0.0001; 200 nM 4 h, CYLD^WT^ vs CYLD^D364A#2^: *P* < 0.0001; 200 nM 6 h, CYLD^WT^ vs CYLD^D364A#1^: *P* < 0.0001; 200 nM 6 h, CYLD^WT^ vs CYLD^D364A#2^: *P* < 0.0001. For CellTiter Glo, *P* values for each comparison: 88.9 nM 4 h, CYLD^WT^ vs CYLD^D364A#1^: *P* = 0.0242; 88.9 nM 4 h, CYLD^WT^ vs CYLD^D364A#2^: *P* = 0.1208; 88.9 nM 6 h, CYLD^WT^ vs CYLD^D364A#1^: *P* = 0.0164; 88.9 nM 6 h, CYLD^WT^ vs CYLD^D364A#2^: *P* = 0.0004; 133 nM 2 h, CYLD^WT^ vs CYLD^D364A#1^: *P* = 0.0131; 133 nM 2 h, CYLD^WT^ vs CYLD^D364A#2^: *P* = 0.2136; 133 nM 4 h, CYLD^WT^ vs CYLD^D364A#1^: *P* < 0.0001; 133 nM 4 h, CYLD^WT^ vs CYLD^D364A#2^: *P *< 0.0001; 133 nM 6 h, CYLD^WT^ vs CYLD^D364A#1^: *P* = 0.0004; 133 nM 6 h, CYLD^WT^ vs CYLD^D364A#2^: *P* < 0.0001; 200 nM 2 h, CYLD^WT^ vs CYLD^D364A#1^: *P* = 0.7666; 200 nM 2 h, CYLD^WT^ vs CYLD^D364A#2^: *P* = 0.0057; 200 nM 4 h, CYLD^WT^ vs CYLD^D364A#1^: *P* = 0.0001; 200 nM 4 h, CYLD^WT^ vs CYLD^D364A#2^: *P* < 0.0001; 200 nM 6 h, CYLD^WT^ vs CYLD^D364A#1^: *P* < 0.0001; 200 nM 6 h, CYLD^WT^ vs CYLD^D364A#2^: *P* < 0.0001. (**C**) Metabolic cell survival measured by CellTiter Glo in the indicated THP-1 cell genotypes treated with escalating doses of ABT-737 or S63845 for 24 h (*n* = 3; mean ± SEM; ***P < 0.001, ****P < 0.0001; two-way ANOVA). *P* values for each comparison: ABT-737 12.5 μM, CYLD^WT^ vs CYLD^D364A#1^: *P* < 0.0001; ABT-737 12.5 μM, CYLD^WT^ vs CYLD^D364A#2^: *P* = 0.0002; ABT-737 25 μM, CYLD^WT^ vs CYLD^D364A#1^: *P* = 0.0002; ABT-737 25 μM, CYLD^WT^ vs CYLD^D364A#2^: *P* = 0.0004. (**D**) Cells as in (**A**) treated with A/S (39.5 nM each) in combination with diABZI (100 nM) for 24 h (*n* = 3 or 6; mean ± SEM; **P < 0.01, ****P < 0.0001; two-way ANOVA). *P* values for each comparison: CYLD^WT^ vs CYLD^D364A#1^: *P* = 0.0051; CYLD^WT^ vs CYLD^D364A#2^: *P* < 0.0001. The presented immunoblots are representative of at least three independent experiments. Source data are available online for this figure.

IRF3 has been shown to induce the transcription of proapoptotic members of the BCL-2 family and further amplify mitochondrial apoptosis (Fig. 4A) (Gulen et al, 2017; Diepstraten et al, 2024). Consistent with increased IRF3 activation, THP-1 cells expressing CASP3-resistant CYLD treated with ABT-737 and S63845 showed upregulation of the BH3-only proapoptotic BCL-2 family members NOXA, PUMA, and BIM (Fig. 4F). Thus, we reasoned that cell death may be enhanced in cells expressing CASP3-resistant CYLD when exposed to BH3 mimetics used at lower concentrations to induce milder and slower cell death. Accordingly, we observed increased CASP3/7 activity and cell death in THP-1 cells expressing a CASP3-resistant version of CYLD treated with escalating doses of ABT-737 and S63845 at different time-points (Figs. 4G,H and EV5A,B). Supporting previous works (Greaves et al, 2019), THP-1 cells were more sensitive to MCL1 inhibition with S63845 than BCL-2/BCL-XL/BCL-w inhibition with ABT-737. Nevertheless, we found that cells expressing CYLD D364A displayed enhanced ABT-737- and not S63845-mediated cell death (Fig. EV5C). Of note, the inhibition of cGAS with RU.521 partly normalized the enhanced cell death observed in THP-1 expressing CASP3-resistant CYLD (Fig. 4I). Conversely, cell death was further increased in cells expressing CYLD D364A when treated with sublethal doses of BH3 mimetic and the STING agonist diABZI (Fig. EV5D). Thus, CYLD cleavage attenuates apoptosis.

In conclusion, our findings position CYLD proteolysis by CASP3 as a critical checkpoint in balancing inflammation and cell death. While this cleavage is dispensable for acute mitochondrial cell death, it safeguards cells from launching interferon-mediated inflammation and cell death programs. Thus, manipulating CYLD proteolysis by CASP3 will provide a window to modulate the inflammatory nature of cell death.

We report that CYLD cleavage by CASP3 uncouples the first two CAP-Gly domains of CYLD from a COOH-terminal fragment that includes a phospho-rich patch, the third CAP-Gly3 motif, and the catalytic domain. These fragments dissociate during mitochondrial apoptosis, with the COOH part remaining associated with SPATA2. Since CAP-Gly1 and CAP-Gly2 bind to microtubules (Gao et al, 2008; Wickström et al, 2010), we expect processing to jeopardize CYLD partitioning between its known localization at the cytosol, the centrosome, and centriolar satellites. Hence, although we found that the COOH-terminal fragment retains the minimal domain previously shown to allow optimal enzyme activity (Elliott et al, 2021) and efficiently removes Lys63- and Met1-linked chains in vitro, cleavage may rewire the interactome of CYLD and substrate specificity. CASP8, which detaches the CAP-Gly1 (O’Donnell et al, 2011), and CASP1, which cleaves at several sites, may differentially affect CYLD fate. Notably, MALT1 cleaves CYLD after the residue Arg324, a site located only 40 amino acids upstream of the CASP3 site (Staal et al, 2011). However, the consequences of cleavage by MALT1 are not fully understood (Staal et al, 2011; Gewies et al, 2014). Given that antigen receptor ligation both activates MALT1 and enhances IRF3 signaling driven by a STING agonist (Kuhl et al, 2023), we propose that MALT1-mediated CYLD cleavage may serve to fine-tune IRF3 activation in lymphocytes.

We also provide evidence that CYLD is cleaved by CASP1 during pyroptosis at multiple sites distinct from those targeted by CASP3, CASP8, or MALT1. These observations suggest that site-specific cleavage events tailor CYLD functions to the cellular context. Additional CASPs, particularly CASP7, could potentially contribute to CYLD cleavage at D364, given their overlapping substrate specificities (Thornberry et al, 1997). Accordingly, CASP7 efficiently cleaves CYLD in vitro, generating a product similar to that produced by CASP3. Nevertheless, during mitochondrial apoptosis, CYLD proteolysis is predominantly mediated by CASP3 despite CASP7 activation, consistent with the idea that CASP7 accesses a more restricted set of substrates than CASP3 (Walsh et al, 2008; Slee et al, 1999, 2001).

Whether CASPs can act in concert or if cleavage site selection can shift under different conditions remains an open question. Although CASP3 preferentially cleaves CYLD at Asp364, we also detected a less prominent cleavage at Asp215, even in CASP8-deficient cells. Moreover, thymocytes from mice expressing CYLD D215A still exhibit CYLD cleavage upon Fas ligand stimulation to activate CASP8, with a switch to fragments consistent with cleavage at Asp364 (Newton et al, 2019). Whether this site switch involves CASP3 and/or CASP8 is unknown. CASP activation often involves the assembly of dedicated supramolecular complexes, raising the possibility that spatial compartmentalization dictates the differential cleavage of CYLD. For instance, CYLD is dynamically recruited to the TNF receptor signaling complexes in proximity to CASP8 following TNFα stimulation (Draber et al, 2015; O’Donnell et al, 2011; Micheau and Tschopp, 2003). Additional work is therefore required to elucidate whether CYLD associates with CASP3-containing signaling platform during mitochondrial apoptosis. Intriguingly, a pool of CYLD localizes to centriolar satellites, and we have previously shown that several components of these proteinaceous structures are cleaved by CASP3 downstream of MOMP (Douanne et al, 2019; Renaud et al, 2023), suggesting a potential spatial node for triggering CASP3-dependent proteolysis.

Together, our findings point to a previously unrecognized “proteolytic code”, where distinct cleavage events selectively modulate protein function, which may extend beyond CYLD. Deciphering this code could enable the selective reprogramming of protein functions without globally compromising protein activity.

## Methods

**Table:** Reagents and tools table

Reagent/resource	Reference or source	Identifier or catalog number
**Experimental models**		
THP-1 (*H. sapiens*)	ATCC	TIB-202
HEK293T (*H. sapiens*)	ATCC	CRL-3216
MCF7 (*H. sapiens*)	ATCC	HTB-22
HL-60 (*H. sapiens*)	Provided by Pr J. Gaschet, CRCI^2^NA, France	
MOLM-13 (*H. sapiens*)	Provided by Dr. C Woo, Harvard University, USA	
MOLM-14 (*H. sapiens*)	Provided by Pr. J. Gaschet, CRCI^2^NA, France	
MDA-MB-231 (*H. sapiens*)	Provided by Dr. S. Barillé-Nion, CRCI^2^NA, France	
MDA-MB-231 CASP3^sg^	Provided by Dr. S. Barillé-Nion, CRCI^2^NA, France	
MDA-MB-231 CASP8^sg^	Provided by Dr. S. Barillé-Nion, CRCI^2^NA, France	
MDA-MB-231 BAX/BAK^sg^	Provided by Dr. S. Barillé-Nion, CRCI^2^NA, France	
THP-1 CASP3^sg^	This study	
THP-1 CYLD^sg^	This study	
THP-1 SPATA2^sg^	This study	
THP-1 CYLD^D364A^	This study	
HL-60^WT/D364A^	This study	
**Plasmids**		
MSCV-TAP(FLAG-HA)CYLD	Addgene	60031
MSCV-TAP(FLAG-HA)CYLD D215A	This study	
MSCV-TAP(FLAG-HA)CYLD D345A	This study	
MSCV-TAP(FLAG-HA)CYLD D364A	This study	
MSCV-TAP(FLAG-HA)CYLD D215A/D364A	This study	
FLAG-GSDMD	Addgene	80950
VSVg	Addgene	8454
PAX2	Addgene	12260
**Antibodies**		
CYLD	Santa Cruz Biotechnology	sc-137139
PARP	Santa Cruz Biotechnology	sc-8007
GAPDH	Santa Cruz Biotechnology	sc-32233
SPATA2	Santa Cruz Biotechnology	sc-51583
HMGB1	Santa Cruz Biotechnology	sc-56698
Ubiquitin	Santa Cruz Biotechnology	sc-8017
BAK	Cell Signaling Technologies	12105
CASP3	Cell Signaling Technologies	9662
Cleaved CASP7	Cell Signaling Technologies	8438
CASP8	Cell Signaling Technologies	9746
Cleaved CASP1	Cell Signaling Technologies	4199
IRF3	Cell Signaling Technologies	4302
p62	Cell Signaling Technologies	88588
P-IκBα	Cell Signaling Technologies	9246
IκBα	Cell Signaling Technologies	9242
IFIT1	Proteintech	23247-1-AP
α-Tubulin	Proteintech	66031-1-1g
BAX	Proteintech	50599-2
IRF3	Proteintech	11312-1-AP
CYLD	Invitrogen	43-7700
SPATA2	Bethyl Laboratories	A302-493A
P-IRF3 Ser386	Abcam	Ab76493
GSDMD	Atlas Laboratories	HPA044487
**Oligonucleotides and other sequence-based reagents**		
CYLD sgRNA	AGAATATTCAAGAATTCCTC	
CASP1 sgRNA	AAAGCTGTTTATCCGTTCCA	
GSDMD sgRNA	CTTCCACTTCTACGATGCCA	
CASP3 sgRNA	ATGTCGATGCAGCAAACCTC	TrueGuide Thermofisher
SPATA2 sgRNA	GATCAGCCGGAATCGATAAA	TrueGuide Thermofisher
Negative sgRNA sequence	Thermofisher	A35526
BAX sgRNA	AGTAGAAAAGGGCGACAACC	
BAK sgRNA	GCCATGCTGGTAGACGTGTA	
CASP3 sgRNA	ATGTCGATGCAGCAAACCTC	
CASP8 sgRNA	CACCGGCCTGGACTACATTCCGCAA	
ACTB primer forward	GGACTTCGAGCAAGAGATGG	
ACTB primer reverse	AGCACTGTGTTGGCGTACAG	
BIM primer forward	TAAGTTCTGAGTGTGACCGAGA	
BIM primer reverse	GCTCTGTCTGTAGGGAGGTAGG	
CXCL10 primer forward	GAAAGCAGTTAGCAAGGAAAGGTG	
CXCL10 primer reverse	ATGTAGGGAAGTGATGGGAGAGG	
CYLD primer forward	AAGGCTACAGGATCTACCTCAG	
CYLD primer reverse	GTGGTTGTGAGTCAACAGAAGA	
HPRT1 primer forward	TGACACTGGCAAAACAATGCA	
HPRT1 primer reverse	GGTCCTTTTCACCAGCAAGCT	
IFIT1 primer	GCGCTGGGTATGCGATCTC	
IFIT1 primer reverse	CAGCCTGCCTTAGGGGAAG	
IFNB1 primer forward	GCTTGGATTCCTACAAAGAAGCA	
IFNB1 primer reverse	ATAGATGGTCAATGCGGCGTC	
NOXA primer forward	ACCAAGCCGGATTTGCGATT	
NOXA primer reverse	ACTTGCACTTGTTCCTCGTGG	
OASL primer forward	CTGATGCAGGAACTGTATAGCAC	
OASL primer reverse	CACAGCGTCTAGCACCTCTT	
PUMA primer forward	GACCTCAACGCACAGTACGAG	
PUMA primer reverse	AGGAGTCCCATGATGAGATTGT	
SPATA2 primer forward	CGGCCAAGGACTACTACAAGC	
SPATA2 primer reverse	GGATGGGCGGATGATCTCA	
**Chemicals, enzymes, and other reagents**		
ABT-737	Selleckchem	S1002
Bafilomycin A1	Selleckchem	S1413
S63845	Selleckchem	S8383
Nigericin	Invivogen	tlrl-nig
LPS	Invivogen	tlrl-peklps
MCC950	Invivogen	inh-mcc
Recombinant human CASP3	Biotechne	707-C3-010/CF
Recombinant human CASP7	Biotechne	823-C7/CF
Recombinant human CASP8	Biotechne	705-C8-010/CF
Q-VD-O-Ph	Biotechne	OPH001
Cytochrome c	Merck/Sigma	C3131
dATP	Merck/Sigma	11934511001
Etoposide	Merck/Sigma	E1383
Staurosporine	Merck/Sigma	S4400
Raptinal	Merck/Sigma	SML1745
FLAG M2 Affinity Resin	Merck/Sigma	A2220
3X-FLAG Peptide	Merck/Sigma	F4799
MLT-985	MedChemExpress	HY-142648
RU.521	MedChemExpress	HY-112693
MG132	Cell Signaling Technologies	2194
Recombinant human CASP1	Abcam	ab39901
Polybrene	Santa Cruz Biotechnology	sc-134220
TrueGuide Cas9	Thermofisher	A36499
Neon Transfection kit	Thermofisher	MPK5000 MPK1096
Annexin V Alexa Fluor 594	Thermofisher	A13203
CellTiter Glo	Promega	G9243
CASP3/7 Glo	Promega	G8092
BCA kit	Interchim	UP40840A
Recombinant CYLD	Provided by P Elliott, Oxford University, UK	
Lys63-Ub4	Provided by P Elliott, Oxford University, UK	
Met1-Ub4	Provided by P Elliott, Oxford University, UK	
**Software**		
Prism 10	GraphPad	
FIDJI ImageJ	Version 2.16.0	
**Other**		
Incucyte S3	Sartorius	
FACS Aria III	BD	

### Cell culture and reagents

THP-1, HEK293T, and MCF7 were purchased from the American Type Culture Collection. THP-1 cells stably expressing HMGB1-Lucia were obtained from Invivogen. HL-60, OCI-AML5, and MOLM-14 were provided by J. Gaschet (CRCI^2^NA, France). MOLM-13 cells and MDA-MB-231 were a gift from C. Woo (Harvard University, USA) and S. Barillé-Nion (CRCI^2^NA, France), respectively. AML cell lines were cultured in RPMI 1640 supplemented with 20% FBS, 1% Glutamax, 100 mg/mL penicillin, 100 mg/mL streptomycin, 1% non-essential amino acids, 1 μM Hepes, and 100 μM sodium pyruvate. HEK293T, MCF7, and MDA-MB-231 cells were cultured in Dulbecco’s modified Eagle’s medium DMEM supplemented with 10% FBS, 1% Glutamax, 100 mg/mL penicillin, and 100 mg/mL streptomycin. Cells were maintained at 37 °C in a humidified incubator with 5% CO_2_, and were frequently tested for mycoplasma contamination.

ABT-737 (S1002), Bafilomycin A1 (S1413), and S63845 (S8383) were purchased from Selleckchem. LPS (tlrl-peklps), Nigericin (tlrl-nig), and MCC950 (inh-mcc) were obtained from Invivogen. Recombinant human CYLD (E-556), recombinant human CASP3 (707-C3-010/CF), recombinant human CASP7 (823-C7/CF), recombinant human CASP8 (705-C8-010/CF), and Q-VD-OPh (OPH001) were from Biotechne. Cytochrome c (C3131), dATP (11934511001), Etoposide (E1383), Staurosporine (S4400), and Raptinal (SML1745) were from Merck. MLT-985 (HY-142648) and RU.521 (HY-112693) were from MedChemExpress. MG132 (Cell Signaling Technology, 2194) and Recombinant human CASP1 (Abcam, ab39901) were also used.

### CRISPR/Cas9 knockout, knock-in, and site-directed mutagenesis

For CRISPR/Cas9 knockout (KO) editing in THP-1 cells, the following single-guide (sg) RNA sequences were used, targeting CYLD (AGAATATTCAAGAATTCCTC), CASP1 (AAAGCTGTTTATCCGTTCCA), or GSDMD (CTTCCACTTCTACGATGCCA) and were cloned into a lentiviral lentiCRISPRv2 (GeCKO; Zhang Lab) backbone (Sanjana et al, 2014; Shalem et al, 2014). Lentiviral particles were produced in HEK293T by co-transfection of the construct with pVSVg (Addgene, 8454) and psPAX2 (Addgene, 12260) plasmids using a standard calcium phosphate protocol. Supernatants containing the lentiviral particles were collected after 48 h and added to THP-1 cells during a 1250× *g* centrifugation for 90 min in the presence of 8 μg/mL of polybrene (Santa Cruz, sc-134220). 1.25 µg/mL of puromycin was used to select infected cells. Single-cell sorting was performed with a FACS Aria III (Cytocell core facility, SFR Bonamy, BioCore, Inserm UMS 016, CNRS UAR 3556, Nantes, France), and viable single clones were amplified and tested by immunoblotting to control for negative expression of CYLD, CASP1, or GSDMD.

The following TrueGuide synthetic sgRNA (Thermofisher) were used: CASP3, ATGTCGATGCAGCAAACCTC; SPATA2, GATCAGCCGGAATCGATAAA. The negative sgRNA sequence (A35526) and TrueGuide Cas9 (A36499) were also used. Cells were electroporated using a specific Neon^TM^ Transfection System kit (MPK5000 and MPK1096, Thermofisher), according to TrueCut Cas9 Proteins protocol (Thermofisher). After 3–5 days, single-cell sorting was performed, and viable single clones were amplified and tested by immunoblotting to control for negative expression of CASP3 or SPATA2.

For the CRISPR Cas9-induced KO MDA-MB-231 cells, sgRNA sequences targeting human genes were designed using the MIT CRISPR design tool (http://crispr.mit.edu/). The guide sequences (*BAX*, AGTAGAAAAGGGCGACAACC; *BAK*, GCCATGCTGGTAGACGTGTA; *CASP3*, ATGTCGATGCAGCAAACCTC; *CASP8*, CACCGGCCTGGACTACATTCCGCAA) were cloned into the plentiCRISPRV2 vector (gift from Feng Zhang, Addgene plasmid 52961). Cells were selected using 1 μg/mL puromycin, and KOs were confirmed by immunoblot analysis.

For CRISPR/Cas9 knock-in (KI) editing, cell lines were generated by CRISPR/Cas9-mediated homologous directed recombination (HDR). Cells were electroporated with Cas9 protein (Alt-R™ S.p. HiFi Cas9 nuclease V3, IDT), sgRNA targeting CYLD exon 8 (GTCAACAGAAGACCCATTTA, Alt-R™ CRISPR-Cas9 sgRNA, IDT), and HDR template (CCTGGAAATAGAAACAGATCTGAATTATTTTATACCTTGAATGGCTCTTCTGTTGCCTCACAACCACAATCCAAATCAAAAAATACATGGTACATTG, Alt-R™ HDR Donor Oligos, IDT). To prevent the CRISPR/Cas9 system from re-cutting the edited sequence, two silent mutations were introduced in the HDR template (1453 A > G, L358L and 1459 G > C, G360G). 3–5 days after electroporation, single-cell sorting was performed. Three weeks later, DNA was isolated from a fraction of each well, and the mutation was confirmed by sequencing after PCR amplification of the specific region.

Site-directed mutagenesis was conducted on the MSCV-TAP(FLAG-HA) CYLD plasmid, a gift from S. Elledge (Addgene plasmid 60031), to substitute the aspartic acid (D) residue at positions 215, 345, or 364 with an alanine (A).

### Cell death induction and measurement of cell viability

For apoptosis induction, cells were treated with ABT-737 in combination with S63845 or with Raptinal (10 μM). Cell viability was measured using CellTiter Glo and CASP3/7 Glo (Promega) following the manufacturer’s instructions. To induce pyroptosis, cells were first primed with LPS (1 μM) for 3 h before stimulation with Nigericin (10 μM). The release of HMGB1 fused to Lucia was measured per the manufacturer’s instructions. In some experiments, cells were pretreated for 30 min with MG132 (25 μM), Bafilomycin A1 (200 nM), MCC950 (2 μM), or MLT-985 (2 μM) before stimulation.

### Live-cell imaging

24-well plates pre-coated with 0.02% poly-L-lysine in 0.22 µm-filtered PBS, incubated for 30 min at 37 °C, and washed once with PBS. THP-1 cells were seeded at 5 × 10^5^ cells per well in 500 µL of complete RPMI 1640 medium and allowed to attach for 2 h at 37 °C. Apoptosis was induced with ABT-737 and S63845 (1 µM each) in the presence of Annexin V Alexa Fluor 594 (Invitrogen, A13203). When indicated, THP-1 cells were pretreated with Q-VD-OPh (10 µM) for 30 min before stimulation. Bright-field and red fluorescence images were acquired every 30 min for 2 h at ×20 magnification using the IncuCyte S3 system (Sartorius). Each experiment was performed in duplicate, and three images were acquired per condition.

### Lysate preparation, immunoblotting, and immunoprecipitations

Stimulations were terminated with two washes with ice-cold PBS. Samples were lysed with RIPA buffer [25 mM Tris- HCl (pH 7.4), 150 mM NaCl, 0.1% SDS, 0.5% Na-Deoxycholate, 1% NP-40, 1 mM EDTA] supplemented with 1× Halt Protease Inhibitor cocktail (ThermoFisher Scientific), for 30 min on ice. Samples were centrifuged at 10,000× *g*, and protein concentration was determined with a BCA kit (UP40840A; Interchim). In total, 5–10 μg of proteins were incubated with 2× Laemmli buffer (Life Technologies) at 95 °C for 3 min. Lysates were resolved by SDS-polyacrylamide gel electrophoresis (SDS-PAGE) using 3–15% Tris-Acetate or 5–20% Tris-Glycine gels and transferred onto nitrocellulose membranes (GE Healthcare).

For immunoprecipitations, cell lysates were precleared with Protein G Sepharose (Sigma) for 30 min before incubation with 1 μg of antibodies to SPATA2 (Bethyl, A302-493 AM) or with nonrelevant antibodies for 2 h at 4 °C with rotation.

HEK293T cells were transfected using a standard calcium phosphate protocol with 1 μg of plasmids encoding for FLAG-GSDMD, a gift from J. Lieberman (Addgene plasmid 80950), or MSCV-TAP(FLAG-HA) CYLD. Lysates were incubated with FLAG M2 Affinity Resin (Sigma, #A2220) for 2 h under rotation at 4 °C. Samples were washed 3 times with RIPA buffer and once with reaction buffer [25 mM HEPES, 0.1% (w/v) CHAPS, 10 mM DTT]. FLAG-tagged proteins were eluted by incubation with 3X-FLAG peptide (F4799, 100 μg/mL) for 1 h at 4 °C with rotation.

The following antibodies were purchased from Santa Cruz: CYLD (H-6, sc-137139), PARP (sc-8007), GAPDH (sc-32233), SPATA2 (sc-515283), HMGB1 (sc-56698), and Ubiquitin (sc-8017). Antibodies to BAK (12105), CASP3 (9662), active CASP7 (8438), CASP8 (9746), active CASP1 (4199), IκBα (9242), phospho-IκBα (9246), IRF3 (4302), and p62 (88588) were from Cell Signaling Technologies. Antibodies to IFIT1 (23247-1-AP), α-Tubulin (66031-1-1 g), BAX (50599-2), and IRF3 (113-12-1-AP) were obtained from Proteintech. Antibodies to CYLD (clone 733, 43-7700, Invitrogen), SPATA2 (A302-493A, Bethyl Laboratories), phospho-IRF3 Ser386 (ab76493, Abcam), and GSDMD (HPA044487, Atlas antibodies) were also used.

### In vitro cleavage assays and qualitative DUB assays

For in vitro cleavage assays, recombinant proteins or immunopurified FLAG-tagged proteins were incubated with recombinant CASPs at 37 °C for 30 min in reaction buffer [25 mM HEPES, 0.1% (w/v) CHAPS, 10 mM DTT, pH 7.5]. Samples were then resolved by SDS-PAGE.

The cell-free activation of CASPs with cytochrome c and dATP was performed as previously described (Slee et al, 1999, 2001). In brief, 20 × 10^6^ THP-1 cells were washed twice with PBS 1X and resuspended for 5 min on ice with 400 μL fractionation buffer [20 mM Hepes-KOH pH 7.5, 10 mM KCl, 1.5 mM MgCl_2_, 1 mM EDTA, 1 mM EGTA, 1 mM DTT] supplemented with 1× Halt Protease Inhibitor cocktail. Cells were broken by 10 passages in a 30 G syringe, samples were centrifuged for 15 min at 15,000×* g*, and protein concentration was determined. MCF7 cells in fractionation buffer were lysed by three cycles of freeze and thaw. 10 μg samples were incubated with 1 mM dATP and 50 μg/mL cytochrome c for 3 h at 37 °C. Samples were then resolved by SDS-PAGE.

Ubiquitin chain cleavage assays were performed as previously described (Elliott et al, 2021) using purified Lys63- and Met1-linked tetra ubiquitin chains (Ub_4_). Final concentrations per sample were: CYLD [20 nM], CASP [250 nM], K63- or Met1-Ub_4_ [0.2 mg/mL], prepared with dilution buffer [20 mM Tris (pH 7.4), 200 mM NaCl, 10 mM DTT]. Reactions were performed in assay buffer [final concentration: 50 mM Tris (pH 7.4), 50 mM NaCl, 5 mM DTT] at 37 °C, and the reaction was initiated through the addition of DUB to ubiquitin substrate. At designated time points, samples were taken, and the reaction was stopped by adding 2× Laemmli buffer (Life Technologies) at 95 °C for 3 min. Samples were then resolved by SDS-PAGE and silver-stained (Thermofisher, LC6070).

### qPCRs analyses

Before RNA extraction, cells (2 × 10^6^) were treated with ABT-737 plus S63845 (1 µM each) for 2 h. RNA extractions were performed using the Macherey-Nagel RNA Plus NucleoSpin kit on biological triplicate samples. The RNA was subsequently reverse transcribed using the Maxima First Strand cDNA synthesis kit (Thermo Scientific), and 25 ng of the resulting cDNA was amplified by qPCR using PerfeCTa SYBR Green SuperMix Low ROX (QuantaBio). Data were analyzed using the 2^-ΔΔCt^ method and normalized by the housekeeping genes ACTB and HPRT1. The following primers were used: ACTB forward, 5ʹ-GGACTTCGAGCAAGAGATGG-3ʹ; ACTB reverse, 5ʹ-AGCACTGTGTTGGCGTACAG-3ʹ; BIM forward, 5ʹ-TAAGTTCTGAGTGTGACCGAGA-3ʹ; BIM reverse, 5’-GCTCTGTCTGTAGGGAGGTAGG-3ʹ; CXCL10 forward, 5ʹ-GAAAGCAGTTAGCAAGGAAAGGTG-3ʹ; CXCL10 reverse, 5ʹ-ATGTAGGGAAGTGATGGGAGAGG-3ʹ; CYLD forward, 5ʹ-AAGGCTACAGGATCTACCTCAG-3ʹ; CYLD reverse, 5ʹ-GTGGTTGTGAGTCAACAGAAGA-3ʹ; HPRT1 forward, 5ʹ-TGACACTGGCAAAACAATGCA-3ʹ; HPRT1 reverse, 5ʹ-GGTCCTTTTCACCAGCAAGCT-3ʹ; IFIT1 forward, 5ʹ-GCGCTGGGTATGCGATCTC-3ʹ; IFIT1 reverse, 5ʹ- CAGCCTGCCTTAGGGGAAG-3ʹ; IFNB1 forward, 5ʹ-GCTTGGATTCCTACAAAGAAGCA-3ʹ; IFNB1 reverse, 5ʹ-ATAGATGGTCAATGCGGCGTC-3ʹ; NOXA forward, 5ʹ-ACCAAGCCGGATTTGCGATT-3ʹ; NOXA reverse, 5ʹ-ACTTGCACTTGTTCCTCGTGG-3ʹ; OASL forward, 5ʹ-CTGATGCAGGAACTGTATAGCAC-3ʹ; OASL reverse, 5ʹ- CACAGCGTCTAGCACCTCTT-3ʹ; PUMA forward, 5ʹ-GACCTCAACGCACAGTACGAG-3ʹ; PUMA reverse, 5ʹ-AGGAGTCCCATGATGAGATTGT-3ʹ; SPATA2 forward, 5ʹ-CGGCCAAGGACTACTACAAGC-3ʹ; SPATA2 reverse, 5ʹ-GGATGGGCGGATGATCTCA-3ʹ.

### Statistical analysis and reproducibility

Statistical analyses were performed using GraphPad Prism 10 software. The significance and number of repeats are indicated in the figure legends.

## Supplementary information

Peer Review File

Source data Fig. 1

Source data Fig. 2

Source data Fig. 3

Source data Fig. 4

Figure EV1 Source Data

Figure EV2 Source Data

Figure EV3 Source Data

Figure EV4 Source Data

Figure EV5 Source Data

Expanded View Figures

## Data Availability

This study includes no data deposited in external repositories. The source data of this paper are collected in the following database record: biostudies:S-SCDT-10_1038-S44319-026-00876-4.
