# Supplementary material for: Caspase-3 cleaves CYLD to restrict interferon signaling during mitochondrial apoptosis
Source: EMBO Rep. 2026 Jul 15;27(17):4999–5021. doi: 10.1038/s44319-026-00876-4 (PMC13554258; doi:10.1038/s44319-026-00876-4)
Supplement: Supplementary file 1 [file 44319_2026_876_MOESM11_ESM.pdf]

Expanded View Figures

Figure EV1. CYLD cleavage is associated with hallmarks of apoptosis.

Related to Fig. 1. (**A**) Representative IncuCyte bright-field images of THP-1 cells treated with increasing doses of ABT-737 and S63845 (A/S) for 0 h and 1 h (20X magnification). When indicated, cells were pretreated with the pan-caspase inhibitor Q-VD-OPh (10 µM, 30 min). (**B**, **C**) Real-time quantification of annexin V-positive THP-1 cells (red objects) treated as in (**A**) using IncuCyte ($n = 3$; mean ± SEM; ****$P < 0.0001$; two-way ANOVA). P values for each comparison: 0.125 µM, untreated vs 30 min: $P = 0.6958$; 0.125 µM, untreated vs 60 min: $P = 0.6395$; 0.125 µM, untreated vs 90 min: $P = 0.0126$; 0.125 µM, untreated vs 120 min: $P < 0.0001$; 0.250 µM, untreated vs 30 min: $P = 0.5936$; 0.250 µM, untreated vs 60 min: $P = 0.1943$; 0.250 µM, untreated vs 90 min: $P = 0.011$; 0.250 µM, untreated vs 120 min: $P < 0.0001$; 0.5 µM, untreated vs 30 min: $P = 0.9921$; 0.5 µM, untreated vs 60 min: $P = 0.0011$; 0.5 µM, untreated vs 90 min: $P < 0.0001$; 0.5 µM, untreated vs 120 min: $P < 0.0001$; 1 µM, untreated vs 30 min: $P = 0.4030$; 1 µM, untreated vs 60 min: $P < 0.0001$; 1 µM, untreated vs 90 min: $P < 0.0001$; 1 µM, untreated vs 120 min: $P < 0.0001$. (**D**) Immunoblot analysis of THP-1 cells pretreated or not with Q-VD-OPh (10 µM, 30 min) and stimulated with A/S as indicated for 1 h. Closed and open triangles indicate full-length and cleaved proteins. Cl.CASP7, cleaved CASP7. Numbers to the right indicate molecular mass markers. (**E**) CASP3/7 catalytic activity was measured by luminescent substrate cleavage (CASP Glo assay) in THP-1 cells pretreated or not with Q-VD-OPh (10 µM, 30 min) and treated with escalating concentrations of A/S, as indicated ($n = 4$; mean ± SEM; ****$P < 0.0001$; two-way ANOVA). P values for each comparison: 0.125 µM, control vs Q-VD-Oph: $P < 0.0001$; 0.25 µM, control vs Q-VD-Oph: $P < 0.0001$; 0.5 µM, control vs Q-VD-Oph: $P < 0.0001$; 1 µM, control vs Q-VD-Oph: $P < 0.0001$. (**F**) Dose–response curves of the indicated THP-1 cells treated with escalating doses of ABT-737 and S63845 (A/S) for 24 h ($n = 6$; mean ± SEM; extra sum-of-squares F test; P value < 0.0001). (**G**, **H**) Immunoblot analysis of THP-1 cells treated as indicated with etoposide (VP16) or staurosporine (STS) for 24 h. Closed and open triangles indicate full-length and cleaved proteins. (**I**) THP-1 cells were pretreated with MG132 (25 µM) or Bafilomycin A1 (Baf A1, 200 nM) for 30 min and exposed to A/S (1 µM each) for 1 h. Cell lysates were prepared and analyzed by immunoblotting with antibodies specific to the indicated proteins. The presented immunoblots are representative of at least three independent experiments. Source data are available online for this figure.

© The Author(s)

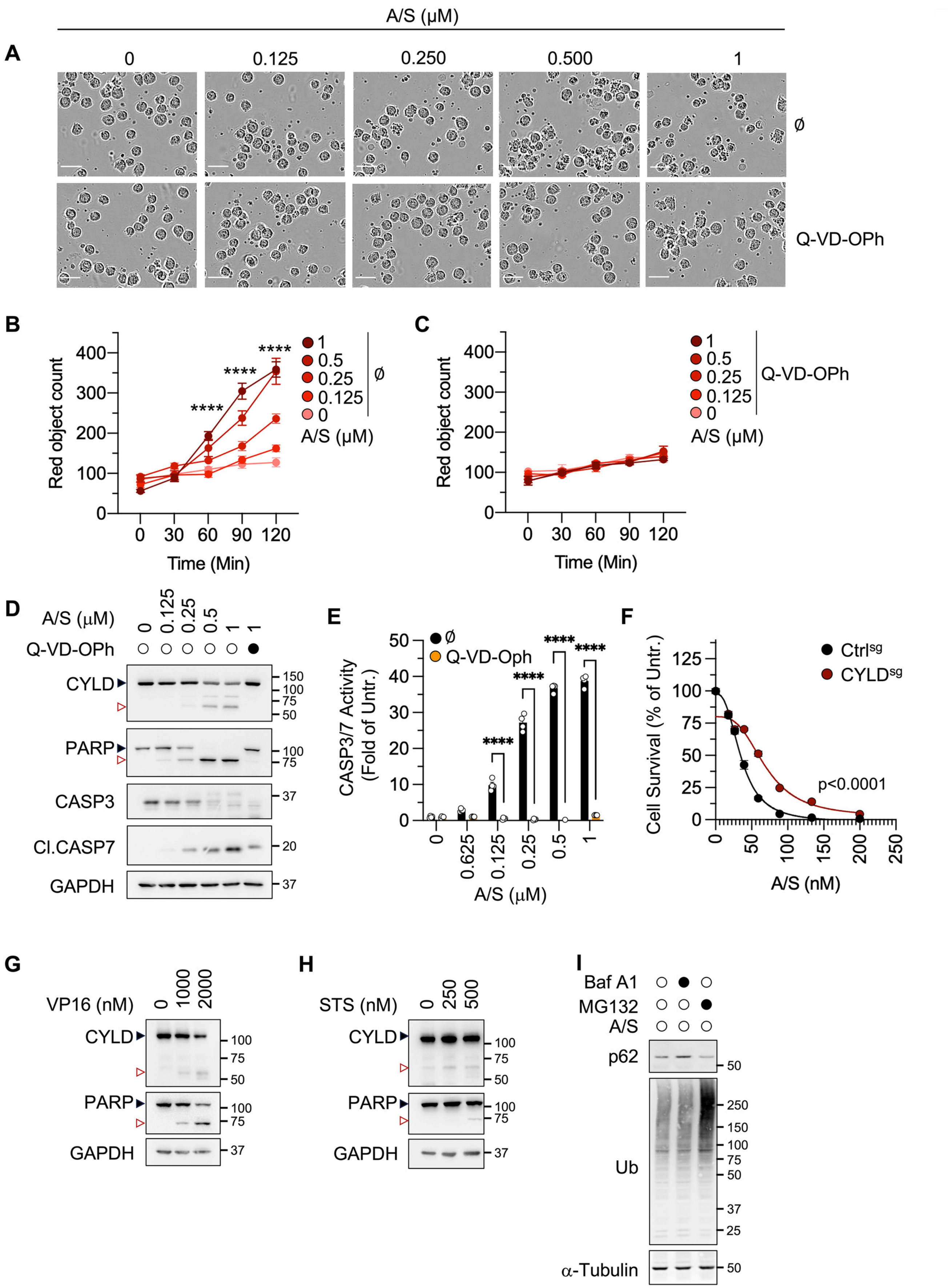
A/S (μM)
A
0
0.125
0.250
0.500
1
Ø
Q-VD-OPh
B
Red object count
Time (Min)
A/S (μM)
C
Red object count
Time (Min)
Q-VD-OPh
A/S (μM)
D
A/S (μM)
Q-VD-OPh
CYLD
PARP
CASP3
Cl.CASP7
GAPDH
E
CASP3/7 Activity (Fold of Untr.)
Ø
Q-VD-Oph
A/S (μM)
F
Cell Survival (% of Untr.)
Ctrl sg
CYLD sg
p<0.0001
A/S (nM)
G
VP16 (nM)
CYLD
PARP
GAPDH
H
STS (nM)
CYLD
PARP
GAPDH
I
Baf A1
MG132
A/S
p62
Ub
α-Tubulin

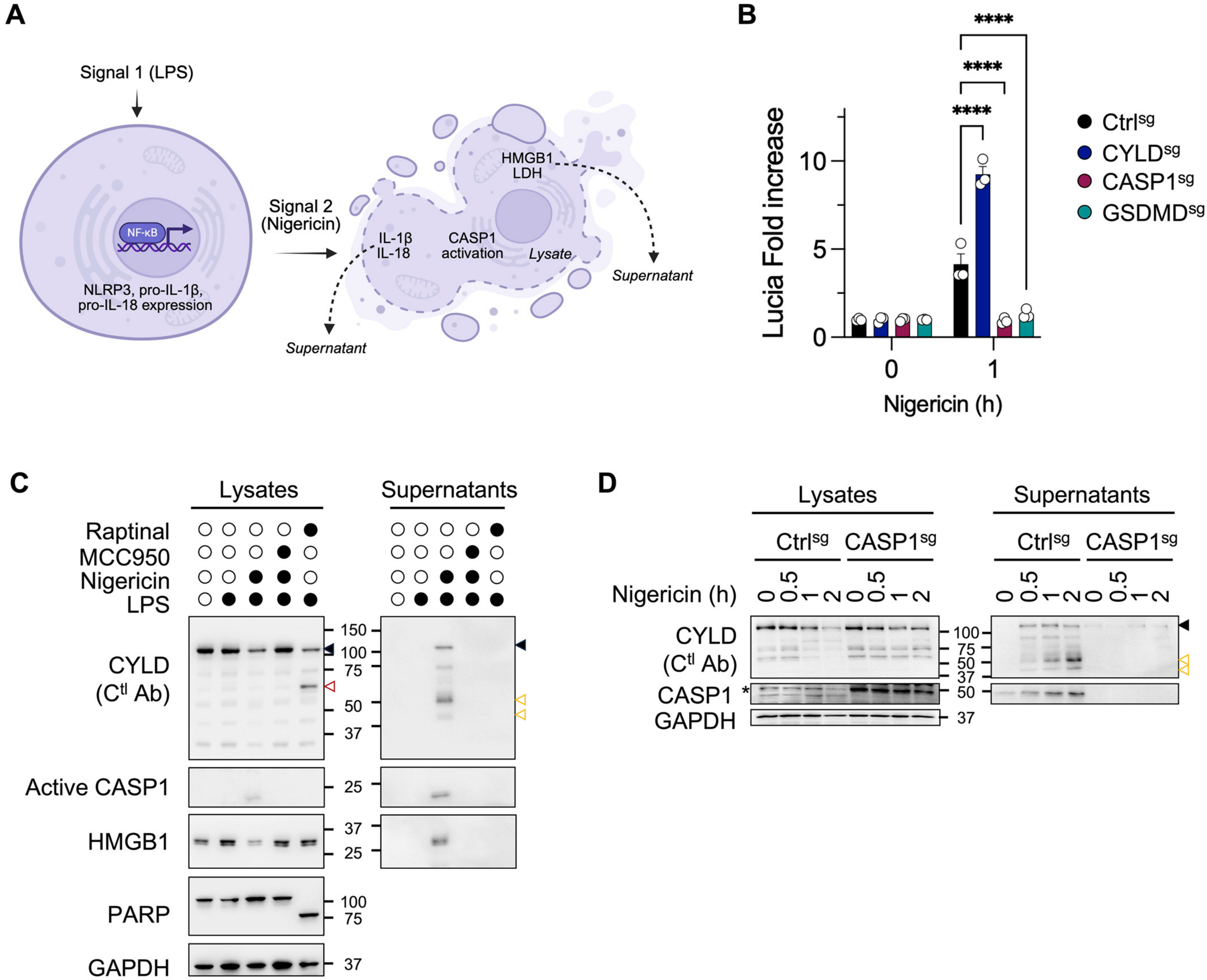

Figure EV2. CYLD is differentially cleaved during apoptosis and pyroptosis.

Related to Fig. 1. (**A**) Workflow to induce pyroptosis in monocytic cells. (**B**) THP-1 cells stably expressing HMGB1-Lucia deleted with sgRNAs for CYLD (CYLD^{sg}), CASP1 (CASP1^{sg}), GSDMD (GSDMD^{sg}), or their control counterparts (Ctrl^{sg}) were primed with LPS (1 µg/mL) for 3 h before stimulation with Nigericin (10 µM) for 1 h. The release of HMGB1-Lucia was assessed in cell supernatants ($n = 3$; mean ± SEM; ****$P < 0.0001$; two-way ANOVA). P values for each comparison: Ctrl^{sg} vs CYLD^{sg}: $P < 0.0001$; Ctrl^{sg} vs CASP1^{sg}: $P < 0.0001$; Ctrl^{sg} vs GSDMD^{sg}: $P < 0.0001$. (**C**) Immunoblot analysis of THP-1 cells primed or not with LPS (1 µg/mL) for 3 h before treatment with Nigericin (10 µM) or Raptinal (10 µM) for 1 h. When indicated, cells were pretreated with the NLRP3 inhibitor MCC950 (2 µM) for 30 min. Closed and open symbols indicate full-length and cleaved CYLD. (**D**) Immunoblot analysis of Ctrl^{sg} and CASP1^{sg} THP-1 cells treated as in (**B**). * indicates a nonspecific band. The presented immunoblots are representative of at least three independent experiments. Source data are available online for this figure.

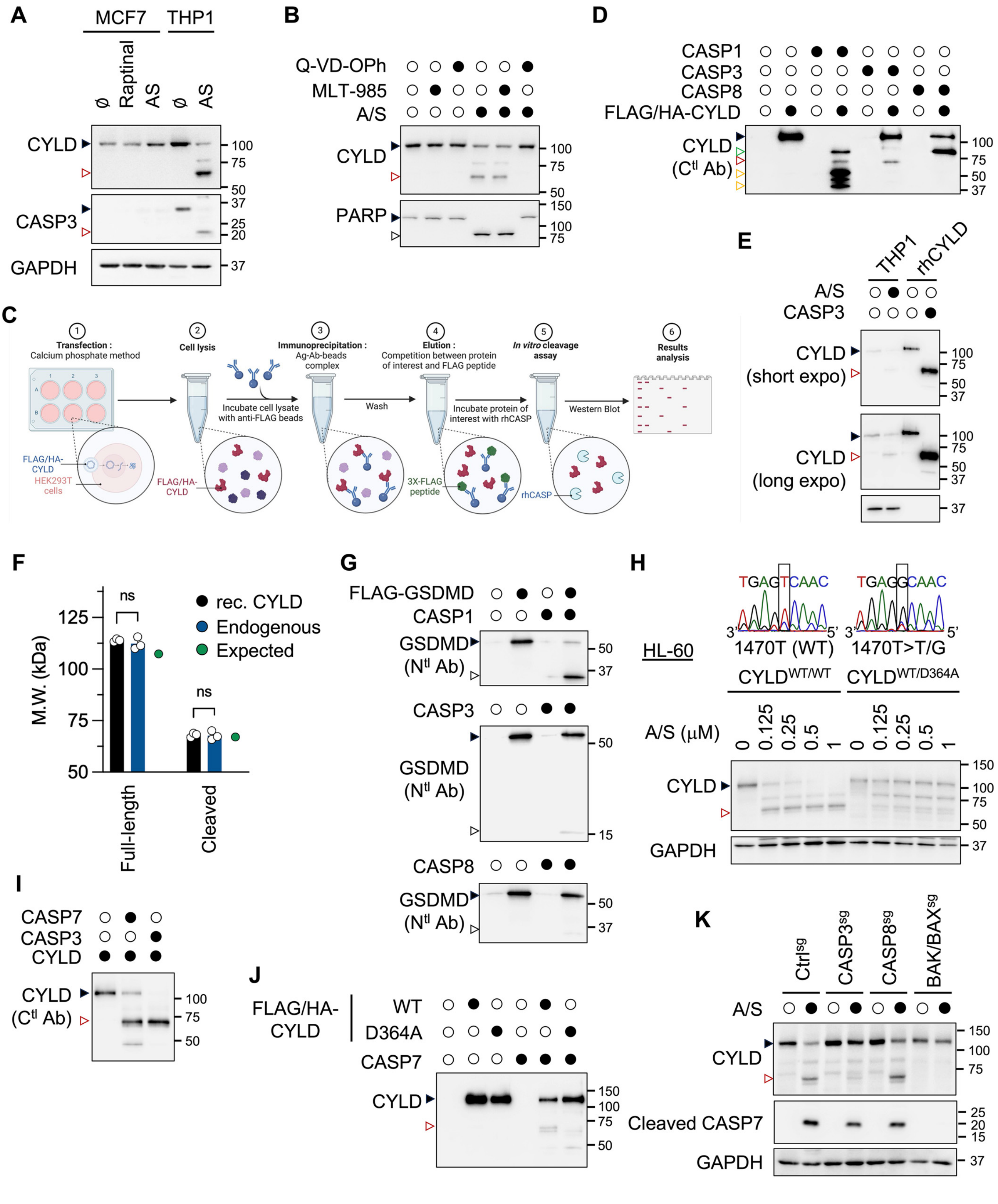
A
MCF7
THP1
ø
Raptinal
AS
ø
AS
CYLD
CASP3
GAPDH
100
75
50
37
25
20
37
B
Q-VD-OPh
MLT-985
A/S
CYLD
PARP
100
75
50
150
100
75
D
CASP1
CASP3
CASP8
FLAG/HA-CYLD
CYLD
(Ctl Ab)
100
75
50
37
E
THP1
rhCYLD
A/S
CASP3
CYLD
(short expo)
CYLD
(long expo)
100
75
50
37
C
1 Transfection : Calcium phosphate method
2 Cell lysis
3 Immunoprecipitation : Ag-Ab-beads complex
4 Elution : Competition between protein of interest and FLAG peptide
5 In vitro cleavage assay
6 Results analysis
Incubate cell lysate with anti-FLAG beads
Wash
Incubate protein of interest with rhCASP
Western Blot
FLAG/HA-CYLD
HEK293T cells
FLAG/HA-CYLD
3X-FLAG peptide
rhCASP
F
M.W. (kDa)
125
100
75
50
ns
ns
rec. CYLD
Endogenous
Expected
Full-length
Cleaved
G
FLAG-GSDMD
CASP1
GSDMD
(Ntl Ab)
CASP3
GSDMD
(Ntl Ab)
CASP8
GSDMD
(Ntl Ab)
50
37
50
15
50
37
H
HL-60
TGAGTCAAC
TGAGGCAAC
3'
5'
1470T (WT)
1470T>T/G
CYLDWT/WT
CYLDWT/D364A
A/S (μM)
0
0.125
0.25
0.5
1
CYLD
GAPDH
150
100
75
50
37
I
CASP7
CASP3
CYLD
CYLD
(Ctl Ab)
100
75
50
J
FLAG/HA-CYLD
WT
D364A
CASP7
CYLD
150
100
75
50
K
Ctrlsg
CASP3sg
CASP8sg
BAK/BAXsg
A/S
CYLD
Cleaved CASP7
GAPDH
150
100
75
25
20
15
37

Figure EV3. CYLD cleavage by caspases.

Related to Figs. 2 and 3. (**A**) Immunoblot analysis of MCF7 cells treated with Raptinal (10 µM) or ABT-737 and S63845 (A/S, 10 µM each) for 3 h. As a control, THP-1 cells exposed to A/S (1 µM each, 1 h) were used. Cell lysates were prepared, and immunoblotting was performed as indicated. Closed and open triangles indicate full-length and cleaved proteins. Numbers to the right indicate molecular mass markers. (**B**) Immunoblot analysis of THP-1 cells pretreated with MLT-985 (2 µM) or Q-VD-OPh (10 µM) for 30 min before exposure to A/S (1 µM each) for 1 h. (**C**) Workflow of the in vitro CASP cleavage assay of immunopurified CYLD. (**D**, **E**) In vitro cleavage of immunopurified CYLD, as in (**C**), incubated with the indicated recombinant human CASPs. In (**E**), cell lysates from THP-1 treated with ABT-737 and S63845 (A/S, 1 µM each) for 1 h were also analyzed. (**F**) Measured and expected molecular weights of CYLD and its cleaved fragment ($n = 3$; mean ± SEM; ns, not significant; ANOVA). *P* values for each comparison: Full-length, rhCYLD vs endogenous: $P = 0.9274$; Cleaved, rhCYLD vs endogenous: $P > 0.9999$. Rec., recombinant. (**G**) in vitro cleavage of immunopurified GSDMD, as in (**A**), incubated with the indicated CASPs. (**H**) Immunoblot analysis of control cells ($CYLD^{WT/WT}$) or knock-in HL-60 cells engineered to express heterozygous D364A mutation ($CYLD^{WT/D364A}$), treated with ABT-737 and S63845 (A/S) for 1 h. (**I**) in vitro cleavage of CYLD incubated with the indicated CASPs. (**J**) HEK293T cells were transfected with the indicated FLAG/HA-CYLD plasmids. After 24 h, cell lysates were prepared and immunoprecipitated with FLAG antibodies. Samples were eluted with FLAG peptides before incubation with CASP7 as indicated. Samples were analyzed by immunoblotting as indicated. (**K**) Immunoblot analysis of $Ctrl^{sg}$, $CASP3^{sg}$, $CASP8^{sg}$, or BAX/BAK^{sg} MDA-MB-231 cells treated with ABT-737 and S63845 (A/S, 10 µM each) for 2 h. The presented immunoblots are representative of at least three independent experiments. Source data are available online for this figure.

© The Author(s)

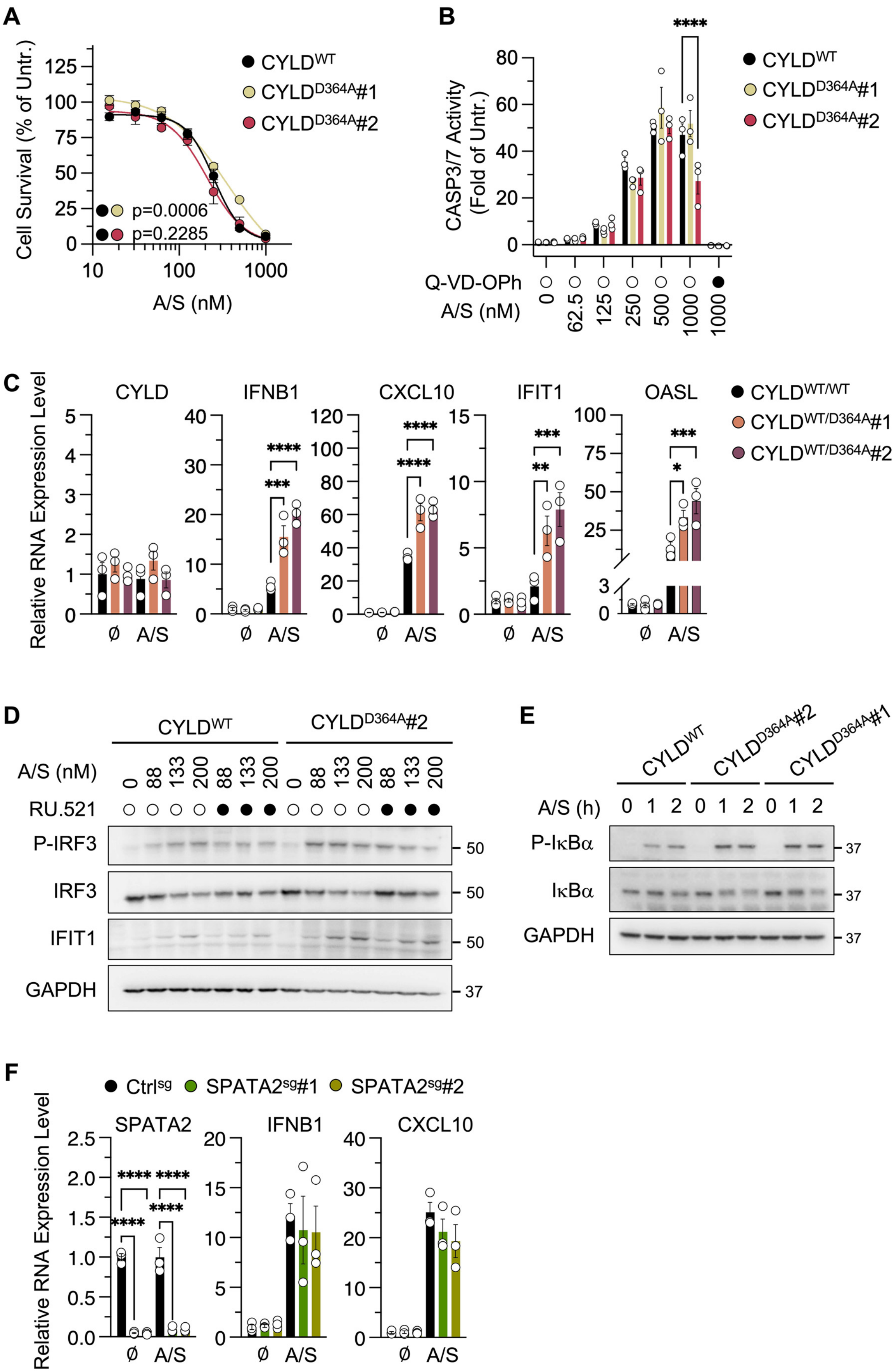
A
Cell Survival (% of Untr.)
A/S (nM)
CYLD^{WT}
CYLD^{D364A}#1
CYLD^{D364A}#2
p=0.0006
p=0.2285
B
CASP3/7 Activity (Fold of Untr.)
****
Q-VD-OPh
A/S (nM)
0 62.5 125 250 500 1000 1000
C
Relative RNA Expression Level
CYLD
IFNB1
CXCL10
IFIT1
OASL
Ø A/S
CYLD$^{WT/WT}$
CYLD$^{WT/D364A}$#1
CYLD$^{WT/D364A}$#2
D
CYLD^{WT}
CYLD^{D364A}#2
A/S (nM)
0 88 133 200 88 133 200 0 88 133 200 88 133 200
RU.521
P-IRF3
IRF3
IFIT1
GAPDH
E
CYLD^{WT}
CYLD^{D364A}#2
CYLD^{D364A}#1
A/S (h)
0 1 2 0 1 2 0 1 2
P-IκBα
IκBα
GAPDH
F
Ctrl^{sg}
SPATA2^{sg}#1
SPATA2^{sg}#2
Relative RNA Expression Level
SPATA2
IFNB1
CXCL10
Ø A/S

Figure EV4. Cells expressing CASP3-resistant CYLD display enhanced interferon signaling.

Related to Fig. 4. (**A**) Dose–response curves in the indicated THP-1 cell genotypes treated with escalating doses of ABT-737 and S63845 (A/S, 15.625, 31.25, 62.5, 125, 250, 500, and 1000 nM) for 1 h. Metabolic cell survival was assessed by CellTiter Glo ($n = 4$; mean ± SEM; extra sum-of-squares F test; P values for each comparison: $CYLD^{WT}$ vs $CYLD^{D364A\#1}$: $P = 0.0006$, $CYLD^{WT}$ vs $CYLD^{D364A\#2}$: $P = 0.2285$). (**B**) CASP3/7 Glo assay in the indicated THP-1 cell genotypes treated with escalating concentrations of A/S, as indicated. When indicated, cells were pretreated with Q-VD-OPh (10 µM, 30 min) ($n = 3$; mean ± SEM; two-way ANOVA; ****$P < 0.0001$; P values for $CYLD^{WT}$ vs $CYLD^{D364A\#2}$, A/S 1000 nM: $P < 0.0001$). (**C**) qPCR analysis of the indicated genes in the indicated HL-60 cell genotypes treated with ABT-737 and S63845 (A/S, 1 µM each) for 2 h ($n = 3$; mean ± SEM; *$P < 0.05$, **$P < 0.01$, ***$P < 0.001$, ****$P < 0.0001$; one-way ANOVA). P values for each comparison (A/S treatment): INFB1, $CYLD^{WT}$ vs $CYLD^{D364A\#1}$: $P = 0.0001$; INFB1, $CYLD^{WT}$ vs $CYLD^{D364A\#2}$: $P < 0.0001$; CXCL10, $CYLD^{WT}$ vs $CYLD^{D364A\#1}$: $P < 0.0001$; CXCL10, $CYLD^{WT}$ vs $CYLD^{D364A\#2}$: $P < 0.0001$; OASL, $CYLD^{WT}$ vs $CYLD^{D364A\#1}$: $P = 0.0218$; OASL, $CYLD^{WT}$ vs $CYLD^{D364A\#2}$: $P = 0.0010$; IFIT1, $CYLD^{WT}$ vs $CYLD^{D364A\#1}$: $P = 0.0071$; IFIT1, $CYLD^{WT}$ vs $CYLD^{D364A\#2}$: $P = 0.0005$. (**D**) Immunoblot analysis of THP-1 cell genotypes pretreated or not with the cGAS inhibitor RU.521 (10 µM, 30 min) and stimulated with A/S as indicated for 24 h. Numbers to the right indicate molecular mass markers. (**E**) Immunoblot analysis of the indicated THP-1 cell genotypes exposed to A/S (1 µM each) for 1 and 2 h. (**F**) qPCR analysis of SPATA2, IFNB1, and CXCL10 in the indicated THP-1 cell genotypes treated with ABT-737 and S63845 (A/S, 1 µM each) for 2 h ($n = 3$; mean ± SEM; ****$P < 0.0001$; one-way ANOVA). P values for each comparison: SPATA2, Untreated $Ctrl^{sg}$ vs $SPATA2^{sg\#1}$: $P < 0.0001$; SPATA2, Untreated SPATA2, $Ctrl^{sg}$ vs $SPATA2^{sg\#2}$: $P < 0.0001$; SPATA2, A/S $Ctrl^{sg}$ vs $SPATA2^{sg\#1}$: $P < 0.0001$; SPATA2, A/S $Ctrl^{sg}$ vs $SPATA2^{sg\#2}$: $P < 0.0001$. The presented immunoblots are representative of at least three independent experiments. Source data are available online for this figure.

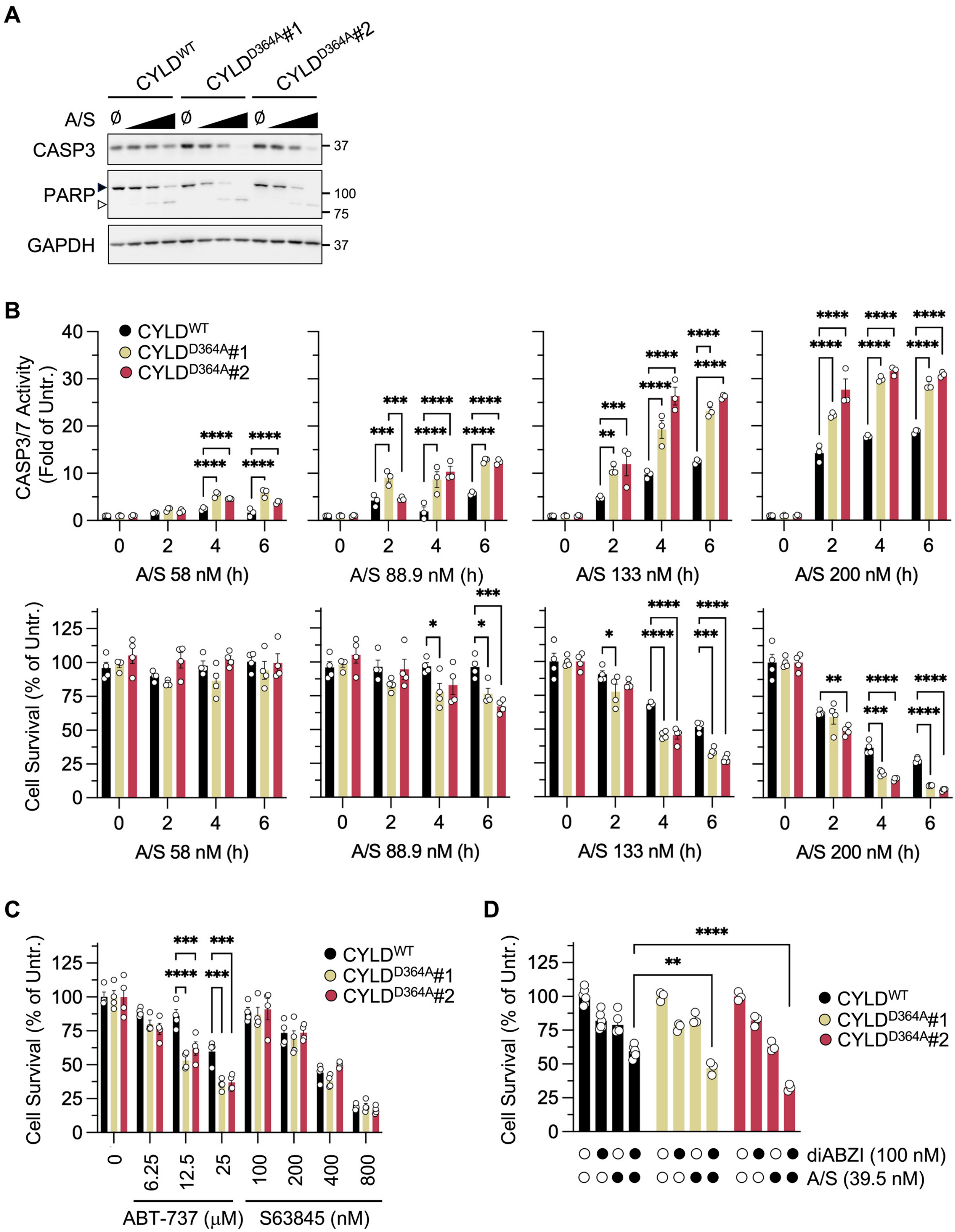
A
CYLDWT
CYLDD364A#1
CYLDD364A#2
A/S
CASP3
PARP
GAPDH
37
100
75
37
B
CYLDWT
CYLDD364A#1
CYLDD364A#2
CASP3/7 Activity (Fold of Untr.)
A/S 58 nM (h)
A/S 88.9 nM (h)
A/S 133 nM (h)
A/S 200 nM (h)
Cell Survival (% of Untr.)
C
Cell Survival (% of Untr.)
ABT-737 (μM)
S63845 (nM)
D
Cell Survival (% of Untr.)
diABZI (100 nM)
A/S (39.5 nM)

 © The Author(s)

Figure EV5. Cells expressing CASP3-resistant CYLD display enhanced cell death.

Related to Fig. 4. (**A**) Immunoblot analysis of the indicated THP-1 cell genotypes treated with ABT-737 and S63845 (A/S, 39.5, 59, 89 nM each) for 24 h. Closed and open triangles indicate full-length and cleaved PARP. (**B**) CASP3/7 Glo and CellTiter Glo assays of cells in the indicated THP-1 cell genotypes treated for 0, 2, 4, and 6 h with the indicated doses of ABT-737 and S63845 (A/S) ($n = 3$ for CellTiter Glo and $n = 4$ for CASP3/7 Glo; mean ± SEM; *$P < 0.05$, **$P < 0.01$, ***$P < 0.001$, ****$P < 0.0001$; two-way ANOVA). For CASP3/7 Glo, P values for each comparison: 58 nM 4 h, CYLD^{WT} vs CYLD$^{D364A\#1}$: $P < 0.0001$; 58 nM 4 h, CYLD^{WT} vs CYLD$^{D364A\#2}$: $P < 0.0001$; 58 nM 6 h, CYLD^{WT} vs CYLD$^{D364A\#1}$: $P < 0.0001$; 58 nM 6 h, CYLD^{WT} vs CYLD$^{D364A\#2}$: $P < 0.0001$; 88.9 nM 2 h, CYLD^{WT} vs CYLD$^{D364A\#1}$: $P = 0.0003$; 88.9 nM 2 h, CYLD^{WT} vs CYLD$^{D364A\#2}$: $P = 0.9257$; 88.9 nM 4 h, CYLD^{WT} vs CYLD$^{D364A\#1}$: $P < 0.0001$; 88.9 nM 4 h, CYLD^{WT} vs CYLD$^{D364A\#2}$: $P < 0.0001$; 88.9 nM 6 h, CYLD^{WT} vs CYLD$^{D364A\#1}$: $P < 0.0001$; 88.9 nM 6 h, CYLD^{WT} vs CYLD$^{D364A\#2}$: $P < 0.0001$; 133 nM 2 h, CYLD^{WT} vs CYLD$^{D364A\#1}$: $P = 0.0027$; 133 nM 2 h, CYLD^{WT} vs CYLD$^{D364A\#2}$: $P = 0.0004$; 133 nM 4 h, CYLD^{WT} vs CYLD$^{D364A\#1}$: $P < 0.0001$; 133 nM 4 h, CYLD^{WT} vs CYLD$^{D364A\#2}$: $P < 0.0001$; 133 nM 6 h, CYLD^{WT} vs CYLD$^{D364A\#1}$: $P < 0.0001$; 133 nM 6 h, CYLD^{WT} vs CYLD$^{D364A\#2}$: $P < 0.0001$; 200 nM 2 h, CYLD^{WT} vs CYLD$^{D364A\#1}$: $P < 0.0001$; 200 nM 2 h, CYLD^{WT} vs CYLD$^{D364A\#2}$: $P < 0.0001$; 200 nM 4 h, CYLD^{WT} vs CYLD$^{D364A\#1}$: $P < 0.0001$; 200 nM 4 h, CYLD^{WT} vs CYLD$^{D364A\#2}$: $P < 0.0001$; 200 nM 6 h, CYLD^{WT} vs CYLD$^{D364A\#1}$: $P < 0.0001$; 200 nM 6 h, CYLD^{WT} vs CYLD$^{D364A\#2}$: $P < 0.0001$. For CellTiter Glo, P values for each comparison: 88.9 nM 4 h, CYLD^{WT} vs CYLD$^{D364A\#1}$: $P = 0.0242$; 88.9 nM 4 h, CYLD^{WT} vs CYLD$^{D364A\#2}$: $P = 0.1208$; 88.9 nM 6 h, CYLD^{WT} vs CYLD$^{D364A\#1}$: $P = 0.0164$; 88.9 nM 6 h, CYLD^{WT} vs CYLD$^{D364A\#2}$: $P = 0.0004$; 133 nM 2 h, CYLD^{WT} vs CYLD$^{D364A\#1}$: $P = 0.0131$; 133 nM 2 h, CYLD^{WT} vs CYLD$^{D364A\#2}$: $P = 0.2136$; 133 nM 4 h, CYLD^{WT} vs CYLD$^{D364A\#1}$: $P < 0.0001$; 133 nM 4 h, CYLD^{WT} vs CYLD$^{D364A\#2}$: $P < 0.0001$; 133 nM 6 h, CYLD^{WT} vs CYLD$^{D364A\#1}$: $P = 0.0004$; 133 nM 6 h, CYLD^{WT} vs CYLD$^{D364A\#2}$: $P < 0.0001$; 200 nM 2 h, CYLD^{WT} vs CYLD$^{D364A\#1}$: $P = 0.7666$; 200 nM 2 h, CYLD^{WT} vs CYLD$^{D364A\#2}$: $P = 0.0057$; 200 nM 4 h, CYLD^{WT} vs CYLD$^{D364A\#1}$: $P = 0.0001$; 200 nM 4 h, CYLD^{WT} vs CYLD$^{D364A\#2}$: $P < 0.0001$; 200 nM 6 h, CYLD^{WT} vs CYLD$^{D364A\#1}$: $P < 0.0001$; 200 nM 6 h, CYLD^{WT} vs CYLD$^{D364A\#2}$: $P < 0.0001$. (**C**) Metabolic cell survival measured by CellTiter Glo in the indicated THP-1 cell genotypes treated with escalating doses of ABT-737 or S63845 for 24 h ($n = 3$; mean ± SEM; ***$P < 0.001$, ****$P < 0.0001$; two-way ANOVA). P values for each comparison: ABT-737 12.5 μM, CYLD^{WT} vs CYLD$^{D364A\#1}$: $P < 0.0001$; ABT-737 12.5 μM, CYLD^{WT} vs CYLD$^{D364A\#2}$: $P = 0.0002$; ABT-737 25 μM, CYLD^{WT} vs CYLD$^{D364A\#1}$: $P = 0.0002$; ABT-737 25 μM, CYLD^{WT} vs CYLD$^{D364A\#2}$: $P = 0.0004$. (**D**) Cells as in (**A**) treated with A/S (39.5 nM each) in combination with diABZI (100 nM) for 24 h ($n = 3$ or 6; mean ± SEM; **$P < 0.01$, ****$P < 0.0001$; two-way ANOVA). P values for each comparison: CYLD^{WT} vs CYLD$^{D364A\#1}$: $P = 0.0051$; CYLD^{WT} vs CYLD$^{D364A\#2}$: $P < 0.0001$. The presented immunoblots are representative of at least three independent experiments. Source data are available online for this figure.

© The Author(s)
