# Supplementary material for: Caspase-3 cleaves CYLD to restrict interferon signaling during mitochondrial apoptosis
Source: EMBO Rep. 2026 Jul 15;27(17):4999–5021. doi: 10.1038/s44319-026-00876-4 (PMC13554258; doi:10.1038/s44319-026-00876-4)
Supplement: Supplementary file 2 [file 44319_2026_876_MOESM1_ESM.pdf]

EMBO reports

# Caspase-3 cleaves CYLD to restrict interferon signaling during mitochondrial apoptosis

Nicolas Bidere, Yanis Macé, Tiphaine Douanne, Jane Jardine, Kilian Trillet, Rosalie Moreau, Joanna Re, Cara Ellison, Nadine LAGUETTE, Paul Elliott, and Julie Gavard

*Corresponding author(s): Nicolas Bidere (nicolas.bidere@inserm.fr)*



## Review Timeline:

| | |
|---|---|
| Submission Date: | 17th Dec 25 |
| Editorial Decision: | 10th Feb 26 |
| Revision Received: | 12th May 26 |
| Editorial Decision: | 26th Jun 26 |
| Revision Received: | 1st Jul 26 |
| Accepted: | 6th Jul 26 |

*Editor: Martina Rembold*

## Transaction Report:

(Note: With the exception of the correction of typographical or spelling errors that could be a source of ambiguity, letters and reports are not edited. Depending on transfer agreements, referee reports obtained elsewhere may or may not be included in this compilation. Referee reports are anonymous unless the Referee chooses to sign their reports.)

Dear Dr. Bidere

Thank you for the submission of your manuscript to EMBO reports. We have now received the full set of referee reports that are all pasted below.

As you will see, the referees acknowledge that the findings are potentially interesting and that the conclusions are largely supported by the data. They have however a number of suggestions how to further strengthen your manuscript, such as a closer examination of caspase specificity, kinetics, and the addition of more functional and physiological data, which should be addressed.

I would thus like to invite you to revise your manuscript with the understanding that the referee concerns must be fully addressed and their suggestions taken on board. Please address all referee concerns in a complete point-by-point response. Acceptance of the manuscript will depend on a positive outcome of a second round of review. It is EMBO reports policy to allow a single round of major revision only and acceptance or rejection of the manuscript will therefore depend on the completeness of your responses included in the next, final version of the manuscript.

We realize that it is difficult to revise to a specific deadline. In the interest of protecting the conceptual advance provided by the work, we recommend a revision within 3 months (13th May 2026). Please discuss the revision progress ahead of this time with the editor if you require more time to complete the revisions.

You can either publish the study as a short report or as a full article. For short reports, the revised manuscript should not exceed 27,000 characters (including spaces but excluding materials & methods and references) and 5 main plus 5 expanded view figures. The results and discussion sections must further be combined, which will help to shorten the manuscript text by eliminating some redundancy that is inevitable when discussing the same experiments twice. For a normal article there are no length limitations, but it should have more than 5 main figures and the results and discussion sections must be separate. In both cases, the entire materials and methods must be included in the main manuscript file.

IMPORTANT NOTE: we perform an initial quality control of all revised manuscripts before re-review. Your manuscript will FAIL this control and the handling will be DELAYED if the following APPLIES:
1) A data availability section providing access to data deposited in public databases is missing. If you have not deposited any data, please add a sentence to the data availability section that explains that.
2) Your manuscript contains statistics and error bars based on n=2. Please use scatter blots in these cases. No statistics should be calculated if n=2.

When submitting your revised manuscript, please review the submission guidelines (https://link.springer.com/journal/44319/submission-guidelines#cms-Revised-submissions) and carefully review the instructions that follow below. Failure to include requested items will delay the evaluation of your revision.

1) a .docx formatted version of the manuscript text (including legends for main figures, EV figures and tables). Please make sure that the changes are highlighted to be clearly visible.

2) individual production quality figure files as .eps, .tif, .jpg (one file per figure). See hhttps://media.springernature.com/original/springer-cms/rest/v1/content/27825798/data/v1 for more info on how to prepare your figures.

3) We replaced Supplementary Information with Expanded View (EV) Figures and Tables that are collapsible/expandable online. A maximum of 5 EV Figures can be typeset. EV Figures should be cited as 'Figure EV1, Figure EV2" etc... in the text and their respective legends should be included in the main text after the legends of regular figures.

- For the figures that you do NOT wish to display as Expanded View figures, they should be bundled together with their legends in a single PDF file called *Appendix*, which should start with a short Table of Content. Appendix figures should be referred to in the main text as: "Appendix Figure S1, Appendix Figure S2" etc. See 'Expanded View' section of the submission guidelines.

- Additional Tables/Datasets should be labeled and referred to as Table EV1, Dataset EV1, etc. Legends have to be provided in a separate tab in case of .xls files. Alternatively, the legend can be supplied as a separate text file (README) and zipped together with the Table/Dataset file.

4) a .docx formatted letter INCLUDING the reviewers' reports and your detailed point-by-point responses to their comments. As part of the EMBO Press transparent editorial process, the point-by-point response is part of the Review Process File (RPF), which will be published alongside your paper.

5) a complete author checklist, which you can download from our author guidelines. Please insert information in the checklist

that is also reflected in the manuscript. The completed author checklist will also be part of the RPF.

6) Please note that all corresponding authors are required to supply an ORCID ID for their name upon submission of a revised manuscript (), which can be linked to your profile in the manuscript submission system.

7) Before submitting your revision, primary datasets produced in this study need to be deposited in an appropriate public database. Please remember to provide a reviewer password if the datasets are not yet public. The accession numbers and database should be listed in a formal "Data Availability" section placed after Methods. Please note that the Data Availability Section is restricted to new primary data that are part of this study. * Note - All links should resolve to a page where the data can be accessed. *

If your study has not produced novel datasets, please mention this fact in the Data Availability Section.

8) At EMBO Press we ask authors to provide source data for the main manuscript figures. You will receive a separate email with instructions for providing source data with your revised manuscript, including how to upload and organize the files.

9) Our journal also encourages inclusion of *data citations in the reference list* to directly cite datasets that were re-used and obtained from public databases. Data citations in the article text are distinct from normal bibliographical citations and should directly link to the database records from which the data can be accessed. In the main text, data citations are formatted as follows: "Data ref: Smith et al, 2001" or "Data ref: NCBI Sequence Read Archive PRJNA342805, 2017". In the Reference list, data citations must be labeled with "[DATASET]". A data reference must provide the database name, accession number/identifiers and a resolvable link to the landing page from which the data can be accessed at the end of the reference.

10) Regarding data quantification, the following points must be specified in each figure legend:

- the name of the statistical test used to generate error bars and P values,

- the number (n) of independent experiments (please specify technical or biological replicates) underlying each data point,

- the nature of the bars and error bars (s.d., s.e.m.),

- If the data are obtained from n Program fragment delivered error ``Can't locate object method "less" via package "than" (perhaps you forgot to load "than"?) at //ejpvfs23/sites23b/embor_www/letters/embor_decision_revise_and_review_sr_any.txt line 55.' 2, use scatter blots showing the individual data points.

Discussion of statistical methodology can be reported in the materials and methods section, but figure legends should contain a basic description of n, P and the test applied.

- Please also include scale bars in all microscopy images.

11) The journal requires a statement specifying whether or not authors have competing interests (defined as all potential or actual interests that could be perceived to influence the presentation or interpretation of an article). In case of competing interests, this must be specified in your disclosure statement.

12) All Materials and Methods need to be described in the main text using our 'Structured Methods' format, which is required for all research articles. According to this format, the Methods section includes a Reagents and Tools Table (listing key reagents, experimental models, software and relevant equipment and including their sources and relevant identifiers) followed by a Methods and Protocols section describing the methods using a step-by-step protocol format. The aim is to facilitate adoption of the methodologies across labs. More information on how to adhere to this format as well as a downloadable template (.docx) for the Reagents and Tools Table can be found in our author guidelines.

We would also welcome the submission of cover suggestions, or motifs to be used by our Graphics Illustrator in designing a cover.

As part of the EMBO publication's Transparent Editorial Process, EMBO reports publishes online a Review Process File (RPF) to accompany accepted manuscripts. This File will be published in conjunction with your paper and will include the referee reports, your point-by-point response and all pertinent correspondence relating to the manuscript.

You are able to opt out of this by letting the editorial office know (emboreports@embo.org). If you do opt out, the Review Process File link will point to the following statement: "No Review Process File is available with this article, as the authors have chosen not to make the review process public in this case."

I look forward to seeing a revised form of your manuscript when it is ready. Please use this link to submit your revision:

Yours sincerely,

Martina Rembold, PhD
Senior Editor
EMBO reports

=================

Referee #1:

This manuscript presents a beautiful and carefully executed biochemical study addressing an important question at the interface of apoptosis and inflammatory signaling. The work is technically strong and conceptually interesting, and the conclusions are largely well supported by the data.
A major strength of the study is the exceptional quality of the biochemical analyses. The western blots are very clear and convincing, and the biochemical experiments are thoughtfully designed and well controlled. In particular, the generation of CYLD mutants and the systematic analysis of their function in vitro represent a substantial experimental effort and are executed to a very high standard. The data are presented clearly, and the story is easy to follow. Overall, this is genuinely a very elegant and impressive piece of work.
The identification of CYLD as a CASP3 substrate, cleaved at a site distinct from the previously described CASP8 site, is novel and potentially important. The use of knock-in cells expressing CASP3-resistant CYLD strengthens the physiological relevance of the findings and supports the conclusion that CYLD proteolysis contributes to limiting interferon signaling and cell death during apoptosis.
That said, I remain somewhat puzzled by the assertion that the identified cleavage site represents a de facto CASP3-specific site. As caspases share overlapping substrate specificities and many substrates can be cleaved by multiple caspases under certain conditions, it is difficult to exclude potential contributions from other caspases based primarily on in vitro experiments. While the in vitro assays are informative and suggest differences in cleavage efficiency, they may not fully capture the complexity of caspase activity in cells. A more cautious discussion of caspase specificity, or additional experimental support, would strengthen this aspect of the manuscript. There is a last paragraph in the discussion, but I think more discussion ( or experimental data if available) of caspase-8 specificity versus caspase-3 is required. I think the link to caspase-8- activation platforms has to be made in this context and discussed accordingly.
More comments:
1. 1B, C: Can signinificance be estimated between Ctr and Casp3-sg?
2. 1D, E, G: Can additional apoptotic markers be shown in these Western Blots, for example Casp3 or PARP cleavage?
3. 1F: Why PARP1 is cleaved upon QVD addition?
4. Figure 4E: The corresponding Western Blots would be important: the analysis at the protein level of the expression of Puma, Noxa, and Bim.

In summary, this is a high-quality and aesthetically pleasing biochemical study with clear data, strong experimental design, and important implications for how apoptosis is rendered immunologically silent. With clarification or moderation of the claims regarding CASP3 specificity, the manuscript will make a valuable contribution to the field.

Referee #2:

Reviewer comments EMBOR-2025-63467V1

In their manuscript entitled "Caspase-3 cleaves CYLD to restrict Interferon Signaling during mitochondrial Apoptosis" (EMBOR-2025-63467V1) Macé et al. provide convincing evidence for the claims in the title and undoubtedly show that ABT-737&S63845 incubation of THP1 cells generates fragments that are clearly distinct from those produced by caspase-8 or MALT1. The experimental strategy is comprehensive and technically strong, combining pharmacological apoptosis induction (ABT-737/S63845), knock-down approaches, caspase inhibition, site-directed mutagenesis, qRT-PCR, and functional deubiquitination assays. The authors pinpoint Asp364 as cleavage site of Caspase-3 by site directed mutagenesis. The biochemical evidence for CYLD as a direct caspase-3 substrate is convincing.
However, the study remains largely descriptive with a significant lack of biological consequences. Key aspects concerning physiological relevance, downstream signaling effects, temporal dynamics, and model generalizability are insufficiently addressed and currently limit the conceptual impact.
In summary, the manuscript is well and comprehensively written and provides reliable biochemical evidence for caspase-3-mediated cleavage of CYLD at Asp354 during intrinsic apoptosis. To elevate the study beyond descriptive proteolysis, additional work addressing downstream functional consequences, temporal dynamics, and broader model validation is necessary.

Major points
a) The authors induce intrinsic apoptosis by simultaneous blocking anti-apoptotic proteins BCL-2, BCL-xL, BCL-w and MCL1 using ABT-737&S63845. It would be nice to have similar analyses using the classical cytochrome c/dATP-activation of cytoplasmic extracts. This system allows more direct manipulation (depletion by IP, addition of inhibitors) and thus is useful for mechanistic dissection.
b) While CYLD cleavage is convincingly demonstrated, the exploration of functional consequences is only scarcely touched. Given CYLD's role in inflammatory and stress-responsive signaling pathways, analysis of downstream target gene expression (beyond qRT-PCR) would substantially strengthen the mechanistic relevance.
c) Although inhibitor and mutagenesis data support caspase-3 involvement, validation in a genetically caspase-3-deficient system (e.g. MCF-7 cells, as described by Jänicke et al.) would provide decisive, orthogonal evidence for specificity. In this line, the results from experiments with recombinant proteins and tagged CYLD should be analyzed in western blots site-by-site with cellular extracts.
d) Temporal window. Most experiments are performed at a single early time point (1 h or 2 h). While sufficient to capture cleavage events, this narrow window precludes conclusions about sustained signaling or downstream effects. Inclusion of extended incubation times and presentation of time kinetic data (using lower concentration of inhibitors) is strongly recommended. The authors should also attempt to proof induction of apoptotic cells death in their experimental system more thoroughly. Presentation of bright field microscopy images might by an easy yet convincing way to proof apoptosis induction.
e) CellTiter-Glo measurements (ATP content) after 1 h are difficult to interpret. More direct evidence of apoptosis rather than reduced ATP is demanded (see point d)
f) The study relies predominantly on THP-1 cells, although HL-60, OCI-AML5, MOLM-13, and MOLM-15 are shown in Fig. 1F. Limited validation of additional experimental results in additional cell lines would increase confidence in the general applicability of the conclusions.
g) This reviewer would be interested in a direct comparison of results in Fig 1C and 4F.

Minor points
a) Annotation of experimental conditions. Incubation times and drug concentrations should be depicted directly in the graphs (e.g. axis labels or legends) rather than being accessible only via the Methods section.
b) Use and consistency of symbols (open vs. closed circles). The meaning of open and closed circles appears inconsistent between figures. Please double check use of these symbols explicitly in all figure legends (e.g. Fig. 1D/E) and ensure consistent usage throughout the manuscript.
c) Error bars are shown as SEM, which does not allow intuitive assessment of data dispersion. Please consider presenting SD instead, or alternatively, clearly indicate the number of biological replicates (n) directly in the figures or legends.

Referee #3:

This manuscript by Mace et al showed that in THP1 cells as well as in AML cell lines, activation of the intrinsic apoptotic pathway following inhibition of BCL2 family members using BH3 mimetics, resulted in the cleavage of CYLD at D364. This cleavage is mediated by CASP3. This novel cleavage of CYLD adds to previous observations that CYLD is cleaved by CASP8 at D215 and by MALT1 at R324. The authors also showed that one functional effect of CYLD cleavage by CASP3 is attenuation of the type I IFN pathway subsequent to mitochondria damage caused by mitochondria nuclei acid induced STING-IRF3 activation. CYLD has been shown to play a positive role in this pathway and cells expressing the cleavage resistant D364A variant of CYLD have greater IFNa/b signaling. The same mutation did not appear to impact apoptosis induced by the BH3 mimetics after 1 hour (Fig EV4A) but did enhance apoptosis at 24 h (Fig 4F). The authors attributed the effect at the 24 h timepoint to IFNa/b-mediated upregulation of BH3 family members. The biochemical analysis of the CASP3-CYLD interaction is quite convincing. The impact of this cleavage event on IFNa/b signaling and apoptosis with prolonged BH3 mimetic treatment is also convincing.
Major issues:
1. Authors used a combination of ABT-737 and S63845 to trigger intrinsic apoptosis and some of the analyses were done within an hour of treatment. This seems like a sledgehammer and some of the more subtle effects of CYLD deficiency (e.g., Fig 1C) or of the D364A mutation (Fig EV4A & 4F) might be lost. Have the authors examined the use of single BH3 mimetic? In addition to dose response effects, it would be nice to also conduct kinetic experiments to show the impact of CYLD deficiency and CYLD cleavage resistance on the readouts they examined.
2. Related to point 1 above, can CYLD cleavage also be observed with inducers of intrinsic apoptosis that are not BH3 mimetics? For example, DNA damage? Presumably this CASP3-mediated cleavage doesn't come into play during apoptosis induced by CASP8-activating treatments that leads to BID cleavage (e.g., FAS ligation) because the C-terminal CYLD fragment from CASP8 cleavage is already unstable, so it won't be available for CASP3 to act on. In other words, is the CASP3 cleavage only observed under conditions where CASP3 is activated without CASP8 being activated?
3. Is CASP7 also capable of cleaving CYLD at D364?
4. Does cleavage of CYLD at D364 have any impact on NF-kappaB signaling?

Minor issue:
1. Can the authors clarify what the two different shades of brown color for the D364A mutation (Fig 4D-F, EV4A,B & D) stand for? Are these two individual clones with the D364A mutation?

Nicolas Bidère
CANOPE Institute INSERM U1307/CNRS 6075/ UN
SOAP: Signaling in Oncogenesis, Angiogenesis, and Permeability
8 quai Moncousu BP 70721
44007 Nantes Cedex 1
France
Ph: 33 68 352 6986
Email: nicolas.bidere@inserm.fr

May 11[th], 2026

Dr Martina Rembold
Senior Editor, *EMBO Reports*

Re: EMBOR-2025-63467V1

Dear Dr Rembold,

Thank you very much for considering our manuscript for publication in *EMBO Reports*. We also wish to thank the three referees for their positive and insightful comments, which helped enhance our manuscript. All three Reviewers commanded the interest and quality of data, although suggesting some additional lines of experimentation. We have now had time to carry out substantial additional experimental work to address the reviewers' queries.

Please find below our point-by-point discussion in which we reiterate the reviewers' points and provide a rebuttal.

**Referee#1:**
This manuscript presents a beautiful and carefully executed biochemical study addressing an important question at the interface of apoptosis and inflammatory signaling. The work is technically strong and conceptually interesting, and the conclusions are largely well supported by the data.
A major strength of the study is the exceptional quality of the biochemical analyses. The western blots are very clear and convincing, and the biochemical experiments are thoughtfully designed and well controlled. In particular, the generation of CYLD mutants and the systematic analysis of their function in vitro represent a substantial experimental effort and are executed to a very high standard. The data are presented clearly, and the story is easy to follow. Overall, this is genuinely a very elegant and impressive piece of work. The identification of CYLD as a CASP3 substrate, cleaved at a site distinct from the previously described CASP8 site, is novel and potentially important. The use of knock-in cells expressing CASP3-resistant CYLD strengthens the physiological relevance of the findings and supports the conclusion that CYLD proteolysis contributes to limiting interferon signaling and cell death during apoptosis.
We would like to thank the Reviewer for the positive assessment of our manuscript and for his/her insightful suggestions to enhance its quality.

That said, I remain somewhat puzzled by the assertion that the identified cleavage site represents a de facto CASP3-specific site. As caspases share overlapping substrate specificities and many substrates can be cleaved by multiple caspases under certain conditions, it is difficult to exclude potential contributions from other caspases based primarily on in vitro experiments. While the in vitro assays are informative and suggest differences in cleavage efficiency, they may not fully capture the complexity of caspase activity in cells. A more cautious discussion of caspase specificity, or additional experimental support, would strengthen this aspect of the manuscript. There is a last paragraph in the discussion,

Nicolas Bidère
CANOPE Institute INSERM U1307/CNRS 6075/ UN
SOAP: Signaling in Oncogenesis, Angiogenesis, and Permeability
8 quai Moncousu BP 70721
44007 Nantes Cedex 1
France
Ph: 33 68 352 6986
Email: nicolas.bidere@inserm.fr

but I think more discussion ( or experimental data if available) of caspase-8 specificity versus caspase-3 is required. I think the link to caspase-8- activation platforms has to be made in this context and discussed accordingly.

These insightful points were also raised by Reviewer#3 (please see his/her point #3). We agree that additional CASPs, particularly CASP7, could potentially contribute to CYLD cleavage at D364, given their overlapping substrate specificities (Thornberry *et al*, 1997). Our additional experiments show that CASP7 efficiently cleaves CYLD *in vitro*, generating a product similar to that produced by CASP3 (new Fig. EV3G). Moreover, CASP7-mediated cleavage of CYLD was abolished when the CYLD D364A mutant was used, indicating that both CASP3 and CASP7 cleave CYLD at the same cleavage site (new Fig. EV3H). Furthermore, active CASP7 was detected in lysates from cells treated with BH3 mimetics, and its activation was only partly dependent on CASP3 (new Fig. EV3I). Despite this, CYLD cleavage was prevented in CASP3 knockout cells, as well as in MCF7 cells, which naturally lack CASP3 (Fig. 2B, EV3I and Appendix Fig. S1A). Collectively, these findings suggest that CYLD is predominantly cleaved by CASP3 in cells undergoing mitochondrial apoptosis. These results have now been incorporated and further discussed in the revised manuscript (please see page 4, lines 26-36 and page 6, lines 3-9).

As highlighted by the Reviewer, CASP activation often involves the assembly of dedicated supramolecular complexes, raising the possibility that spatial compartmentalization dictates the differential cleavage of CYLD. For example, CYLD is dynamically recruited to the TNF receptor signaling complexes in close proximity to CASP8 following TNFα stimulation (Draber *et al*, 2015; O'Donnell *et al*, 2011; Micheau & Tschopp, 2003). Additional work is therefore required to elucidate whether CYLD associates with CASP3-containing signaling platform during mitochondrial apoptosis. Intriguingly, a pool of CYLD localizes to centriolar satellites, and we have previously shown that several components of these proteinaceous structures are cleaved by CASP3 downstream of MOMP (Douanne *et al*, 2019; Renaud *et al*, 2023). This observation suggests a potential spatial node for triggering CASP3-dependent proteolysis. This perspective has now been incorporated in the Discussion section of the revised manuscript (please see page 6, lines 16-26).

<u>More comments:</u>
1. 1B, C: Can significance be estimated between Ctr and Casp3-sg?
The significance has been added to the figures of the revised manuscript.

2. 1D, E, G: Can additional apoptotic markers be shown in these Western Blots, for example Casp3 or PARP cleavage?
At the Reviewer's request, immunoblotting analyses of PARP and CASP3 were included in the panels D, E, and G of the new Figure 1. As pointed out in our response to Reviewer#2 (please see his/her point c and d), we also assessed other hallmarks of apoptosis (morphological changes, CASP3/7 activation with CASP Glo, and loss of phosphatidylserine asymmetry).

3. 1F: Why PARP1 is cleaved upon QVD addition?
We agree that this experiment was confusing. This set of data was, therefore, substituted with a repeat experiment, which shows that CASP inhibition prevents PARP cleavage.

4. Figure 4E: The corresponding Western Blots would be important: the analysis at the protein level of the expression of Puma, Noxa, and Bim.
We concur with the Reviewer regarding the importance of these experiments. Despite several attempts, we failed to detect a robust induction of Puma, Noxa, and Bim at the protein level. We think that the expression of these BH3-only proteins precipitates cell destruction, thereby limiting their detectability. Supporting this idea, we observed that interfering with CASPs with QVD-OPh, as shown by Diepstraten

Nicolas Bidère
CANOPE Institute INSERM U1307/CNRS 6075/ UN
SOAP: Signaling in Oncogenesis, Angiogenesis, and Permeability
8 quai Moncousu BP 70721
44007 Nantes Cedex 1
France
Ph: 33 68 352 6986
Email: nicolas.bidere@inserm.fr

and colleagues (Diepstraten *et al*, 2024), allows detecting the upregulation of Noxa (data not presented). However, this treatment precludes assessment of the effect of CASP3-mediated cleavage of CYLD.
Prompted by the Reviewers, we conducted additional experiments to gain further insights into the IRF3-IFNβ pathway and document its role during mitochondrial apoptosis. We found that cells expressing CYLD D364A displayed enhanced activation of IRF3 and of the downstream ISG IFIT1 in response to escalating doses of BH3 mimetics (new Fig. 4E and EV4D). This was accompanied by heightened apoptotic hallmarks, including CASP3 activation, PARP cleavage, and cell death (new Fig. EV5A-C). Of note, inhibition of cGAS with RU.521 partly normalizes this enhanced cell death (new Fig. 4I).

In summary, this is a high-quality and aesthetically pleasing biochemical study with clear data, strong experimental design, and important implications for how apoptosis is rendered immunologically silent. With clarification or moderation of the claims regarding CASP3 specificity, the manuscript will make a valuable contribution to the field.

**Referee #2:**
In their manuscript entitled "Caspase-3 cleaves CYLD to restrict Interferon Signaling during mitochondrial Apoptosis" (EMBOR-2025-63467V1), Macé et al. provide convincing evidence for the claims in the title and undoubtedly show that ABT-737&S63845 incubation of THP1 cells generates fragments that are clearly distinct from those produced by caspase-8 or MALT1. The experimental strategy is comprehensive and technically strong, combining pharmacological apoptosis induction (ABT-737/S63845), knock-down approaches, caspase inhibition, site-directed mutagenesis, qRT-PCR, and functional deubiquitination assays. The authors pinpoint Asp364 as cleavage site of Caspase-3 by site directed mutagenesis. The biochemical evidence for CYLD as a direct caspase-3 substrate is convincing.
However, the study remains largely descriptive with a significant lack of biological consequences. Key aspects concerning physiological relevance, downstream signaling effects, temporal dynamics, and model generalizability are insufficiently addressed and currently limit the conceptual impact.
In summary, the manuscript is well and comprehensively written and provides reliable biochemical evidence for caspase-3-mediated cleavage of CYLD at Asp354 during intrinsic apoptosis. To elevate the study beyond descriptive proteolysis, additional work addressing downstream functional consequences, temporal dynamics, and broader model validation is necessary.
We thank the Reviewer for the positive assessment of our work and for his/her constructive remarks.

Major points

a) The authors induce intrinsic apoptosis by simultaneous blocking anti-apoptotic proteins BCL-2, BCL-xL, BCL-w and MCL1 using ABT-737&S63845. It would be nice to have similar analyses using the classical cytochrome c/dATP-activation of cytoplasmic extracts. This system allows more direct manipulation (depletion by IP, addition of inhibitors) and thus is useful for mechanistic dissection.
This is an insightful suggestion we tested experimentally. To this end, post-nuclear extracts from THP-1 and MCF7 cells were prepared and incubated with cytochrome c and dATP, as previously described (Slee *et al*, 1999, 2001). Consistent with previous studies, the addition of cyt.c/dATP induced CASP3 proteolysis in extracts from TPH-1 cells but not from MCF7 cells, which lack endogenous CASP3. In these conditions, CYLD was only cleaved in THP-1 extracts. Moreover, CYLD cleavage was efficiently prevented by pretreating cell extracts with the pan-CASP inhibitor Q-VD-OPh. Combined with our *in vitro* and *in cellulo* data, these results, presented in the new Fig. 1F and 2D, reinforce the idea that CASP3 cleaves CYLD.

Nicolas Bidère
CANOPE Institute INSERM U1307/CNRS 6075/ UN
SOAP: Signaling in Oncogenesis, Angiogenesis, and Permeability
8 quai Moncousu BP 70721
44007 Nantes Cedex 1
France
Ph: 33 68 352 6986
Email: nicolas.bidere@inserm.fr

b) While CYLD cleavage is convincingly demonstrated, the exploration of functional consequences is only scarcely touched. Given CYLD's role in inflammatory and stress-responsive signaling pathways, analysis of downstream target gene expression (beyond qRT-PCR) would substantially strengthen the mechanistic relevance.

To further strengthen the role of CYLD cleavage by CASP3 in inflammation and cell death during mitochondrial apoptosis beyond mRNA induction, several lines of experimentation were carried out. As discussed above (please see the response to Reviewer#1, his/her point 4), we encountered technical limitations in assessing protein expression of proapoptotic BCL2 family members, as cells undergo simultaneous cell destruction (Diepstraten *et al*, 2024). Nevertheless, we have conducted additional experiments to gain further insights into the IRF3-IFNβ pathway and document its role during mitochondrial apoptosis. We now report that cells expressing CYLD D364A exhibit enhanced IRF3 phosphorylation and increased IFIT1 protein expression (new Fig. 4E and EV4D). Consistent with a role of the cGAS-STING pathway (Rongvaux *et al*, 2014; White *et al*, 2014), these events were attenuated in cells treated with the cGAS inhibitor RU.521. In addition, time-course experiments confirmed the increase in cell death (CASP3 activation, PARP cleavage, and cell death, new Fig. EV5A-C). Finally, RU.521 treatment partially normalizes this enhanced cell death (new Fig. 4I). These results have been incorporated in the revised version of the manuscript.

c) Although inhibitor and mutagenesis data support caspase-3 involvement, validation in a genetically caspase-3-deficient system (e.g. MCF-7 cells, as described by Jänicke et al.) would provide decisive, orthogonal evidence for specificity. In this line, the results from experiments with recombinant proteins and tagged CYLD should be analyzed in western blots site-by-site with cellular extracts.

Following the Reviewer's suggestion, experiments with MCF7, which lack the expression of CASP3, were conducted. We found no cleavage of CYLD in lysates from MCF7 treated for 3h with Raptinal (10 μM) or BH3 mimetics (10 μM) (new Appendix Fig. S1A). Similar results were obtained with THP-1 and MDA-MB-231 cells knockout for CASP3 (Fig.2B and 2C). Moreover, the addition of cytochrome c and dATP to MCF7 cellular extracts failed to drive CYLD proteolysis, reinforcing the idea that CASP3 is central to CYLD cleavage (new Fig. 1F and 2D).

At the Reviewer's request, we showed that the CYLD COOH-terminal fragment obtained *in vitro* with recombinant proteins matched the one seen with experiments performed *in cellulo* (new Fig. EV3C). A graph summarizing the observed molecular weights is also shown in the new Fig. EV3D.

d) Temporal window. Most experiments are performed at a single early time point (1 h or 2 h). While sufficient to capture cleavage events, this narrow window precludes conclusions about sustained signaling or downstream effects. Inclusion of extended incubation times and presentation of time kinetic data (using lower concentration of inhibitors) is strongly recommended. The authors should also attempt to proof induction of apoptotic cells death in their experimental system more thoroughly. Presentation of bright field microscopy images might by an easy yet convincing way to proof apoptosis induction.

These important points were also raised by Reviewer #3 (his/her point#1) and echo, to some extent, the comment from Reviewer#1 (see his/her point#2). We agree that, in our initial submission, we did not assess the extent to which CellTiter Glo assays correlate with apoptosis in our model. Prompted by the Reviewers, we measured different hallmarks of apoptosis: morphological changes (brightfield microscopy), activation of CASPs (CASP3/7 Glo, biochemistry for CASP3 activation), and downstream loss of phosphatidylserine asymmetry (Annexin-V staining). As shown in the new Fig. EV1A-E, the drop in ATP measured with CellTiter Glo closely coincides with both morphological and biochemical features

Nicolas Bidère
CANOPE Institute INSERM U1307/CNRS 6075/ UN
SOAP: Signaling in Oncogenesis, Angiogenesis, and Permeability
8 quai Moncousu BP 70721
44007 Nantes Cedex 1
France
Ph: 33 68 352 6986
Email: nicolas.bidere@inserm.fr

of apoptosis. These data support the use of CellTiter Glo as a readout for apoptosis induction in our system.
At the Reviewer's request, we performed kinetic experiments using lower concentrations of BH3 mimetics. The enhanced cell death initially observed in CYLD KI cells overnight was also observed at earlier time points (essentially at 4 and 6 hours post-stimulation) and was associated with increased activation of CASP3/7. These results are presented in the new Fig. EV5B.

e) CellTiter-Glo measurements (ATP content) after 1 h are difficult to interpret. More direct evidence of apoptosis rather than reduced ATP is demanded (see point d)
As discussed above, we carried out complementary CASP3/7 Glo assays, brightfield examination of cells, and biochemical analyses for hallmarks of apoptosis. The data show that the drop in CellTiter Glo we observe correlates with the execution of cell death. These results have been included in the revised version of the manuscript (new Fig. EV1A-E). Nevertheless, to avoid confusion, we also indicated "metabolic cell viability assays" when referring to experiments using CellTiter Glo.

f) The study relies predominantly on THP-1 cells, although HL-60, OCI-AML5, MOLM-13, and MOLM-15 are shown in Fig. 1F. Limited validation of additional experimental results in additional cell lines would increase confidence in the general applicability of the conclusions.
This is an insightful suggestion. We have tried to engineer additional cellular models to express CYLD D364A and managed to obtain two clones of HL-60 harboring a heterozygous mutation, while being unsuccessful with OCI-AML5 (new Fig. EV3F). Using these cells, we confirmed the upregulation of inflammatory markers in response to BH3 mimetics (please see the new Fig. EV4C). Of note, we did not observe enhanced cell death, which can be explained by the remaining WT CYLD or the high sensitivity of HL-60 to BH3 mimetics.

g) This reviewer would be interested in a direct comparison of results in Fig 1C and 4F. The experiment, which shows a partial reduction in cell death in the absence of CYLD, has been performed and is now presented in the new Fig.EV1F.

Minor points

a) Annotation of experimental conditions. Incubation times and drug concentrations should be depicted directly in the graphs (e.g. axis labels or legends) rather than being accessible only via the Methods section.
Following the Reviewer's suggestion, we tried to improve the clarity of the figures and their legends.

b) Use and consistency of symbols (open vs. closed circles). The meaning of open and closed circles appears inconsistent between figures. Please double check use of these symbols explicitly in all figure legends (e.g. Fig. 1D/E) and ensure consistent usage throughout the manuscript.
We have amended the figures and their legends to enhance clarity.

c) Error bars are shown as SEM, which does not allow intuitive assessment of data dispersion. Please consider presenting SD instead, or alternatively, clearly indicate the number of biological replicates (n) directly in the figures or legends.
The number of biological replicates is now indicated in the figure legends.

**Referee #3:**

The biochemical analysis of the CASP3-CYLD interaction is quite convincing. The impact of this cleavage event on IFNa/b signaling and apoptosis with prolonged BH3 mimetic treatment is also

Nicolas Bidère
CANOPE Institute INSERM U1307/CNRS 6075/ UN
SOAP: Signaling in Oncogenesis, Angiogenesis, and Permeability
8 quai Moncousu BP 70721
44007 Nantes Cedex 1
France
Ph: 33 68 352 6986
Email: nicolas.bidere@inserm.fr

convincing.
We thank the Reviewer for the positive evaluation of our manuscript and for the insightful suggestions that helped improve its quality.

Major issues:
1. Authors used a combination of ABT-737 and S63845 to trigger intrinsic apoptosis and some of the analyses were done within an hour of treatment. This seems like a sledgehammer and some of the more subtle effects of CYLD deficiency (e.g., Fig 1C) or of the D364A mutation (Fig EV4A & 4F) might be lost. Have the authors examined the use of single BH3 mimetic? In addition to dose response effects, it would be nice to also conduct kinetic experiments to show the impact of CYLD deficiency and CYLD cleavage resistance on the readouts they examined.
These insightful remarks were raised by Reviewer#2 (his/her point #e and #d) and Reviewer#1 (his/her point#2). In our initial submission, we used a combination of BH3 mimetics (high concentrations for 1-2h or lower concentrations overnight). Following the Reviewers' suggestions, we performed additional titration and kinetic experiments. These analyses confirmed the impact of the CYLD D364A mutation at earlier time points and at lower concentrations, both in terms of cell survival and CASP activation (new Fig. 4G, 4H, and EV5B). Consistently, the effect of CYLD deficiency was also seen with lower concentrations of BH3 mimetics (new Fig. EV1F).
As pointed out, BH3 mimetics were initially used in combination throughout the manuscript. At the Reviewer's suggestion, each BH3 mimetic was used alone. Supporting previous works (Greaves *et al*, 2019), we observed that THP-1 cells were more sensitive to MCL1 inhibition by S63845 than BCL-2/BCL-XL/BCL-w inhibition with ABT-737. Notably, however, cells expressing D364A displayed enhanced ABT-737- and not S63845-mediated cell death. These new results are included in the revised version of the manuscript (new Fig. EV5C).

2. Related to point 1 above, can CYLD cleavage also be observed with inducers of intrinsic apoptosis that are not BH3 mimetics? For example, DNA damage? Presumably this CASP3-mediated cleavage doesn't come into play during apoptosis induced by CASP8-activating treatments that leads to BID cleavage (e.g., FAS ligation) because the C-terminal CYLD fragment from CASP8 cleavage is already unstable, so it won't be available for CASP3 to act on. In other words, is the CASP3 cleavage only observed under conditions where CASP3 is activated without CASP8 being activated?
This is an insightful remark. We often observe concomitant activation of CASP3 and CASP8 (see our figures). However, as pointed out by the Reviewer, testing whether CYLD can be subsequently cleaved by CASP8 and CASP3 may prove difficult.
Following the Reviewer's suggestion, we also tested additional stimuli. We now show that CYLD cleavage can also be observed in cells treated with the topoisomerase II inhibitor etoposide or the PKC inhibitor staurosporine (new Fig. EV1G and EV1H). This nicely aligns with the data obtained with Raptinal, which activates CASP3 independently of BCL2 family members.

3. Is CASP7 also capable of cleaving CYLD at D364?
This is a very intriguing question (also raised by Reviewer#1, his/her general comment), which we thought to address experimentally. CASP3 and CASP7 are often considered as effector CASPs with largely overlapping substrate repertoires. However, accumulating evidence indicates that they are functionally distinct proteases with CASP3 being the major effector protease and CASP7 displaying a more selective set of substrates (Walsh *et al*, 2008). *In vitro*, we observed that recombinant human CASP7 cleaves CYLD, generating a predominant cleavage product comparable to that produced by rhCASP3 (new Fig. EV3G). This processing was abolished when the CYLD D364A mutant was used, indicating that both CASP3 and CASP7 cleave CYLD at the same site (new Fig. EV3H). Notably, active

CANOPE Institute
CANCER & IMMUNOLOGY
Expanding research to drive connection to living systems

Soap
Signaling in Oncogenesis
Angiogenesis & Permeability

Nicolas Bidère
CANOPE Institute INSERM U1307/CNRS 6075/ UN
SOAP: Signaling in Oncogenesis, Angiogenesis, and Permeability
8 quai Moncousu BP 70721
44007 Nantes Cedex 1
France
Ph: 33 68 352 6986
Email: nicolas.bidere@inserm.fr

CASP7 was detected in lysates from cells treated with BH3 mimetics, and its activation was only partly dependent on CASP3 (new Fig. EV3I). Despite this, CYLD cleavage was prevented in CASP3 knockout cells, as well as in MCF7 cells, which naturally lack CASP3 (new Appendix Fig. S1A). Taken together, these findings suggest that CYLD is predominantly cleaved by CASP3 in cells undergoing mitochondrial apoptosis. These points have now been included in both the results and discussion sections of the revised manuscript.

4. Does cleavage of CYLD at D364 have any impact on NF-kappaB signaling?
This is an insightful remark. Consistent with previous findings (Vringer *et al*, 2024), mitochondrial outer membrane permeabilization also triggered NF-κB activation in THP-1 cells, which was further increased in cells expressing CYLD D364A as assessed by immunoblotting (new Fig. EV4E).

Minor issue:
1. Can the authors clarify what the two different shades of brown color for the D364A mutation (Fig 4D-F, EV4A,B & D) stand for? Are these two individual clones with the D364A mutation?
We agree that our labelling for the two individual clones was rather confusing and opted for a clearer color code.

We thank you again for your hard work on our manuscript and hope that it will be suitable for publication in *EMBO Reports* with these additional experiments.

Nicolas Bidère

Nicolas Bidère
CANOPE Institute INSERM U1307/CNRS 6075/ UN
SOAP: Signaling in Oncogenesis, Angiogenesis, and Permeability
8 quai Moncousu BP 70721
44007 Nantes Cedex 1
France
Ph: 33 68 352 6986
Email: nicolas.bidere@inserm.fr

## References

Diepstraten ST, Yuan Y, La Marca JE, Young S, Chang C, Whelan L, Ross AM, Fischer KC, Pomilio G, Morris R, *et al* (2024) Putting the STING back into BH3-mimetic drugs for TP53-mutant blood cancers. *Cancer Cell* 42: 850-868.e9

Douanne T, André-Grégoire G, Thys A, Trillet K, Gavard J & Bidère N (2019) CYLD Regulates Centriolar Satellites Proteostasis by Counteracting the E3 Ligase MIB1. *Cell Reports* 27: 1657-1665.e4

Draber P, Kupka S, Reichert M, Draberova H, Lafont E, de Miguel D, Spilgies L, Surinova S, Taraborrelli L, Hartwig T, *et al* (2015) LUBAC-Recruited CYLD and A20 Regulate Gene Activation and Cell Death by Exerting Opposing Effects on Linear Ubiquitin in Signaling Complexes. *Cell Rep* 13: 2258–2272

Greaves G, Milani M, Butterworth M, Carter RJ, Byrne DP, Eyers PA, Luo X, Cohen GM & Varadarajan S (2019) BH3-only proteins are dispensable for apoptosis induced by pharmacological inhibition of both MCL-1 and BCL-XL. *Cell Death Differ* 26: 1037–1047

Micheau O & Tschopp J (2003) Induction of TNF receptor I-mediated apoptosis via two sequential signaling complexes. *Cell* 114: 181–190

O'Donnell MA, Perez-Jimenez E, Oberst A, Ng A, Massoumi R, Xavier R, Green DR & Ting AT (2011) Caspase 8 inhibits programmed necrosis by processing CYLD. *Nature Cell Biology* 13: 1437–1442

Renaud CCN, Trillet K, Jardine J, Merlet L, Renoult O, Laurent—Blond M, Catinaud Z, Pecqueur C, Gavard J & Bidère N (2023) The centrosomal protein 131 participates in the regulation of mitochondrial apoptosis. *Commun Biol* 6: 1271

Rongvaux A, Jackson R, Harman CCD, Li T, West AP, de Zoete MR, Wu Y, Yordy B, Lakhani SA, Kuan C-Y, *et al* (2014) Apoptotic Caspases Prevent the Induction of Type I Interferons by Mitochondrial DNA. *Cell* 159: 1563–1577

Slee EA, Adrain C & Martin SJ (2001) Executioner Caspase-3, -6, and -7 Perform Distinct, Non-redundant Roles during the Demolition Phase of Apoptosis. *Journal of Biological Chemistry* 276: 7320–7326

Slee EA, Harte MT, Kluck RM, Wolf BB, Casiano CA, Newmeyer DD, Wang H-G, Reed JC, Nicholson DW, Alnemri ES, *et al* (1999) Ordering the Cytochrome c–initiated Caspase Cascade: Hierarchical Activation of Caspases-2, -3, -6, -7, -8, and -10 in a Caspase-9–dependent Manner. *The Journal of Cell Biology* 144: 281–292

Thornberry NA, Rano TA, Peterson EP, Rasper DM, Timkey T, Garcia-Calvo M, Houtzager VM, Nordstrom PA, Roy S, Vaillancourt JP, *et al* (1997) A combinatorial approach defines specificities of members of the caspase family and granzyme B. Functional relationships established for key mediators of apoptosis. *J Biol Chem* 272: 17907–17911

Vringer E, Heilig R, Riley JS, Black A, Cloix C, Skalka G, Montes-Gómez AE, Aguado A, Lilla S, Walczak H, *et al* (2024) Mitochondrial outer membrane integrity regulates a ubiquitin-dependent and NF-κB-mediated inflammatory response. *EMBO J* 43: 904–930

Walsh JG, Cullen SP, Sheridan C, Lüthi AU, Gerner C & Martin SJ (2008) Executioner caspase-3 and caspase-7 are functionally distinct proteases. *Proc Natl Acad Sci U S A* 105: 12815–12819

White MJ, McArthur K, Metcalf D, Lane RM, Cambier JC, Herold MJ, van Delft MF, Bedoui S, Lessene G, Ritchie ME, *et al* (2014) Apoptotic caspases suppress mtDNA-induced STING-mediated type I IFN production. *Cell* 159: 1549–1562

Dear Dr. Bidere

Thank you for the submission of your revised manuscript to EMBO reports. We have now received the full set of referee reports that is copied below.

As you will see, all referees are very positive about the study and request only minor changes to clarify text and figures.

From the editorial side, there are also a few things that we need before we can proceed with the official acceptance of your study.

- There is an author name inconsistency: Tiphaine Douanne in the manuscript vs. Douanne Tiphaine in the system. The name in the system needs to be provided in the same format - first name last name.

- Please rename the Ethics Declaration to Disclosure and Competing Interests Statement and place it after the Acknowledgments.

- Regarding the Author Contributions, we use CRediT to specify the contributions of each author in the journal submission system. CRediT replaces the author contribution section, which therefore needs to be removed from the manuscript text. You can use the free text box in our system if you wish to provide more detailed descriptions. See also guide to authors https://www.embopress.org/page/journal/14693178/authorguide#authorshipguidelines.

- Please convert Figure 1 to portrait orientation.

- The Appendix file has only one figure. Could this be provided as one of the Expanded View figures so that a separate Appendix is not required? If you choose to keep it we need page numbers, even if it is only one page.

- Reagents and Tools table: the separate file is sufficient. It will be typeset into the article, therefore, please remove the table from the manuscript text. Please don't forget to remove the "Instructions" paragraph from the Reagent Table file you uploaded.

- The CYLD blot shown in Figure 2B looks very similar to the one in Figure EV3 I. Please carefully check these panels also whether the reuse is justified and the provided GAPDH controls are the correct ones. If all these blots are indeed from the same experiment and the appropriate controls are shown, then please clearly indicate the reuse in both figure legends.

- The resolution of the submitted figures is too low for blot and microscopy images. This reduction in resolution is commonly caused by converting original 16-bit TIFF files to RGB format for publication. While this is not inherently problematic, it can raise concerns about image integrity for critical readers.

To avoid any misunderstanding and to meet EMBO Press standards, we kindly ask that you:

* Resubmit the complete figure set at the captured original data resolution.

- Our data editors checked your figure legends for completeness and ask you to address the following points:
- Please note that the exact p values are not provided in the legends of 1B,C; 4B-D,F-I; EV-1B,E,F; EV-2B; EV-4B,C,F; EV-5B-D.
- Please note that information related to n is missing in the legends 2F.

- Please change the size of the synopsis image to 550 x 300 pixels (currently it has 385 px height). You might also want to consider increasing text size a bit.

We look forward to seeing a final version of your manuscript as soon as possible.

With kind regards,

Martina Rembold, PhD
Senior Editor
EMBO reports

==================

Referee #1:

The authors performed revisions and fully addressed my concerns.

Referee #2:

Reviewer comments revised version

Reviewer Comments
This reviewer thanks the authors for addressing all major concerns raised in a satisfactory manner. The additional data and revisions further strengthen the conclusion that CYLD undergoes a distinct caspase-3-dependent cleavage during intrinsic apoptosis. The experiments clearly provide convincing evidence for the proposed cleavage mechanism and its consequences for CYLD deubiquitinase activity.
Some minor issues should be addressed by the authors:
Fig. 1A and Fig. 4A: Please align the graphical elements and standardize box widths to improve visual consistency.
Fig. 1B and similar figures: Please indicate the number of biological replicates (n). Furthermore, the designation "% of 0" may be misleading. This reviewer suggests labelling the data as "% of Ctrl".
Fig. 1D and Western blots in general: Molecular weight markers are indicated as "Mr(K)". This reviewer is not familiar with this notation and assumes it refers to kDa. Please clarify.
Fig. 3D: Caspase cleavage motifs comprise four amino acids (P4-P1). To better visualize the cleavage site context, please extend the displayed amino acid sequence at least to residue 342.
Fig. 4B and qRT-PCR figures in general: This reviewer suggests using "Relative RNA Expression Level" as the y-axis label.
Fig. 4G and similar figures: This reviewer suggests the label "CASP3/7 Activity (fold Ctrl)".
Fig. 1D, Fig. 2G, Fig. 3G, Fig. EV1D, Fig. EV1E, Fig. EV2D, and similar panels: Please include the leading zero for decimal values (e.g. 0.5 instead of .5) throughout the manuscript for consistency.
Fig. EV5B: Please verify concentration annotations for consistency. For example, concentrations are reported as 88 nM in the CASP3/7 activity assay and 88.9 nM in the corresponding cell survival experiment.
P12L30: please correct 20.106
P12L32: please use subscript in MgCl2

Referee #3:

The authors have adequately addressed the critiques raised previously by this reviewer. There are a couple of small errors that the authors might want to check on.
1. The closed and open circles in Fig. EV3H might be incorrectly labeled in the right two lanes.
2. Page 5, line 27. Instead of "CYLD cleavage allows timely apoptosis", it would be more appropriate to say 'CYLD cleavage attenuates apoptosis." This is because the cleavage-resistant CYLD-D364A cells were more sensitive to cell death (Fig. 4H, I, EV5A, 5B).
3. Page 19, line 12. The legend to EV4E should indicate that the cells were treated with A/S for 1 and 2 h since the figure indicates 0, 1 and 2 h.

CANOPE Institute
CANCER & IMMUNOLOGY
Expanding research to drive connection to living systems

Soap
Signaling in Oncogenesis
Angiogenesis & Permeability

Nicolas Bidère
CANOPE Institute INSERM U1307/CNRS 6075/ UN
SOAP: Signaling in Oncogenesis, Angiogenesis, and Permeability
8 quai Moncousu BP 70721
44007 Nantes Cedex 1
France
Ph: 33 68 352 6986
Email: nicolas.bidere@inserm.fr

June 30th, 2026

Dr Martina Rembold
Senior Editor, *EMBO Reports*

Re: EMBOR-2025-63467V2

Dear Dr Rembold,

We thank you for the positive assessment of our revised manuscript and are delighted that it has been accepted in principle for publication in *EMBO Reports*. We appreciate the time and effort invested by the editors and reviewers throughout the evaluation process.
We have carefully addressed all points raised in your message and revised the manuscript accordingly. A detailed response to each point is provided below.

**Editor:**
1. There is an author name inconsistency: Tiphaine Douanne in the manuscript vs. Douanne Tiphaine in the system. The name in the system needs to be provided in the same format - first name last name.
This issue has been corrected.

2. Please rename the Ethics Declaration to Disclosure and Competing Interests Statement and place it after the Acknowledgments.
This has been done.

3. Regarding the Author Contributions, we use CRediT to specify the contributions of each author in the journal submission system. CRediT replaces the author contribution section, which therefore needs to be removed from the manuscript text. You can use the free text box in our system if you wish to provide more detailed descriptions. See also guide to
authors https://www.embopress.org/page/journal/14693178/authorguide#authorshipguidelines.
As requested, we have removed this section from the manuscript.

4. Please convert Figure 1 to portrait orientation.
Fig.1 has been reformatted in portrait orientation.

5. The Appendix file has only one figure. Could this be provided as one of the Expanded View figures so that a separate Appendix is not required? If you choose to keep it we need page numbers, even if it is only one page.
The figures from the Appendix file have been incorporated into the new Fig. EV3.

6. Reagents and Tools table: the separate file is sufficient. It will be typeset into the article, therefore, please remove the table from the manuscript text. Please don't forget to remove the "Instructions" paragraph from the Reagent Table file you uploaded.
The table has been removed.

7. The CYLD blot shown in Figure 2B looks very similar to the one in Figure EV3 I. Please carefully check these panels also whether the reuse is justified and the provided GAPDH controls are the correct ones. If all these blots are indeed from the same experiment and the appropriate controls are shown, then please clearly indicate the reuse in both figure legends.
The CYLD blot presented in Fig. EV3I (new Fig. EV3K) is different from the blot shown in Fig. 2B. Nevertheless, to avoid any potential confusion, we have replaced the panel with data from a third repeat experiment in this revised manuscript.

8. The resolution of the submitted figures is too low for blot and microscopy images. This reduction in resolution is commonly caused by converting original 16-bit TIFF files to RGB format for publication. While this is not inherently problematic, it can raise concerns about image integrity for critical readers.
We have increased the resolution of the images.

9. To avoid any misunderstanding and to meet EMBO Press standards, we kindly ask that you:
* Resubmit the complete figure set at the captured original data resolution.

Nicolas Bidère
CANOPE Institute INSERM U1307/CNRS 6075/ UN
SOAP: Signaling in Oncogenesis, Angiogenesis, and Permeability
8 quai Moncousu BP 70721
44007 Nantes Cedex 1
France
Ph: 33 68 352 6986
Email: nicolas.bidere@inserm.fr

- Our data editors checked your figure legends for completeness and ask you to address the following points:
- Please note that the exact p values are not provided in the legends of 1B,C; 4B-D,F-I; EV-1B,E,F; EV-2B; EV-4B,C,F; EV-5B-D.
The p-values have now been included in each figure legend.

- Please note that information related to n is missing in the legends 2F.
Information (n=4) has been added in the legend.

- Please change the size of the synopsis image to 550 x 300 pixels (currently it has 385 px height). You might also want to consider increasing text size a bit.
The image has been resized and the text size has been increased to improve readability.

**Referee #2:**
1. Fig. 1A and Fig. 4A: Please align the graphical elements and standardize box widths to improve visual consistency.
The graphical elements have been aligned and box widths standardized.

2. Fig. 1B and similar figures: Please indicate the number of biological replicates (n). Furthermore, the designation "% of 0" may be misleading. This reviewer suggests labelling the data as "% of Ctrl".
The number of biological replicates now appears in the legends. In addition, we replaced "% of 0" with "% of Untr."

3. Fig. 1D and Western blots in general: Molecular weight markers are indicated as "Mr(K)". This reviewer is not familiar with this notation and assumes it refers to kDa. Please clarify.
The number of biological replicates now appears in the legends. Mr (K) refers to kDa. To avoid ambiguity, this has been removed from the figures.

4. Fig. 3D: Caspase cleavage motifs comprise four amino acids (P4-P1). To better visualize the cleavage site context, please extend the displayed amino acid sequence at least to residue 342.
This is a good point. We have extended the sequence to include residue 342.

5. Fig. 4B and qRT-PCR figures in general: This reviewer suggests using "Relative RNA Expression Level" as the y-axis label.
At the Reviewer's suggestion, the labelling of the y-axis was changed.

6. Fig. 4G and similar figures: This reviewer suggests the label "CASP3/7 Activity (fold Ctrl)".
At the Reviewer's suggestion, the labelling of the y-axis was changed to "CASP3/7 Activity (Fold of Untr.)".

7. Fig. 1D, Fig. 2G, Fig. 3G, Fig. EV1D, Fig. EV1E, Fig. EV2D, and similar panels: Please include the leading zero for decimal values (e.g. 0.5 instead of .5) throughout the manuscript for consistency.
The formatting has been corrected.

8. Fig. EV5B: Please verify concentration annotations for consistency. For example, concentrations are reported as 88 nM in the CASP3/7 activity assay and 88.9 nM in the corresponding cell survival experiment.
We thank the Reviewer for identifying this discrepancy. The concentration has been corrected to to 88.9 nM.

9. P12L30: please correct 20.106
P12L32: please use subscript in MgCl2
Changes were made.

**Referee #3:**
1. The closed and open circles in Fig. EV3H might be incorrectly labeled in the right two lanes.
We thank the Reviewer for noticing this error. The labelling has been corrected.

2. Page 5, line 27. Instead of "CYLD cleavage allows timely apoptosis", it would be more appropriate to say 'CYLD cleavage attenuates apoptosis." This is because the cleavage-resistant CYLD-D364A cells were more sensitive to cell death (Fig. 4H, I, EV5A, 5B).
We agree and have amended the text as suggested.

Nicolas Bidère
CANOPE Institute INSERM U1307/CNRS 6075/ UN
SOAP: Signaling in Oncogenesis, Angiogenesis, and Permeability
8 quai Moncousu BP 70721
44007 Nantes Cedex 1
France
Ph: 33 68 352 6986
Email: nicolas.bidere@inserm.fr

3. Page 19, line 12. The legend to EV4E should indicate that the cells were treated with A/S for 1 and 2 h since the figure indicates 0, 1 and 2 h.
Changes have been made.

We thank you again for the constructive handling of our manuscript throughout the review process and appreciate the opportunity to publish our work in *EMBO Reports*.

Nicolas Bidère

Dr. Nicolas Bidere
INSERM
INSERM U1307
Bât IRS-UN
8 quai Moncousu
Nantes 44200
France

Dear Nicolas,

Thank you for the final changes and clarifications. I am now very pleased to accept your manuscript for publication in the next available issue of EMBO reports. Thank you for your contribution to our journal.

Your manuscript will be processed for publication by EMBO Press. It will be copy edited and you will receive page proofs prior to publication. Please note that you will be contacted by Springer Nature Author Services to complete licensing and payment information.

You may qualify for financial assistance for your publication charges - either via a Springer Nature fully open access agreement or an EMBO initiative. Check your eligibility: https://link.springer.com/journal/44319/how-to-publish-with-us

Should you be planning a Press Release on your article, please notify the production team via this form in order to coordinate publication and release dates: https://forms.cloud.microsoft/e/eH4A8JVP6g

If you have any questions, please do not hesitate to contact the Editorial Office. Thank you for your contribution to EMBO Reports.

Kind regards,

Martina

Martina Rembold, PhD
Senior Editor
EMBO reports

------------------------------------------------

>>> Please note that it is EMBO Reports policy for the transcript of the editorial process (containing referee reports and your response letter) to be published as an online supplement to each paper. If you do NOT want this, you will need to inform the Editorial Office via email immediately. More information is available here: https://link.springer.com/partners/embo-press/editorial-policies#Peer%20review

## EMBO Press Author Checklist

| | |
|---|---|
| Corresponding Author Name: BIDERE | |
| Journal Submitted to: EMBO Reports | |
| Manuscript Number: EMBOR-2025-63467V1 | |

**USEFUL LINKS FOR COMPLETING THIS FORM**
The EMBO Journal - Author Guidelines
EMBO Reports - Author Guidelines
Molecular Systems Biology - Author Guidelines
EMBO Molecular Medicine - Author Guidelines

**Reporting Checklist for Life Science Articles (updated January**
This checklist is adapted from Materials Design Analysis Reporting (MDAR) Checklist for Authors. MDAR establishes a minimum set of requirements in transparent reporting in the life sciences (see Statement of Task: 10.31222/osf.io/9sm4x). Please follow the journal's guidelines in preparing your
**Please note that a copy of this checklist will be published alongside your article.**

**Abridged guidelines for figures**
**1. Data**
The data shown in figures should satisfy the following conditions:
- ➞ the data were obtained and processed according to the field's best practice and are presented to reflect the results of the experiments in an accurate and unbiased manner.
- ➞ ideally, figure panels should include only measurements that are directly comparable to each other and obtained with the same assay.
- ➞ plots include clearly labeled error bars for independent experiments and sample sizes. Unless justified, error bars should not be shown for technical
- ➞ if n<5, the individual data points from each experiment should be plotted. Any statistical test employed should be justified.
- ➞ Source Data should be included to report the data underlying figures according to the guidelines set out in the authorship guidelines on Data

**2. Captions**
Each figure caption should contain the following information, for each panel where they are relevant:
- ➞ a specification of the experimental system investigated (eg cell line, species name).
- ➞ the assay(s) and method(s) used to carry out the reported observations and measurements.
- ➞ an explicit mention of the biological and chemical entity(ies) that are being measured.
- ➞ an explicit mention of the biological and chemical entity(ies) that are altered/varied/perturbed in a controlled manner.
- ➞ the exact sample size (n) for each experimental group/condition, given as a number, not a range;
- ➞ a description of the sample collection allowing the reader to understand whether the samples represent technical or biological replicates (including how many animals, litters, cultures, etc.).
- ➞ a statement of how many times the experiment shown was independently replicated in the laboratory.
- ➞ definitions of statistical methods and measures:
  - common tests, such as t-test (please specify whether paired vs. unpaired), simple χ2 tests, Wilcoxon and Mann-Whitney tests, can be unambiguously identified by name only, but more complex techniques should be described in the methods section;
  - are tests one-sided or two-sided?
  - are there adjustments for multiple comparisons?
  - exact statistical test results, e.g., P values = x but not P values < x;
  - definition of 'center values' as median or average;
  - definition of error bars as s.d. or s.e.m.

**Please complete ALL of the questions below.**
**Select "Not Applicable" only when the requested information is not relevant for your study.**

**Materials**

| Newly Created Materials | Information included in the manuscript? | In which section is the information available? (Reagents and Tools Table, Materials and Methods, Figures, Data Availability Section) |
|---|---|---|
| New materials and reagents need to be available; do any restrictions apply? | Not Applicable | Reagent and Tools Table, Material and Methods |

| Antibodies | Information included in the manuscript? | In which section is the information available? (Reagents and Tools Table, Materials and Methods, Figures, Data Availability Section) |
|---|---|---|
| For **antibodies** provide the following information:<br>- Commercial antibodies: RRID (if possible) or supplier name, catalogue number and or/clone number<br>- Non-commercial: RRID or citation | Yes | Reagent and Tools Table, Material and Methods |

| DNA and RNA sequences | Information included in the manuscript? | In which section is the information available? (Reagents and Tools Table, Materials and Methods, Figures, Data Availability Section) |
|---|---|---|
| **Short novel DNA or RNA including primers, probes**: provide the sequences. | Yes | Reagent and Tools Table, Material and Methods |

| Cell materials | Information included in the manuscript? | In which section is the information available? (Reagents and Tools Table, Materials and Methods, Figures, Data Availability Section) |
|---|---|---|
| **Cell lines:** Provide species information, strain. Provide accession number in repository **OR** supplier name, catalog number, clone number, and/**OR** RRID. | Yes | Reagent and Tools Table, Material and Methods |
| **Primary cultures:** Provide species, strain, sex of origin, genetic modification status. | Not Applicable | |
| Report if the cell lines were recently **authenticated** (e.g., by STR profiling) and tested for mycoplasma contamination. | Yes | Matherials and Methods |

| Experimental animals | Information included in the manuscript? | In which section is the information available? (Reagents and Tools Table, Materials and Methods, Figures, Data Availability Section) |
|---|---|---|
| **Laboratory animals or Model organisms:** Provide species, strain, sex, age, genetic modification status. Provide accession number in repository **OR** supplier name, catalog number, clone number, **OR** RRID. | Not Applicable | |
| **Animal observed in or captured from the field:** Provide species, sex, and age where possible. | Not Applicable | |
| Please detail **housing and husbandry conditions**. | Not Applicable | |

| Plants and microbes | Information included in the manuscript? | In which section is the information available? (Reagents and Tools Table, Materials and Methods, Figures, Data Availability Section) |
|---|---|---|
| **Plants:** provide species and strain, ecotype and cultivar where relevant, unique accession number if available, and source (including location for collected wild specimens). | Not Applicable | |
| **Microbes:** provide species and strain, unique accession number if available, and source. | Not Applicable | |

| Human research participants | Information included in the manuscript? | In which section is the information available? (Reagents and Tools Table, Materials and Methods, Figures, Data Availability Section) |
|---|---|---|
| If collected and within the bounds of privacy constraints report on age, sex and gender or ethnicity for all study participants. | Not Applicable | |

| Core facilities | Information included in the manuscript? | In which section is the information available? (Reagents and Tools Table, Materials and Methods, Figures, Data Availability Section) |
|---|---|---|
| If your work benefited from core facilities, was their service mentioned in the acknowledgments section? | Yes | Materials and Methods, Ackowledgments section |

**Design**

| Study protocol | Information included in the manuscript? | In which section is the information available? (Reagents and Tools Table, Materials and Methods, Figures, Data Availability Section) |
|---|---|---|
| If study protocol has been **pre-registered, provide DOI in the manuscript**. For clinical trials, provide the trial registration number **OR** cite DOI. | Not Applicable | |
| Report the **clinical trial registration number** (at ClinicalTrials.gov or equivalent), where applicable. | Not Applicable | |

| Laboratory protocol | Information included in the manuscript? | In which section is the information available? (Reagents and Tools Table, Materials and Methods, Figures, Data Availability Section) |
|---|---|---|
| Provide DOI OR other citation details if **external detailed step-by-step protocols** are available. | Not Applicable | |

| Experimental study design and statistics | Information included in the manuscript? | In which section is the information available? (Reagents and Tools Table, Materials and Methods, Figures, Data Availability Section) |
|---|---|---|
| Include a statement about **sample size** estimate even if no statistical methods were used. | Yes | Figure legends |
| Were any steps taken to minimize the effects of subjective bias when allocating animals/samples to treatment (e.g. **randomization procedure**)? If yes, have they been described? | Not Applicable | |
| Include a statement about **blinding** even if no blinding was done. | Not Applicable | |
| Describe **inclusion/exclusion criteria** if samples or animals were excluded from the analysis. Were the criteria pre-established?<br><br>If sample or data points were omitted from analysis, report if this was due to attrition or intentional exclusion and provide justification. | Not Applicable | |
| For every figure, are **statistical tests** justified as appropriate? Do the data meet the assumptions of the tests (e.g., normal distribution)? Describe any methods used to assess it. Is there an estimate of variation within each group of data? Is the variance similar between the groups that are being statistically compared? | Yes | Figure legends |

| Sample definition and in-laboratory replication | Information included in the manuscript? | In which section is the information available? (Reagents and Tools Table, Materials and Methods, Figures, Data Availability Section) |
|---|---|---|
| In the figure legends: state number of times the experiment was **replicated** in laboratory. | Yes | Figure legends |
| In the figure legends: define whether data describe **technical or biological replicates**. | Yes | Figure legends |

## Ethics

| Ethics | Information included in the manuscript? | In which section is the information available? (Reagents and Tools Table, Materials and Methods, Figures, Data Availability Section) |
|---|---|---|
| Studies involving **human participants**: State details of **authority granting ethics approval** (IRB or equivalent committee(s), provide reference number for approval. | Not Applicable | |
| Studies involving **human participants**: Include a statement confirming that **informed consent** was obtained from all subjects and that the experiments conformed to the principles set out in the WMA Declaration of Helsinki and the Department of Health and Human Services Belmont Report. | Not Applicable | |
| Studies involving **human participants:** For publication of **patient photos**, include a statement confirming that consent to publish was obtained. | Not Applicable | |
| Studies involving experimental **animals**: State details of **authority granting ethics approval** (IRB or equivalent committee(s), provide reference number for approval. Include a statement of compliance with ethical regulations. | Not Applicable | |
| Studies involving **specimen and field samples:** State if relevant **permits** obtained, provide details of authority approving study; if none were required, explain why. | Not Applicable | |

| Dual Use Research of Concern (DURC) | Information included in the manuscript? | In which section is the information available? (Reagents and Tools Table, Materials and Methods, Figures, Data Availability Section) |
|---|---|---|
| Could your study fall under dual use research restrictions? Please check biosecurity documents and list of **select agents and toxins** (CDC): https://www.selectagents.gov/sat/list.htm | Not Applicable | |
| If you used a select agent, is the security level of the lab appropriate and reported in the manuscript? | Not Applicable | |
| If a study is subject to dual use research of concern regulations, is the name of the **authority granting approval and reference number** for the regulatory approval provided in the manuscript? | Not Applicable | |

## Reporting

The MDAR framework recommends adoption of discipline-specific guidelines, established and endorsed through community initiatives. Journals have their own policy about requiring specific guidelines and recommendations to complement MDAR.

| Adherence to community standards | Information included in the manuscript? | In which section is the information available? (Reagents and Tools Table, Materials and Methods, Figures, Data Availability Section) |
|---|---|---|
| State if relevant guidelines or checklists (e.g., **ICMJE, MIBBI, ARRIVE, PRISMA**) have been followed or provided. | Not Applicable | |
| For **tumor marker prognostic studies,** we recommend that you follow the **REMARK** reporting guidelines (see link list at top right). See author guidelines, under 'Reporting Guidelines'. Please confirm you have followed these guidelines. | Not Applicable | |
| For **phase II and III randomized controlled trials**, please refer to the **CONSORT** flow diagram (see link list at top right) and submit the CONSORT checklist (see link list at top right) with your submission. See author guidelines, under 'Reporting Guidelines'. Please confirm you have submitted this list. | Not Applicable | |

## Data Availability

| Data availability | Information included in the manuscript? | In which section is the information available? (Reagents and Tools Table, Materials and Methods, Figures, Data Availability Section) |
|---|---|---|
| Have **primary datasets** been deposited according to the journal's guidelines (see 'Data Deposition' section) and the respective accession numbers provided in the Data Availability Section? | Not Applicable | |
| Were **human clinical and genomic datasets** deposited in a public access-controlled repository in accordance to ethical obligations to the patients and to the applicable consent agreement? | Not Applicable | |
| Are **computational models** that are central and integral to a study available without restrictions in a machine-readable form? Were the relevant accession numbers or links provided? | Not Applicable | |
| If publicly available data were reused, provide the respective **data citations in the reference list**. | Not Applicable | |
